# Quantitative imaging reveals the role of MpARF proteasomal degradation during gemma germination

**DOI:** 10.1016/j.xplc.2024.101039

**Published:** 2024-07-09

**Authors:** Shubhajit Das, Martijn de Roij, Simon Bellows, Melissa Dipp Alvarez, Sumanth Mutte, Wouter Kohlen, Etienne Farcot, Dolf Weijers, Jan Willem Borst

**Affiliations:** 1 Laboratory of Biochemistry, Wageningen University and Research, Stippeneng 4, 6708WE Wageningen, the Netherlands; 2 School of Mathematical Sciences, University of Nottingham, University Park, NG7 2RD Nottingham, UK; 3 Laboratory of Molecular Biology, Wageningen University and Research, Droevendaalsesteeg 1, 6708PB Wageningen, the Netherlands

**Keywords:** auxin signaling, ARF degradation, *Marchantia polymorpha*, live cell imaging

## Abstract

The auxin signaling molecule controls a variety of growth and developmental processes in land plants. Auxin regulates gene expression through a nuclear auxin signaling pathway (NAP) consisting of the ubiquitin ligase auxin receptor TIR1/AFB, its Aux/IAA degradation substrate, and DNA-binding ARF transcription factors. Although extensive qualitative understanding of the pathway and its interactions has been obtained, mostly by studying the flowering plant *Arabidopsis thaliana*, it remains unknown how these translate to quantitative system behavior *in vivo*, a problem that is confounded by the large NAP gene families in most species. Here, we used the minimal NAP of the liverwort *Marchantia polymorpha* to quantitatively map NAP protein accumulation and dynamics *in vivo* through the use of knockin fluorescent fusion proteins. Beyond revealing the dynamic native accumulation profile of the entire NAP protein network, we discovered that the two central ARFs, MpARF1 and MpARF2, are proteasomally degraded. This auxin-independent degradation tunes ARF protein stoichiometry to favor gene activation, thereby reprogramming auxin response during the developmental progression. Thus, quantitative analysis of the entire NAP has enabled us to identify ARF degradation and the stoichiometries of activator and repressor ARFs as a potential mechanism for controlling gemma germination.

## Teaser

The *Marchantia* auxin response system is the simplest found in land plants. This study reports the quantitative imaging of a set of genomic knockin fluorescent fusions, that reveals native protein accumulation patterns and the regulated degradation of DNA-binding ARF proteins. Proteolysis tunes the stoichiometry of positively and negatively acting ARFs, thereby reprogramming the auxin response during development.

## Introduction

**Figure 1:**
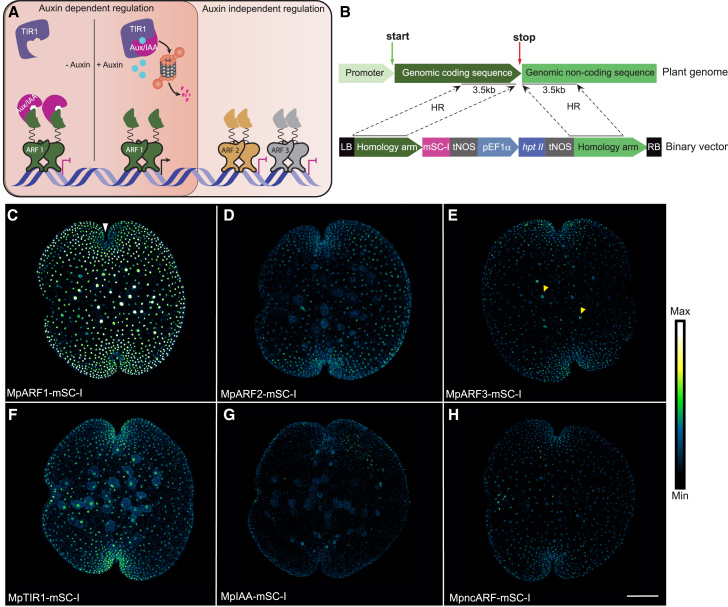
Native cellular accumulation patterns of NAP signaling proteins in *Marchantia* gemmae. **(A)** Schematic diagram of the nuclear auxin signaling pathway. In the absence of auxin, Aux/IAA repressors interact with ARFs to inhibit transcriptional activation. In the presence of auxin, TIR1 interacts with Aux/IAA and targets it for proteasomal degradation. Free ARFs initiate gene transcription of auxin response genes. ARF2 is independent from this regulation. The regulation mode of ARF3 is not well characterized. Both ARF2 and ARF3 act as transcriptional repressors.**(B)** Principle of homologous recombination (HR)-mediated genomic knockin of fluorescent proteins at the C terminus of a gene of interest. *hptII*, hygromycin phosphotransferase; LB, T-DNA left border; mSC-I, mScarlet-I; pEF1ɑ, ELONGATION FACTOR1ɑ promoter; tNOS, nopaline synthase terminatormoter; RB, T-DNA right border; tNOS, nopaline synthase terminator.**(C–H)** Fluorescence patterns reflecting the accumulation of core nuclear auxin signaling proteins in dormant gemmae of *Marchantia* knockin lines with mScarlet-I. Scale bar, 100 μm. White arrowhead indicates the apical notch; yellow arrowheads indicate the rhizoid cells.

The plant signaling molecule auxin triggers a multitude of growth, developmental, and physiological responses across land plants (Teale et al., 2006; Vanneste and Friml, 2009). Key to the cellular response is a canonical nuclear auxin signaling pathway (NAP) (Lavy and Estelle, 2016). Auxin promotes the binding of AUXIN/INDOLE-3-ACETIC ACID (Aux/IAA) repressor proteins to the nuclear auxin receptor TRANSPORT INHIBITOR RESPONSE1/AUXIN SIGNALING F-BOX (TIR1/AFB) (Tan et al., 2007). This triggers degradation of Aux/IAA proteins (Gray et al., 2001) and releases DNA-binding AUXIN RESPONSE FACTORS (ARFs) (Weijers and Wagner, 2016; Leyser, 2018) from inhibition (Figure 1A). In past decades, the NAP has been studied extensively in the angiosperm *Arabidopsis thaliana* (Friml, 2022). These studies have led to a comprehensive qualitative model of auxin signaling, supported by atomic structures of each component. A key challenge in understanding auxin response is the large size of gene families representing each component in angiosperms (Luo et al., 2018). Specifically, it is hard to tell whether any one protein behaves typically or atypically. More recent analysis of the auxin response system in the bryophytes *Physcomitrium patens* (a moss) and *Marchantia polymorpha* (a liverwort) has helped to reduce system complexity and derive common core principles. Phylogenetic analysis has shown that the simple NAP system in *Marchantia* consists of one ARF in each of the three subclasses (A class: MpARF1; B class: MpARF2; C class: MpARF3), a single Aux/IAA (MpIAA), and a single TIR1 receptor (MpTIR1), in addition to a non-canonical ARF (MpncARF) and a non-canonical Aux/IAA (MpncIAA; Mutte et al., 2018). Studies in *Physcomitrium* and *Marchantia* led to the formulation of a model in which the auxin response revolves around antagonistic interactions between A- and B-class ARFs competing for the same DNA-binding sites. While A-ARFs are regulated by auxin and can switch between repression and activation, B-ARFs are auxin-independent repressors (Lavy et al., 2016; Kato et al., 2020). Thus, stoichiometry of the A- and B-class ARFs is predicted to determine the output of auxin response. The C-class ARF in *Marchantia* acts as an auxin-independent transcriptional repressor (Flores-Sandoval et al., 2018), and the non-canonical ARF (ncARF) that lacks the DNA-binding domain positively influences auxin response (Mutte et al., 2018). However, the mechanisms of C-class ARF DNA binding and ncARF-driven auxin response remain to be explored.

Despite our deep understanding of the NAP, one key element remains largely unresolved: essentially all *in vivo* findings are built on qualitative data, and the true *in vivo* concentrations and accumulation patterns of NAP components and their relative concentrations or stoichiometries are unknown. As a consequence, it is unclear whether domains of different auxin activities are generated and, if so, through what mechanisms. Likewise, it is nearly impossible to infer dynamics from genetic or *in vitro* biochemical data, which thus leaves many questions unanswered about the temporal aspects of auxin response. Given that any biochemical interaction is determined by both affinity and concentration of and among components, it will be essential to map protein patterns *in vivo*. The ability to tag endogenous proteins through homologous-recombination-based gene targeting in bryophytes enables us to visualize the dynamics of proteins at native concentrations, as a first step toward understanding system behavior.

In this study, we generated and characterized fluorescent genomic knockin lines for all *Marchantia* NAP proteins. In addition to resolving spatial and temporal maps of protein accumulation, this enabled us to identify active proteasomal degradation of both MpARF1 and MpARF2. We show that degradation tunes A-/B-ARF stoichiometry to permit normal development and auxin response. Our study provides a unique resource for the quantitative analysis of auxin response in *Marchantia* and reveals proteasomal degradation as a regulatory mechanism for ARFs.

## Results

### Development of a collection of genomic knockin lines for the *Marchantia* NAP

Essentially all activities and properties underlying protein function are dependent upon protein concentration. Therefore, a quantitative understanding of any biological process requires the monitoring of endogenous protein accumulation patterns, which can be achieved either by immunolabeling—which suffers from difficulties in reproducible quantification across different proteins—or by endogenous tagging. The latter has so far not been feasible in flowering plants because of the extremely low efficiencies of homology-directed gene targeting, but it is realistic in bryophytes. We set out to generate genomic knockin translational fluorescent fusion lines for all components of the NAP in *Marchantia* (Figure 1B) (Ishizaki et al., 2013). We previously generated knockin lines for both MpARF1 and MpARF2, fused to mScarlet-I (Kato et al., 2020). We extended this, and using homologous recombination, we knocked in a fluorescent protein (either mNeonGreen or mScarlet-I) at the C terminus of each auxin signaling protein: MpARF1 (class A), MpARF2 (class B), MpARF3 (class C), MpIAA, MpTIR1, and MpncARF, thus effectively replacing the single endogenous copy with a fluorescently tagged one.

Where analyzed, overexpression and loss of function of these proteins have been shown to cause strong phenotypes (Flores-Sandoval et al., 2015; Kato et al., 2015, 2017, 2020; Mutte et al., 2018; Suzuki et al., 2023) In contrast, all knockin lines displayed macroscopically normal morphologies and responded to externally applied auxin in a manner qualitatively similar to that of the wild type (Supplemental Figure 1). This suggests that all fusion proteins are functional and that none accumulate at levels significantly outside of the normal range. Next, we used confocal microscopy to visualize all auxin signaling proteins in dormant gemmae residing within the gemma cup, and we compared fusions of all NAP components to the same fluorescent protein (mScarlet-I) under identical experimental conditions. All three ARFs localized to nuclei, but their tissue-wide accumulation patterns showed clear differences (Figures 1C–1E). MpARF1-mScarlet-I showed the highest accumulation among all the NAP components and was present in most cell types of the gemma (see explanation of different cell types in Supplemental Figure 2). MpARF2-mScarlet-I accumulation was much weaker and was absent in the region distal of the apical notch where rhizoid initials reside. MpARF3-mScarlet-I (class C) displayed a weak yet broad accumulation pattern in all cell types. The auxin receptor MpTIR1-mScarlet-I and the non-canonical MpncARF-mScarlet-I showed low, nuclear accumulation, and MpIAA-mScarlet-I was not detected at this stage (Figures 1F–1H). In summary, we established a collection of knockin lines that enabled mapping and quantification of NAP protein accumulation patterns *in vivo*.

### Co-receptor-ligand dynamics in *Marchantia* NAP

**Figure 2:**
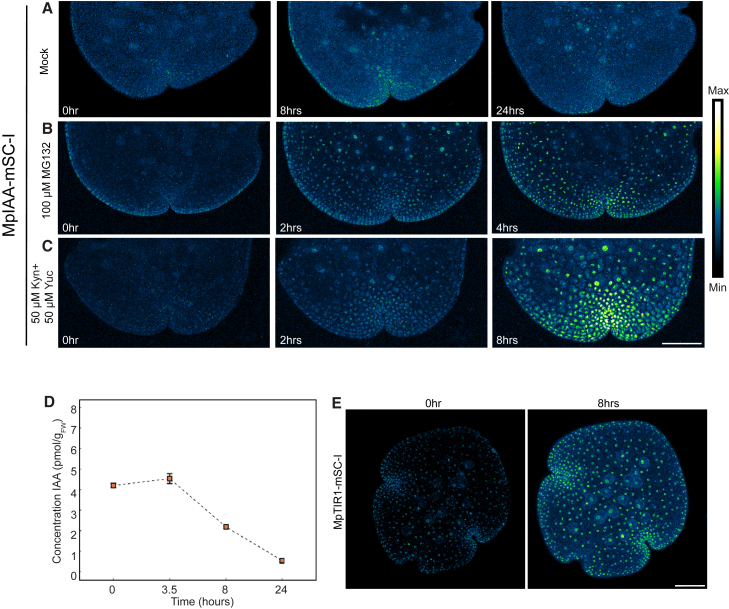
Concentrations of auxin and MpIAA are dynamic during gemma development. **(A–C)** Time-course imaging of MpIAA-mSC-I treated with mock **(A)**, 100 μM MG132 **(B)**, and 50 μM l-kynurenine and 50 μM yucasin **(C)**.(**D)** Concentration dynamics of free IAA in germinating gemmae (*n* = 3, error bars: SD). Liquid chromatography–mass spectrometry quantification shows that total IAA levels drop following gemma germination.(**E)** Time-course imaging of MpTIR1-mSC-I shows that its accumulation levels gradually increase during gemma germination. Scale bars, 100 μm.

We could not detect MpIAA in dormant gemma (Figure 1G), and therefore we monitored MpIAA accumulation patterns in the first 24 h following gemma germination. The first weak nuclear signals could be detected after 24 h of growth (Figure 2A). It is conceivable that the MpIAA protein is expressed in dormant gemmae but continuously degraded owing to high auxin concentrations. Indeed, treatment of dormant gemmae with the proteasome inhibitor MG132 led to a clear nuclear signal within 2 h (Figure 2B). By quantifying free IAA concentrations in germinating gemmae, we found a clear decline in the 24 h following germination (Figure 2D), consistent with IAA-triggered MpIAA degradation at early stages. Pharmacological inhibition of auxin synthesis by combining l-kynurenine and yucasin inhibitors (He et al., 2011; Nishimura et al., 2014) led to abundant nuclear MpIAA-mScarlet-I accumulation across gemmae within 2 h (Figure 2C). These findings suggest that MpTIR1 is active throughout gemma development in mediating IAA-triggered MpIAA degradation. MpTIR1-mScarlet-I was detectable at all stages and showed a gradual increase in protein levels as gemmae grew (Figure 2E). Thus, as gemmae break dormancy and grow, the auxin response system moves from restrictive to permissive.

**Figure 3:**
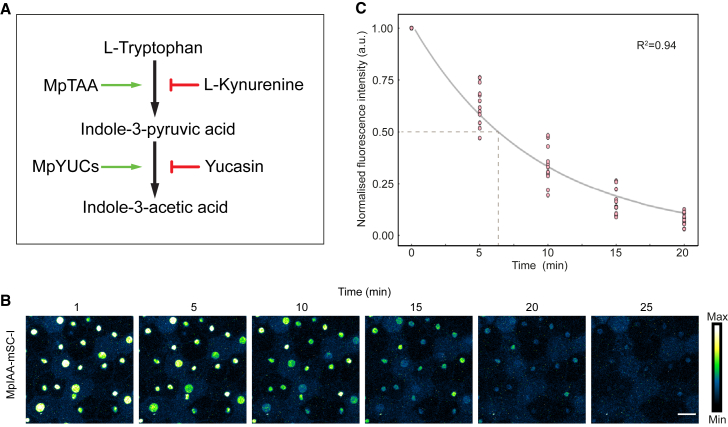
Quantification of MpIAA turnover. **(A)** Auxin biosynthesis via the indole pyruvic acid pathway is catalyzed by two key enzymes, TRYPTOPHAN AMINOTRANSFERASE OF ARABIDOPSIS (TAA1) and YUCCA (YUC). To lower auxin levels in a tissue, these enzymes can be chemically inhibited by l-kynurenine and yucasin, respectively.(**B)** Time-lapse imaging of MpIAA-mSC-I gemmae pre-treated with 50 μM l-kynurenine and 50 μM yucasin. Upon auxin (1-NAA; 3 μM) treatment, fluorescence rapidly decreases owing to MpIAA-mSC-I degradation.**(C)** Fitting normalized fluorescence data using a 1-component exponential model results in an MpIAA half-life of 6.5 min. Scale bar, 25 μm.

The pace of auxin signal relay from TIR1/AFBs to ARFs depends on the degradation rate of Aux/IAA proteins. A quick transcriptional response to auxin, therefore, relies on the rapid degradation of Aux/IAAs (Dreher et al., 2006). Aux/IAA degradation rates in *Arabidopsis*, where studied, range from 10 min to 1.3 h, and it is unclear what rate reflects the ancestral state within the Aux/IAA family. To determine the *in vivo* MpIAA half-life, we first depleted endogenous auxin with l-kynurenine and yucasin (Figure 3A). This led to abundant nuclear accumulation of MpIAA. Gemmae were next treated with 3 μM of the stable, synthetic auxin 1-NAA (1-naphthaleneacetic acid), and MpIAA protein levels were tracked and quantified by time-lapse imaging (Figure 3B). We detected a clear loss of fluorescence signal and determined the half-life of MpIAA to be 6.5 min (Figure 3C). To confirm that the signal decline was due to proteasomal degradation, we added MG132 and found that the fluorescence signal remained over the time course (Supplemental Figure 3). Likewise, the signal of MpARF1-Scarlet-I did not decline over the same time with the same imaging settings (Supplemental Figure 4). This demonstrated that the imaging settings used did not induce significant photobleaching. These results suggest that the ancestral condition of the NAP may be characterized by fast Aux/IAA turnover and that the slower degradation rates found in *Arabidopsis* may be a derived property.

### Dynamic MpARF protein accumulation

**Figure 4:**
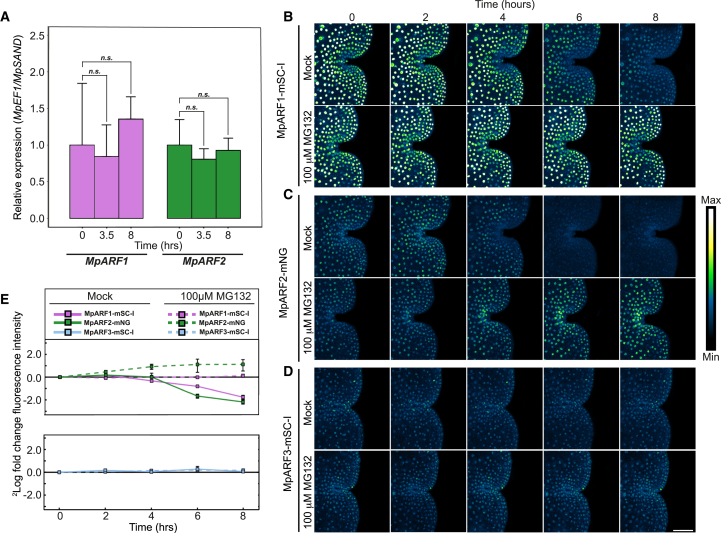
MpARF protein accumulation in gemmae is post-translationally regulated by the proteasome. **(A)** qRT–PCR of *MpARF1* and *MpARF2* transcripts shows stable gene transcription during gemma germination (*n* = 3, Student’s *t* test *p* < 0.05).**(B–D)** Time-course imaging of MpARF1, MpARF2, and MpARF3 protein fusions to mScarlet-I in germinating gemmae in the control treatment or upon treatment with 100 μM MG132.**(E)** Quantification of MpARF1, MpARF2, and MpARF3 protein accumulation after germination, expressed as FC relative to that in dormant gemmae (≥30 nuclei quantified per image, *n* = 3, error bars: SE). Scale bar, 50 μm.

Dynamics in the NAP are ascribed primarily to the degradation of Aux/IAA proteins, whereas TIR1 and ARFs are considered to be more stable factors. Indeed, transcriptional levels of MpARFs were stable following gemma germination (Figure 4A). To explore the accumulation dynamics of the MpARF proteins, we imaged each ARF during the first 24 h following germination. Remarkably, we found that both MpARF1 and MpARF2 protein levels progressively declined (Figures 4B, 4C, and Supplemental Figure 5). By contrast, MpARF3 patterns did not change in this time window (Figure 4D). Given the higher starting levels of MpARF1 in dormant gemmae and the lower MpARF2 concentrations, the former was still detectable after 8 h, whereas the MpARF2 signal declined to undetectable levels (Supplemental Figure 5A). Comparable dynamics were found for MpARF1-mNeonGreen and MpARF2-mNeonGreen (Supplemental Figure 5), indicating that signal decline was independent of the fluorescent protein tag.

Given that MpARF transcript levels did not change in the same time window (Figure 4A), MpARF1 and MpARF2 must be post-transcriptionally controlled by either translational or post-translational regulation. To test the involvement of proteolysis, we treated plants with the proteasome inhibitor MG132 or bortezomib. While MpARF3 was unaffected by the treatments (Figures 4D and 4E), the decline of MpARF1 was prevented by either inhibitor (Figures 4B, 4C, 4E, and Supplemental Figure 6). MpARF2 levels not only stabilized upon treatment with the inhibitors but even increased relative to those of dormant gemmae (Figures 3B, 3C, 3E, and Supplemental Figure 6). This implies that both MpARF1 and MpARF2, but not MpARF3, are proteasomally degraded.

Proteolysis of regulatory proteins, including transcriptional regulators, is often part of feedback control or a point of regulation in signaling pathways. To investigate whether auxin or any other known signaling molecule triggers MpARF degradation during germination, we exogenously treated gemmae for ±16 h with auxin and a range of other plant hormones or inhibitors. Neither auxin nor abscisic acid, gibberellic acid, or jasmonic acid altered MpARF degradation. Similarly, auxin transport or biosynthesis inhibitors did not affect degradation (Supplemental Figure 7), suggesting that ARF degradation is not controlled by auxin or other tested hormones.

### Regulated degradation controls ARF stoichiometry for normal development and auxin response

**Figure 5:**
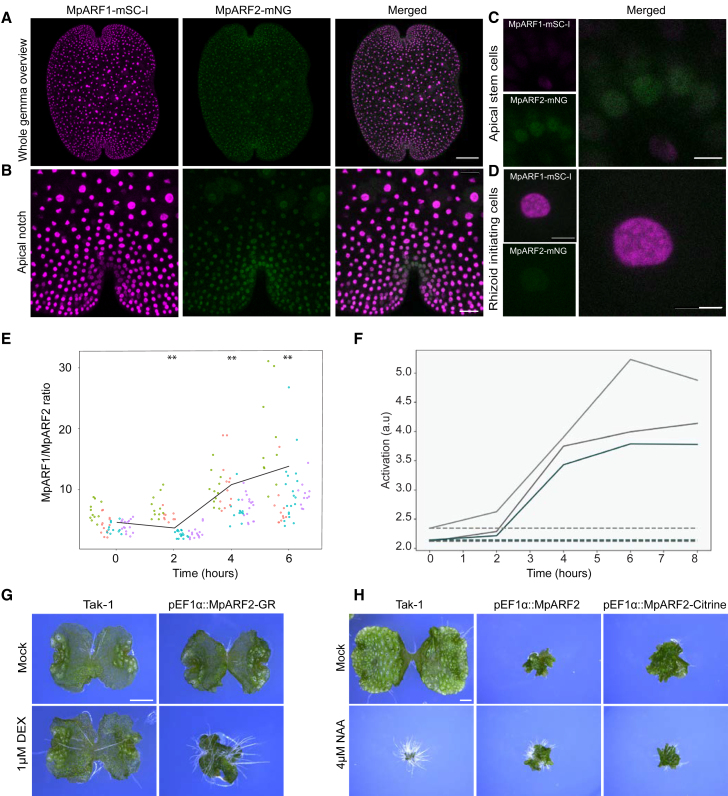
Control of gemma growth by regulation of MpARF1/MpARF2 stoichiometry. **(A)** Overview of MpARF1-mSC-I and MpARF2-mNG accumulation patterns in dormant gemmae of double knockin lines (scale bar, 100 μm).**(B–D)** Detail of MpARF1-mSC-I and MpARF2-mNG accumulation patterns in the apical notch region (**B**; scale bar, 25 μm), outermost apical notch cells (**C**; scale bar, 5 μm), and rhizoid initial cells (**D**; scale bar, 5 μm).**(E)** Quantification of ARF1:ARF2 stoichiometry in individual nuclei of a double knockin line (MpARF1-mScI MpARF2-mNG) during gemma germination. Colors mark nuclei from different gemmae. ∗∗*p* < 0.05, paired Student’s *t* test between t = 0 and other time points.**(F)** Predicted transcription pattern of an auxin-inducible gene (a.u.) during gemma germination. Solid lines indicate transcription rate under normal conditions in which MpARFs degrade during gemma germination, whereas dashed lines indicate transcription rate in the absence of MpARF2 proteasomal degradation. Lines correspond to predictions modeled in 3 replicate measurements of ARF levels.**(G)** Phenotypes of wild-type and pEF1ARF2-GR lines treated for 7 days with 1 μM dexamethasone or mock control.**(H)** Phenotypes of 14-day-old wild type and untagged (pEF1αARF2) or citrine-tagged (pEF1αARF2-citrine) constitutive ARF2 overexpression lines grown on mock medium or medium containing 4 μM 1-NAA. Scale bars, 1 mm.

A key question is what function is served by the regulated degradation of MpARF1 and MpARF2. We previously proposed that auxin responsiveness in *Marchantia* is determined by the stoichiometry of MpARF1 and MpARF2 (Kato et al., 2020). We therefore explored the possibility that this stoichiometry is actively regulated by proteolysis. Because such stoichiometries may differ among individual cells, it is not possible to derive them by comparing individual knockin lines. To visualize both MpARF1 and MpARF2 quantities in the same tissue and in the same cell, we crossed MpARF1-mScarlet-I with MpARF2-mNeonGreen and MpARF1-mNeonGreen with MpARF2-mScarlet-I and obtained two double knockin lines. Both double knockins showed ARF accumulation patterns comparable to those of the single MpARF lines (Figures 4B, 4C, and 5A–5C). As expected from their individual patterns, MpARF1 and MpARF2 were present in different stoichiometries in different cell types. In nuclei of meristematic apical notch cells, MpARF2 displayed higher accumulation than MpARF1 (Figure 5A–5C). In rhizoid initial cells, MpARF2 protein was nearly undetectable, whereas MpARF1 showed clear accumulation (Figure 5D). In the region separating the apical notch and the rhizoid initial cells, which we refer to here as the transition zone, both MpARFs were present (Figure 5A and 5B and Supplemental Figure 8).

We next determined MpARF1/MpARF2 stoichiometries following gemma germination in an MpARF1-mSc-I/MpARF2-mNG double knockin line. For this analysis, we measured relative MpARF1:MpARF2 fluorescence intensities in individual nuclei within four independent gemmae at four time points. Quantification of the MpARF1 and 2 fluorescence intensities in the transition zone showed that the MpARF1:MpARF2 stoichiometry consistently increased during gemmae germination, favoring MpARF1 over MpARF2 (Figure 5E).

We next asked what the impact of this change in MpARF1:MpARF2 stoichiometry might be, and we first developed a mathematical model of the minimal *Marchantia* NAP and its known interactions (Supplemental File 1). Using a piecewise polynomial function, we fit three independent, experimentally quantified MpARF accumulation profiles into the mathematical model, in which the effect of changing MpARF1 and MpARF2 levels on the outcome of auxin response was modeled as the transcription of a hypothetical ARF-regulated gene. Model simulations predicted that the increased MpARF1:MpARF2 ratio would enhance transcriptional response output (solid lines, Figure 5F). Simulated loss of MpARF2 degradation predicted no response output (dashed lines, Figure 5F). Thus, ARF degradation may be required to switch auxin-regulated genes from an inactive to an active transcriptional state by acting on the MpARF1:MpARF2 stoichiometry.

To test the prediction that regulated, low MpARF2 levels are needed for proper auxin sensitivity, we made use of the published MpARF2 inducible overexpression lines (Kato et al., 2020). In addition, we generated a new set of stable overexpression lines with and without a C-terminal citrine tag. Induction of MpARF2-GR activity with dexamethasone prevented thallus growth (Figure 5G) (Jones and Dolan, 2012; Flores-Sandoval et al., 2015). Likewise, 2-week-old stable pEF1-MpARF2 overexpression lines had multiple apical notches, were strongly defective in growth, lacked gemma cups (Supplemental Figure 9), and were completely insensitive to auxin treatment (1-NAA; Figure 5H and Supplemental Figures 6A and 6B). The inducible ARF2-GR lines had a lower expression of auxin response genes (Kato et al., 2020). In addition, in previous reports, overexpression of MpARF1 has been shown to cause auxin hypersensitivity and increased branching rates (see Eklund et al., 2015; Kato et al., 2020). These results support the prediction that elevated levels of MpARF1 and MpARF2 have opposite effects on auxin sensitivity.

### Transcriptional reprogramming in germinating gemmae

**Figure 6:**
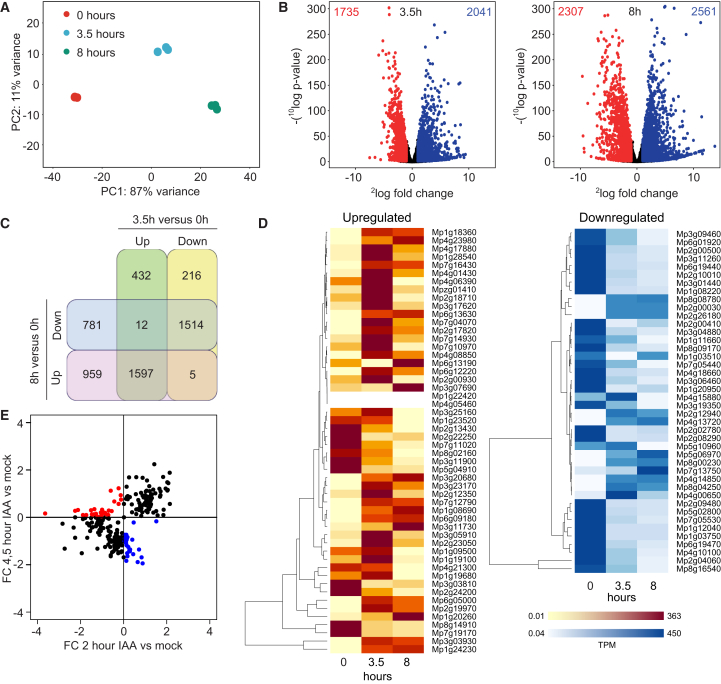
Reprogramming of transcriptional auxin response capacity during early gemma development. **(A)** PCA plot of RNA-seq transcriptomes of dormant gemmae and gemmae grown in control medium for 3.5 and 8 h. **(B)** Volcano plots show the number of significantly upregulated (blue) and downregulated (red) genes (padj < 0.05) at 3.5 and 8 h relative to the 0-h time point.**(C)** Venn diagram illustrates the overlap of DEGs in 3.5- and 8-h gemmae.**(D)** Transcriptional dynamics (TPM) of auxin-responsive genes during gemma germination.**(E)** Plot of log_2_(FC) differently regulated genes between 2- and 4.5-h-old gemmae treated with mock medium or auxin. Plot shows FC in average TPM counts of all differentially regulated genes at the 2 time points. Commonly regulated genes are marked in black; genes with opposite regulation at 2 and 4.5 h are indicated in blue and red, respectively.

The dynamics of NAP components, particularly the shift in ARF stoichiometry following gemma germination, suggest that auxin-regulated genes are dynamically controlled. To test this prediction directly, we performed an RNA-sequencing (RNA-seq) experiment on gemmae collected at 0 h (dormant), 3.5 h, and 8 h of growth. Principal-component analysis (PCA) resulted in a strong clustering of biological replicates and a high variance between different time points. This suggests major differences in the transcriptome at different developmental stages and implies massive transcriptional reprogramming early during gemma germination (Figure 6A). After 3.5 h of gemma growth, we found hundreds of differentially regulated genes, and this effect was even more pronounced after 8 h (Figure 6B). There was a large overlap in genes that were either up- or downregulated at 3, 5, and 8 h compared with the 0-h time point (Figure 6C). However, a substantial fraction of genes was also uniquely regulated at each time point (Figure 6C). We next identified 94 known auxin-regulated genes from available transcriptome data of auxin-treated wild-type plants (Mutte et al., 2018) and determined their expression levels across the time series. Transcript levels of the majority of auxin-upregulated genes were higher following germination, and likewise, transcript counts of most auxin-downregulated genes were lower in germinated gemmae (Figure 6D). Thus, as predicted, at a global scale, transcriptional auxin response is elevated upon dormancy release. However, there also seems to be a dampening of auxin-regulated gene expression at 8 h, which suggests that there is additional complexity at this time point that our model does not capture. To directly test the capacity for auxin-regulated gene expression at different times during gemma germination, we harvested gemmae either 1 or 3.5 h after removal from the gemma cup, treated each set separately with 1 μM IAA for 1 h, and then performed RNA-seq analysis. We determined differential expression upon IAA treatment at each time point and plotted the fold change (FC) for each gene at the two time points (Figure 6E). This analysis showed that among 289 auxin-regulated genes (152 at 1 h and 137 at 3.5 h), 26 showed similar regulation at both time points. Notably, there was no substantial feedback regulation of NAP components, including MpIAA (Supplemental Figure 10). Of the 26 genes with similar auxin regulation at both time points, a substantial fraction showed a clear difference in amplitude between time points. This result demonstrates that, as time passes after gemma germination, the capacity for auxin-regulated gene expression changes both qualitatively (identity of genes) and quantitatively (amplitude of regulation).

## Discussion

We used the simple, minimal auxin response system of *Marchantia* to map protein levels both quantitatively and qualitatively throughout gemma development. To this end, we generated a full set of genomic knockin lines in which fluorescent proteins (two spectrally unique fluorophores) were fused to the C terminus of each auxin signaling protein by homologous recombination. Such an effort would also be possible in *Physcomitrium patens*, given the ease of gene targeting in that species (Roberts et al., 2011), but it would be much more complicated owing to the larger gene families. We do not know of any other plant species that would currently allow such a holistic investigation of the auxin response system. Given their macroscopically normal phenotypes, we believe that these knockins faithfully represent the native spatiotemporal accumulation profile of all nuclear auxin signaling proteins in *Marchantia*.

A noticeable pattern in MpARF spatial expression was the cell-specific difference in ARF quantities. Cell types with either A-class ARF1, B-class ARF2, or both, were observed. Because class A and B ARFs can recognize the same DNA-binding element in *Arabidopsis*, *Physcomitrium*, and *Marchantia* (Boer et al., 2014; Freire-Rios et al., 2020), the relative concentrations of these two competing MpARFs should determine auxin responsiveness. The contrasting localization maps of MpARF1 and MpARF2 in various cell types satisfy the protein abundance requirements to support an ARF stoichiometry-dependent model of gene regulation (Supplemental Figure S8). The relatively high MpARF1 accumulation observed in rhizoid initial cells points toward an ARF1-dominated gene activation mode. In the apical cells, we observed the opposite pattern—relatively high MpARF2 abundance, suggesting a gene repression mode. A plausible explanation is that auxin levels are high in stem cells, resulting in the degradation of MpIAA and relieving any auxin-dependent repression. To keep these stem cells transcriptionally silent and possibly to maintain stem cell identity, MpARF2 expression is relatively high. In the transition zone, both MpARF1 and MpARF2 accumulate, suggesting that this region is defined by a competitive ARF-DNA binding mode. MpARF-DNA binding assays and transcriptome analysis at a single-cell resolution would help determine the actual ARF output at cellular resolution.

By following the localization of all NAP components over time, we characterized the dynamics of the MpTIR1 receptor, the MpIAA repressor, and IAA itself, which suggested a progressive transition from a state with high auxin, low receptor, and low MpIAA to one with low auxin, high receptor, and higher MpIAA. Thus, the capacity for MpIAA-dependent auxin response changes dynamically during development. It could be that the gemma cup itself provides a high-auxin environment, as proposed previously (Eklund et al., 2015), and causes effective Aux/IAA degradation in gemmae. The capacity to respond to auxin depends on both the TIR1–Aux/IAA machinery and the ARF transcription factors (Weijers and Wagner, 2016; Leyser, 2018). As auxin at high concentration is a growth inhibitory signal and is required to keep gemmae dormant inside gemma cups (see Eklund et al., 2015), a reduction in auxin is required to release dormancy and allow gemma growth. As MpARF1:MpARF2 stoichiometry increases post-germination, transcriptional responses are permitted at an auxin concentration that is more favorable for growth. While imaging native MpARF levels, we found a surprising and profound degradation process that changes the ARF landscape during the first hours of gemma germination. Permissive auxin conditions at the early stage are countered by an ARF landscape that is relatively inhibitory. Over time, this ARF landscape shifts to a relatively activating state. The prediction would be that as time progresses, gemmae will become more sensitive to small changes in auxin concentrations, but this prediction remains to be tested.

We observed an elevated auxin response after dormancy release (Figure 6). By contrast, the quantified endogenous auxin levels were lower after 6 h of growth (Figure 2D). To explain this contradiction, we must consider the genes that are regulated exclusively by MpARF1 or MpARF2. MpARF1 is an auxin-dependent activator, whereas MpARF2 is an auxin-independent transcriptional repressor. Genes activated by MpARF1 alone are therefore expected to be downregulated when auxin levels are low after germination. Genes repressed by MpARF2 are not expected to change in expression with changes in auxin level, but as the MpARF2 protein is degraded, these genes are expected to be de-repressed. Genes regulated by both MpARF1 and MpARF2 are upregulated as their relative ratio increases, favoring MpARF1 function. In addition, we also observed increased expression of MpTIR1 upon dormancy release, suggesting modulation of the auxin response system at multiple points. Therefore, the transcriptional output is a reflection of not only the auxin level itself but also the relative concentrations of the signaling proteins and their stability at the stage of growth that caused this contradictory response.

We could connect MpARF degradation to an active control of A-/B-ARF stoichiometry. Even in the absence of any treatment, and in static observations, gemmae represent a rich landscape of MpARF1:MpARF2 stoichiometries. From first principles, and supported by mathematical modeling, these sites of varying stoichiometries should translate into areas with different auxin response outputs. Indeed, we see that the cells with the most “activating” stoichiometry are rhizoid initial cells, which are known to be highly sensitive to externally applied auxin (Ashton et al., 1979; Sakakibara et al., 2003; Prigge et al., 2010). Thus, the endogenous MpARF accumulation patterns will likely translate to a corresponding map of auxin sensitivities. Unfortunately, these are hard to map at present owing to the absence of a cellular-resolution reporter for mapping of the auxin response output. Given that stoichiometry manipulation does prevent normal auxin response and development, we do expect that the maps, both in the gemma studied here and beyond, will be an exciting starting point for mapping sites of auxin action.

A key question is how changes in ARF stoichiometry are brought about. In principle, any gene/protein regulatory process can contribute to protein accumulation, and this stoichiometry therefore serves as a central pivot point for the control of auxin output. Although it remains to be seen what transcriptional inputs contribute to diverse MpARF gene expression patterns, we do see that, given unequal starting levels, a relatively generic degradation rate can create large changes in MpARF1:MpARF2 stoichiometry. Identification of components in the degradation mechanism, as well as in (post)transcriptional controls such as the microRNA390–TAS3–ARF pathway (Xia et al., 2017), will help to resolve the tuning mechanisms.

MpARF1 and MpARF2 are the sole representatives of the A-class and B-class ARFs in *Marchantia*, and both are derived from an inferred algal proto-A-/B-ARF that is likely represented by ARF proteins of streptophyte algae (Mutte et al., 2018). It is thus plausible that proteolytic degradation is a property inherited from the proto-A-/B-ARF. This raises the question of how widespread this type of regulation is. Several of the 23 *Arabidopsis* ARFs (AtARF1, AtARF6, AtARF7, AtARF17, and AtARF19) have been reported to be proteasomally degraded (Salmon et al., 2008; Lakehal et al., 2019; Jing et al., 2022). Neither the degron nor the biological relevance of these degradations is entirely clear, but there is clearly potential for this degradation mechanism to be intimately connected to auxin response.

## Methods

### Plant growth conditions

*M. polymorpha* male Takaragaike-1 (Tak-1) and female Takaragaike-2 (Tak-2) plants were used as the wild-type variety. For vegetative propagation, plants were grown on ½ Gamborg B5 media plates in growth chambers with 40 μmol photons m^−2^ s^−1^ continuous white light at 22°C. For sexual reproduction, plants were grown on ½ Gamborg B5 medium supplemented with 1% sucrose and maintained under 40 μmol photons m^−2^ s^−1^ continuous white fluorescent light for 1 month. Plants were then moved into 40 μmol photons m^−2^ s^−1^ continuous white light supplemented with 15 μmol photons m^−2^ s^−1^ far-red light to induce antheridiophore and archegoniophore development. Plants were repeatedly crossed manually to ensure fertilization. Sporangia with mature spores were collected aseptically and used in spore transformation.

### Development of genomic knockin translational fusions

*Marchantia* knockin lines were developed to study native auxin response proteins at endogenous concentrations. A nopaline synthase (NOS) terminator and a fluorescent marker gene (either mScarlet-I or mNeonGreen) were cloned sequentially at the HindIII restriction site of the pJHY-TMp1 binary plasmid to create pJHY-mScarlet-I and pJHY-mNeonGreen vectors. After each cloning step, the HindIII site was regenerated by adding a HindIII site in the forward primer. This enabled subsequent cloning at the 5′ end of the previous insert in the same plasmid. Two 3.5-kb genomic DNA fragments were amplified by PCR and used as homologous arms for recombination. The first genomic DNA fragment contained the 3.5-kb sequence upstream of the stop codon of the gene of interest, and the second fragment was composed of the 3.5-kb sequence downstream of the stop codon. The first fragment was cloned at the HindIII site, and the second fragment was cloned at the AscI site of pJHY-mScarlet-I and pJHY-mNeonGreen. This cloning strategy was used to create homologous recombination constructs for ARF1, ARF2, ARF3, TIR1, Aux/IAA, and ncARF. Wild-type (Tak-1) spores were transformed by the *Agrobacterium-*mediated transformation protocol described in Ishizaki et al. (2008). Transformants were selected on ½ Gamborg B5 + 100 mg/L cefotaxime medium with 10 mg/L hygromycin selection. Genomic DNA PCR was used to isolate true knockin lines.

### Generation of *MpARF2* overexpression lines

To overexpress *MpARF2* in *Marchantia*, the *MpARF2* coding sequence was amplified and cloned under the *MpEF1* promoter. The *MpARF2*-CDS was cloned into the published gateway vectors pMpGWB103 and pMpGWB108 (Ishizaki et al., 2015) to generate an unfused and a citrine-fluorophore-fused version, respectively. Constructs were transformed in *Marchantia* Tak-1 using *Agrobacterium*-mediated transformation of gemmae. Positive transformants were obtained through hygromycin selection on ½ Gamborg B5 medium plates.

### Auxin sensitivity and physiological analysis of knockins

Knockin lines were tested for their wild-type-like growth, physiology, and auxin sensitivity. Tak-1, Tak-2, and all knockin lines were treated with ½ B5 medium supplemented with either DMSO or 3 μM 1-NAA and grown for 7 days. On the 8th day, plants (*n* = 10 per genotype) were imaged with a stereomicroscope to compare their physiological responses to auxin (Supplemental Figure S1).

### Microscope slide-mount setup for time-course imaging

A microscope slide mount was set up for live imaging of gemmae to precisely track a selected set of cells for temporal protein expression analysis. The mount consisted of a circular aluminum disc with a plastic inset fitted at the center of the disc (Supplemental Figure S11). Melted ½ B5 medium with or without desired treatments was poured into the cavity of the plastic inset and allowed to solidify. Gemmae were carefully placed on top of the solidified medium and covered with a round coverslip. The bottom of the mount was sealed with parafilm to prevent any evaporation and drying of the medium during time-series imaging. Between two imaging time points in a time-series experiment, the mounts were placed inverted in growth chambers to keep the gemmae exposed to light and allow for normal growth.

### Confocal live-cell imaging

All live-cell imaging was performed on a Leica SP8X-SMD confocal microscope equipped with hybrid detectors and a pulsed (40 MHz) white-light laser. mNeonGeen and mScarlet-I were excited with the 506-nm  and 561-nm laser lines, respectively. The laser powers were set at 4% output to avoid bleaching of the fluorophores. Fluorescence was detected between 512 and 560 nm (mNeonGreen) and 570 and 620 nm (mScarlet-I) using hybrid detectors in photon counting mode. Z-stack images were acquired using a 20× water immersion objective lens and time-gated detection to suppress chlorophyll autofluorescence. Images were processed using ImageJ software. Maximum-intensity projections of z-stack images were used to quantify total cellular fluorescence in each analyzed nucleus, corrected for background fluorescence. For presentation of the representative images of protein expression patterns, outlines of gemmae were cut in Adobe Photoshop and placed onto a black background. For quantification of images, the background signal was always taken into consideration.

### RNA extraction, cDNA synthesis, and qPCR

Total plant RNA was extracted from gemmae collected from gemma cups of 4-week-old Tak-1 and knockin plants and subsequently incubated in liquid ½ B5 medium for 0, 3.5, and 8 h before freezing in liquid nitrogen. RNA was extracted from ground tissue using the TRIzol reagent and the Qiagen Plant mini-kit. An on-column RNase-free DNase (Qiagen) treatment was performed before final elution. cDNA was synthesized from 1 μg total RNA using the iScript Reverse Transcriptase kit (Bio-Rad). qPCR reactions were carried out using 2× IQ SYBR green (Bio-Rad) on a CFX384 Touch Real-Time PCR detection system (Bio-Rad). Data analysis was performed as described in Taylor et al. (2019). The housekeeping genes *MpEF1a* and *MpSAND* were used for transcript-level normalization.

### RNA-seq data analysis

The quality of the raw fastq reads was analyzed in FastQC. Reads were then mapped onto the *M. polymorpha* genome (MpTak1v5.1, accessed through MarpolBase, https://marchantia.info/download/tak1v5.1/) using HISAT2 version 2.2.1 (Kim et al., 2019) with -dta and -trim5 10 as additional parameters. SAM and BAM files were handled using SAMtools version 1.11 (Danecek et al., 2021). The raw reads were then counted using featureCounts version 2.0.1 (Liao et al., 2014) with the additional parameters: -t exon; -g gene_id; -Q 30; -p; -primary. The results were imported into R version 3.6.1, and raw count normalization as well as identification of differentially expressed genes (adjusted *p value [*padj] <0.05) were performed using DESeq2 (Love et al., 2014). Genes with a total read count <45 were excluded from the analysis, and genes with an absolute log_2_(FC) of >1 and padj < 0.05 were deemed differentially expressed. All plots were generated in R (www.r-project.org). Raw RNA-seq reads were deposited in the NCBI Sequence Read Archive under project number PRJNA1019931.

### Inducible ARF2 overexpression

For inducible MpARF2 overexpression, the pEF1MpARF2-GR lines were used (Kato et al., 2020). Plants (*n* = 10 per genotype) were treated with either DMSO or 1 μM dexamethasone in B5 medium and imaged after 3 days to look for rhizoid formation as an indicator of gemma germination. Dexamethasone treatment was used to induce the movement of ARF2-GR from the cytosol to the nucleus.

### Mathematical modeling

Details of modeling are described in detail in Supplemental File 1.

### IAA quantification

Total IAA was quantified to estimate the total cellular auxin levels during gemma growth. Gemmae were collected from 4-week-old Tak-1 plants. Tak-1 gemmae were grown on liquid ½ B5 medium, and samples were collected after 0, 3.5, 8, and 24 h of growth. Samples were snap frozen in liquid nitrogen, ground into a fine powder, and weighed. For extraction of IAA, ∼150 mg snap-frozen plant material was used per sample. Tissue was ground into a fine powder at −80°C using 3-mm stainless-steel beads at 50 Hz for 2 × 30 s in a TissueLyser LT (Qiagen). Ground samples were extracted with 1 mL cold methanol containing [phenyl ^13^C_6_]-IAA (0.1 nmol/mL) as an internal standard in a 2-mL Eppendorf tube as described previously (Ruyter-Spira et al., 2011). Samples were filtered through a 0.45-μm Minisart SRP4 filter (Sartorius) and measured on the same day. IAA was measured on a Waters Xevo TQ-S tandem quadrupole mass spectrometer.

## Funding

This work was supported by the 10.13039/501100003246Netherlands Organisation for Scientific Research, the Netherlands (grants ALWOP.402 and OCENW.M20.031 to J.W.B.) and the Human Frontiers Research Program (grant RGP0015/2022 to D.W.).

## Author contributions

Conceptualization, D.W. and J.W.B. Investigation, S.D., M.d.R., S.M., M.D.A., and W.K. Modeling, E.F. and S.B. Writing – original draft, S.D., M.d.R., W.K., S.B., E.F., J.W.B., and D.W. Writing – review & editing, S.D., J.W.B., and D.W. Supervision, J.W.B. and D.W.

## Supplemental information

Document S1. Supplemental Figures 1–15 and Supplemental File 1

Document S2. Article plus supplemental information
