# Supplementary material for: Quantitative imaging reveals the role of MpARF proteasomal degradation during gemma germination
Source: Plant Commun. 2024 Jul 9;5(11):101039. doi: 10.1016/j.xplc.2024.101039 (PMC11589480; doi:10.1016/j.xplc.2024.101039)
Supplement: Document S1. Supplemental Figures 1–15 and Supplemental File 1 [file mmc1.pdf]

Plant Communications, Volume *5*

Supplemental information

Quantitative imaging reveals the role of MpARF proteasomal degradation during gemma germination

Shubhajit Das, Martijn de Roij, Simon Bellows, Melissa Dipp Alvarez, Sumanth Mutte, Wouter Kohlen, Etienne Farcot, Dolf Weijers, and Jan Willem Borst

Supplemental information

Quantitative imaging reveals the role of MpARF proteasomal degradation during gemma germination

Shubhajit Das[1,4], Martijn de Roij[1], Simon Bellows[2], Melissa Dipp Alvarez[1], Sumanth Mutte[1], Wouter Kohlen[3], Etienne Farcot[2], Dolf Weijers[1*], Jan Willem Borst[1*]

[1]Laboratory of Biochemistry, Wageningen University and Research, the Netherlands.

[2]School of Mathematical Sciences, University of Nottingham, United Kingdom.

[3]Laboratory of Molecular Biology, Wageningen University and Research, the Netherlands.

[4]Current address: Institute of Science and Technology, Austria

*Corresponding authors dolf.weijers@wur.nl and janwillem.borst@wur.nl

Supplemental Figures

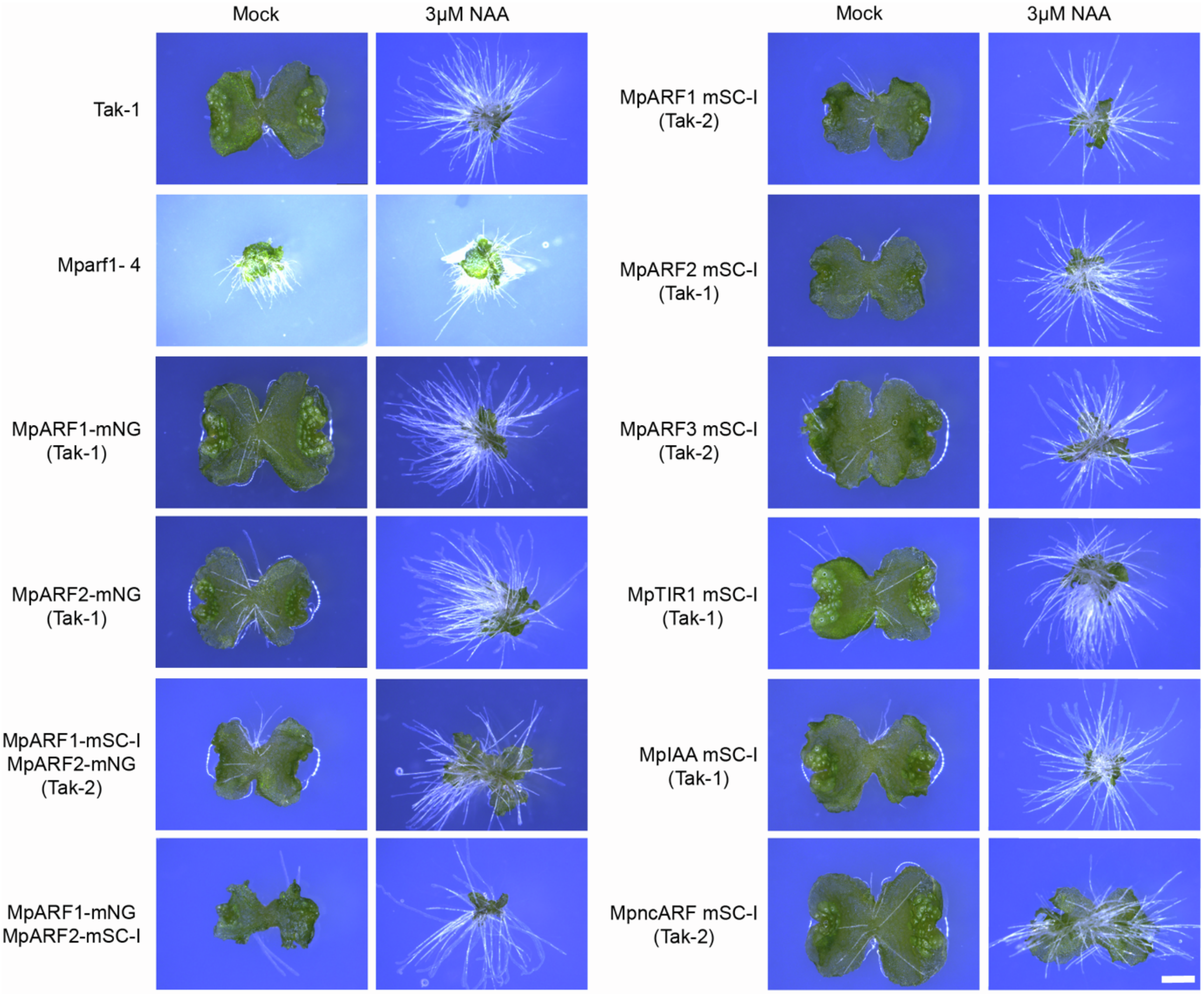

Supplemental Figure S1: Phenotype and auxin response in knock-in lines.
Auxin response assay on all genomic knock-in lines. All knock-ins were treated with mock or 3µM NAA and imaged after 1 week. All knock-ins show wild-type-like auxin response with thallus growth inhibition and ectopic rhizoid formation. Scale bar = 1 mm.

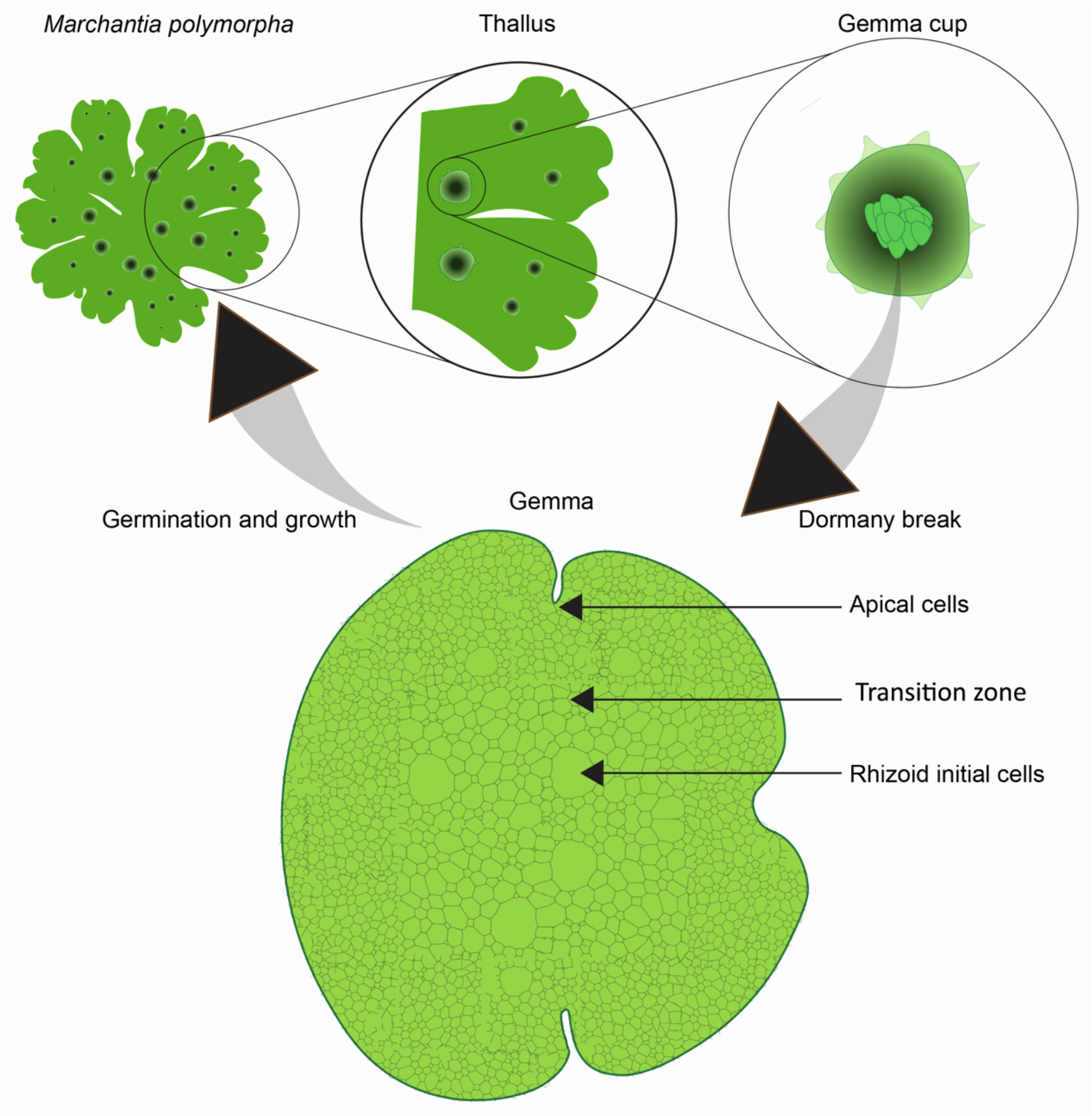

Supplemental Figure S2. Schematic representation of the Marchantia tissues and different cell types within a gemma.

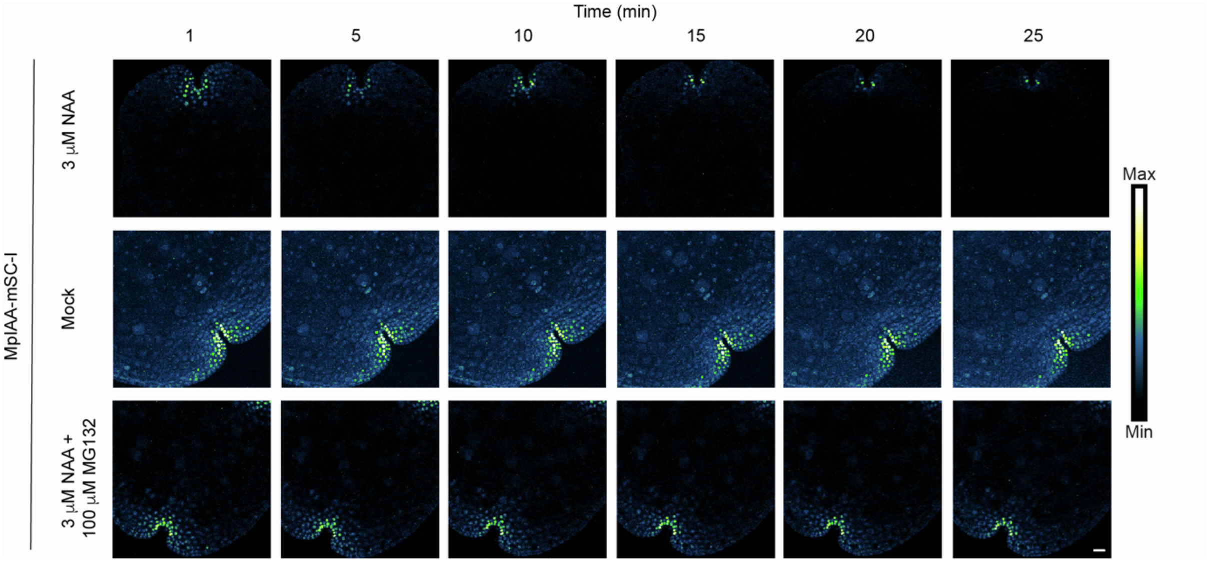

Supplemental Figure S3: Time-lapse imaging on MpIAA-mSC-I gemmae pre-treated with (50 μM)L-Kynurenine and (50 μM) Yucasin. Upon auxin (1-NAA; 3μM) treatment, fluorescence rapidly decreases due to MpIAA-mSC-I degradation, whereas the fluorescence remains constant if the sample is mock treated or incubated with the proteasomal inhibitor MG132. Scale bar = 25 μm.

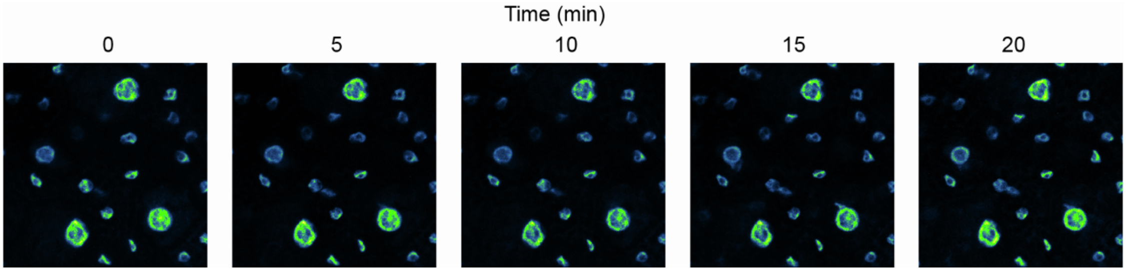

Supplemental Figure S4; time course of MpARF1-mSC-I at same time of MpIAA experiment showing no significant photobleaching of MpARF1-mSC-I.

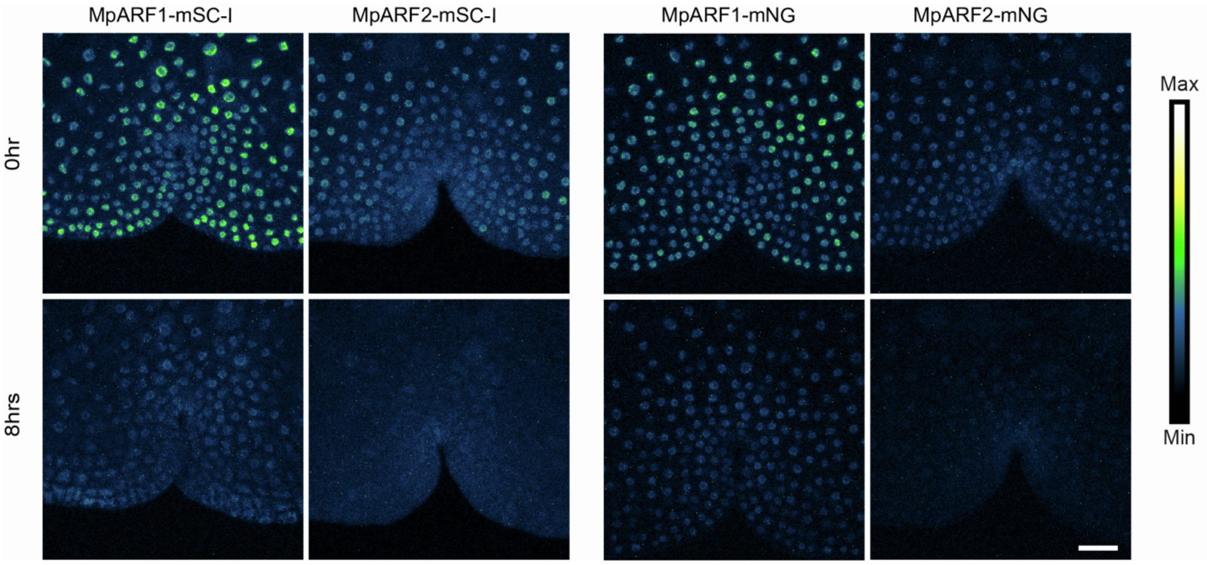

Supplemental Figure S5. MpARF1 and MpARF2 protein accumulation dynamics is independent of the fluorophore tag used.

Time course imaging on MpARF1 and MpARF2 single knock-in lines fused to mScarlet-I (mSC-I) and mNeonGreen (mNG) fluorescent proteins. Both mNG and mSC-I fusion variants of MpARF1 and MpARF2 show similar fluorescence decline during gemma germination. Scale bar = 50 µm.

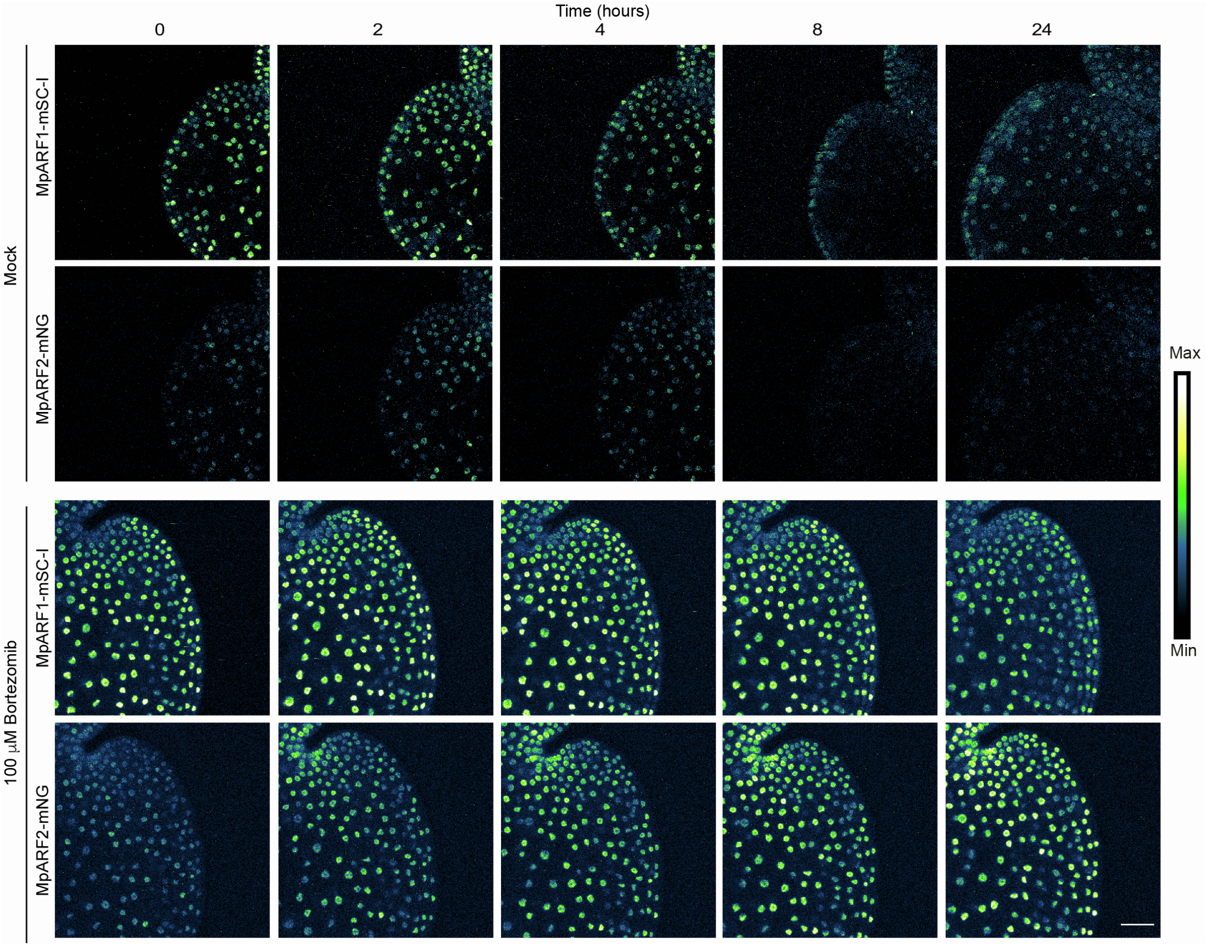

Supplemental Figure S6. Bortezomib prevents MpARF degradation.
Treatment with proteasomal degradation inhibitor Bortezomib also blocks the degradation of MpARF1-mSC-I and MpARF2-mNG. Scale bar = 50 μm

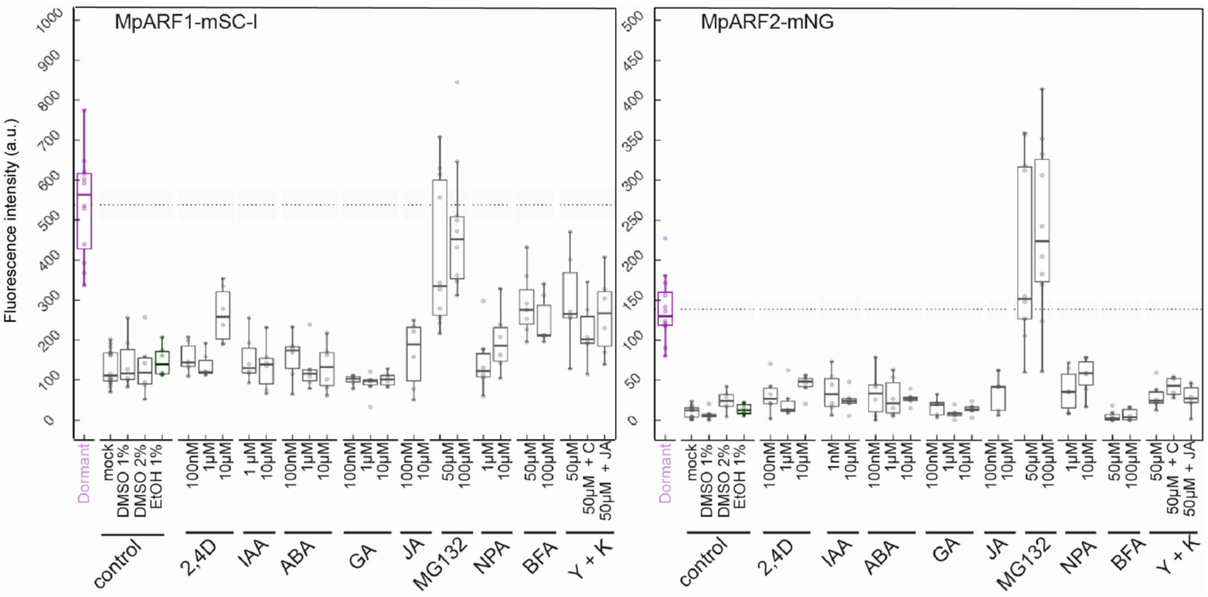

Supplemental Figure S7. Proteasomal degradation of MpARF is independent of auxin and other tested common plant hormones.

Treatment with common plant hormones such as natural (IAA) and synthetic (2,4-D) auxin, abscisic acid (ABA), gibberellic acid (GA), and jasmonic acid (JA), as well as inhibitors of auxin biosynthesis (Yucasin and Kyneurenine), auxin transport (NPA) and endocytosis(BFA), do not have any effect on MpARF degradation. Only proteasomal inhibitor (MG132) can block MpARF degradation.

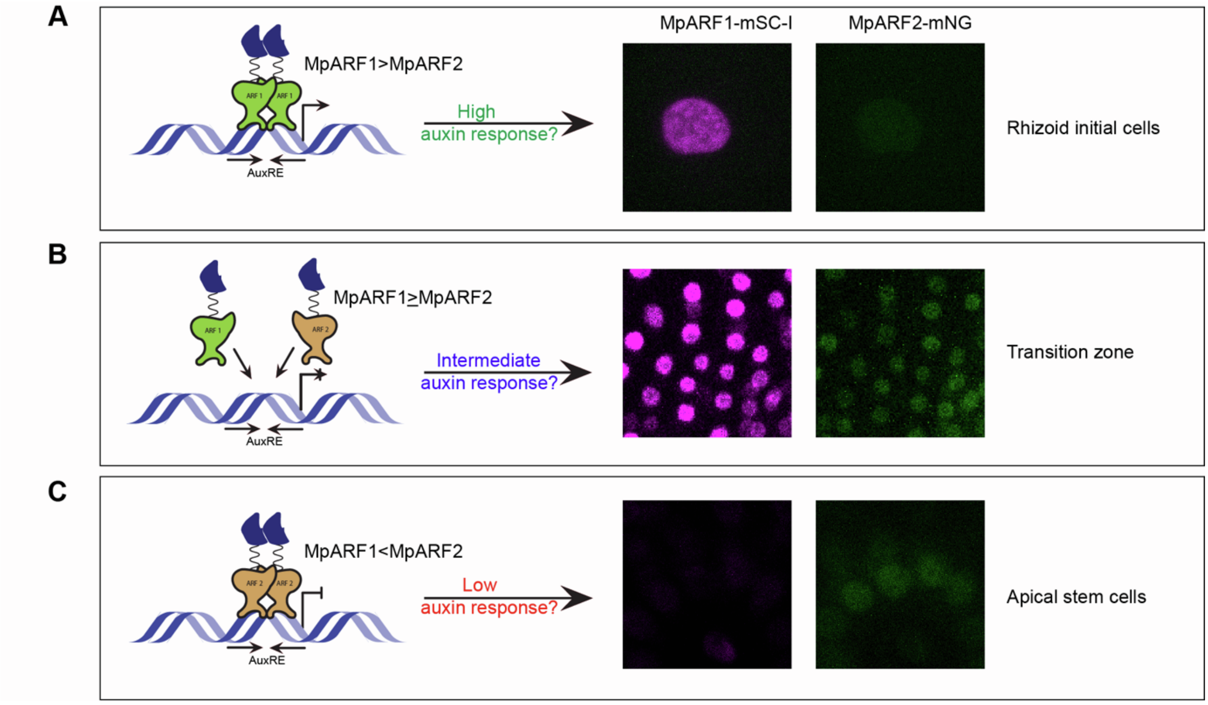

Supplemental Figure S8. Model for how differential MpARF stoichiometry may underpin auxin response output across tissues.

Three possible modes of gene regulation by the relative stoichiometry of class A and class B ARFs, adapted from Kato et. al. 2020. **A)** At a relatively higher concentration of MpARF1, DNA binding and gene activation is controlled by MpARF1 in an auxin-dependent manner. This mode might be operational in rhizoid initial cells where MpARF2 expression is below detection limit and MpARF1 is abundant. **B)** The second mode of gene regulation considers the presence of both class A and B MpARFs leading to competition-driven gene regulation(in the transition zone. **C)** The third mode describes gene repression by class B MpARFs, independent of cellular auxin concentrations. Apical meristem cells of gemmae could represent this third mode as these cells have relatively higher MpARF2 expression and no MpARF1 expression.

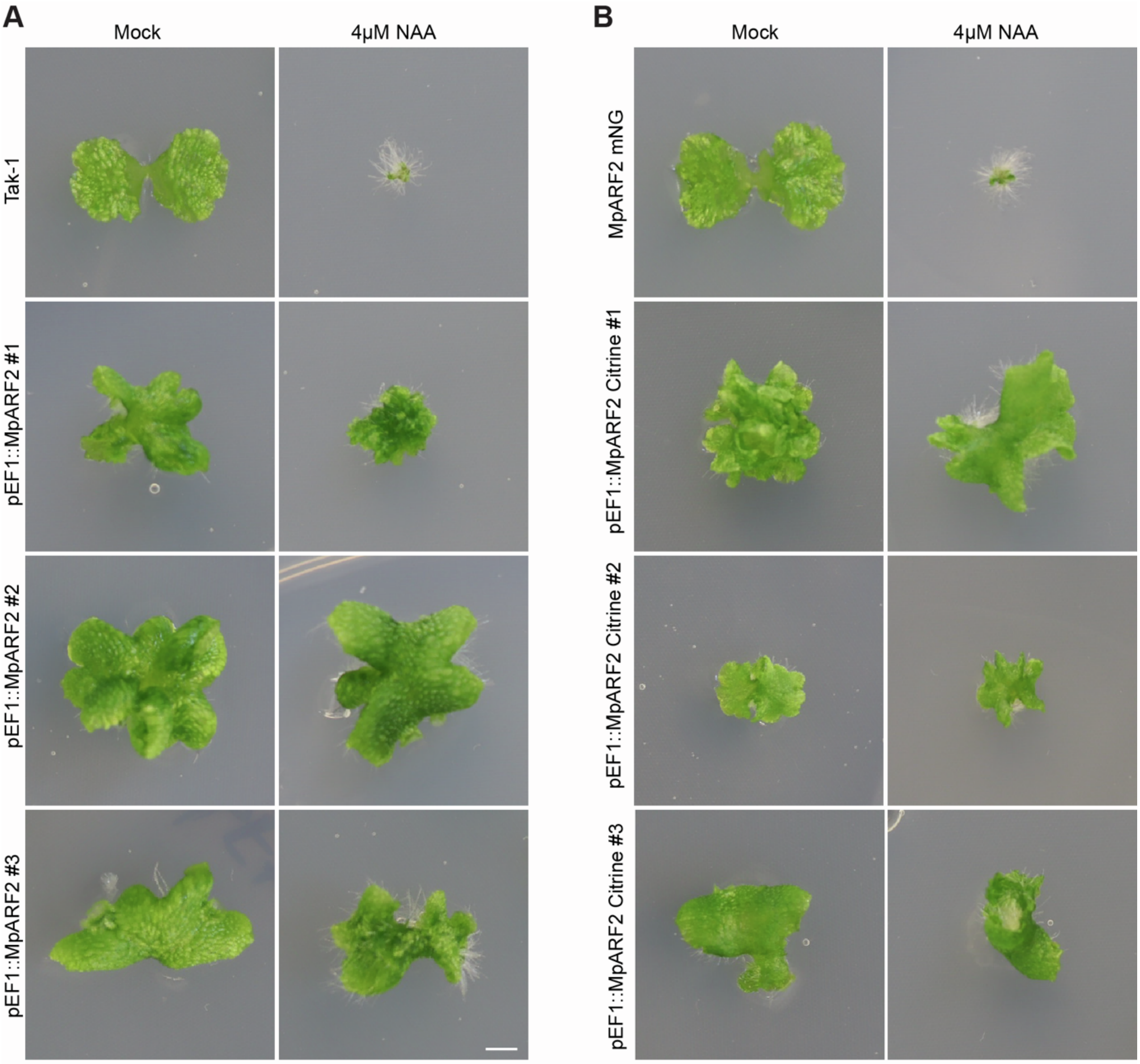

Supplemental Figure S9. Accumulation of MpARF2 leads to an auxin insensitive phenotype.

A,B) Growth of three independent pEF1::MpARF2 (**A**) and pEF1::MpARF2-Citrine (**B**) lines and Tak-1 control on control medium or medium containing 4 µM NAA. **C)** Expression of MpARF2-Citrine in pEF1::MpARF2-Citrine line#1. Scale bar = 25 µm.

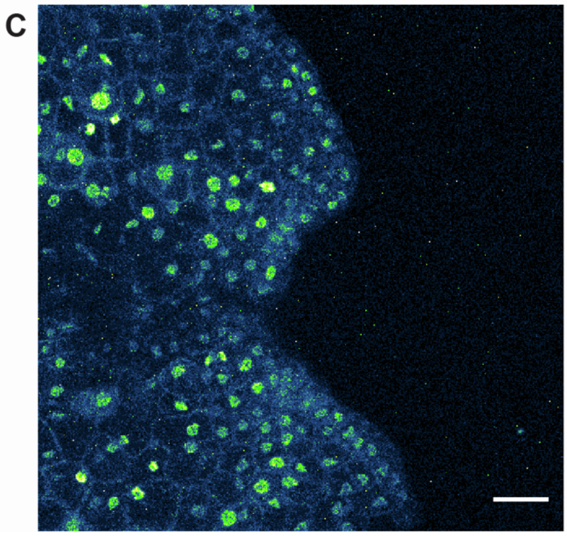

.

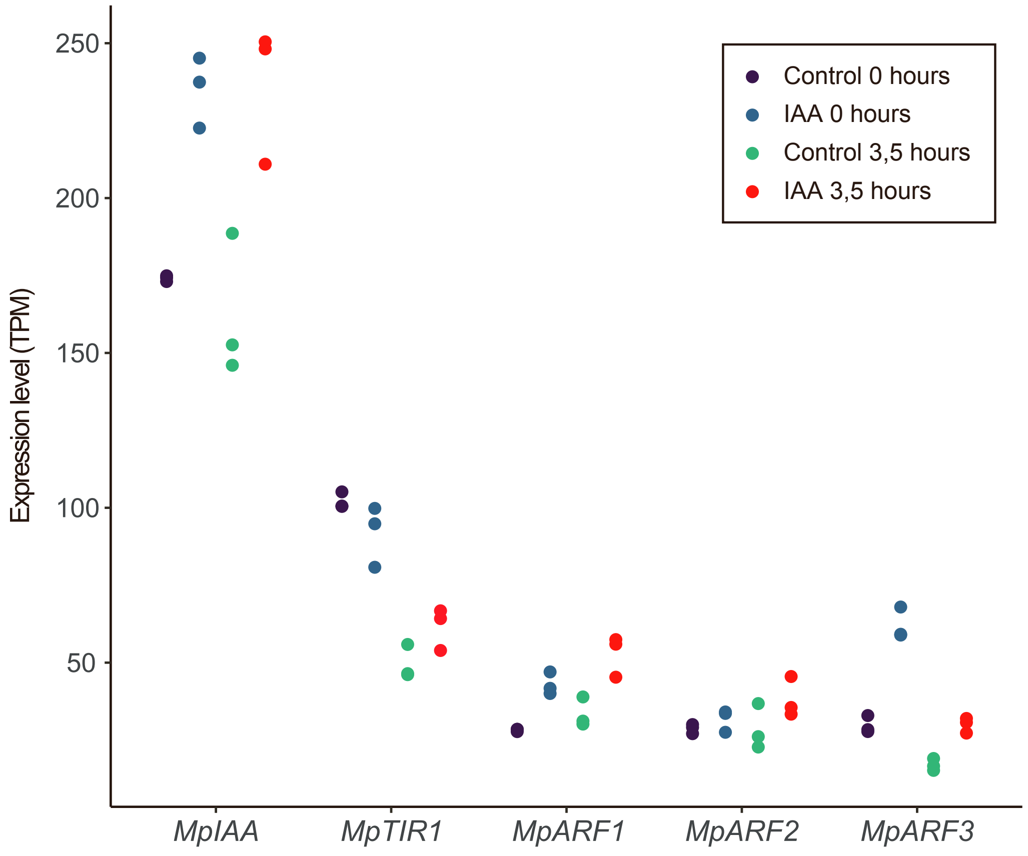

Supplemental Figure S10: Raw TPM expression values for MpIAA, MpTIR1 and MpARF1/2/3 in control- and IAA-treated gemmae at 0 and 3,5 hours after removal from the gemmae cup. Values of each replicate are shown.

:

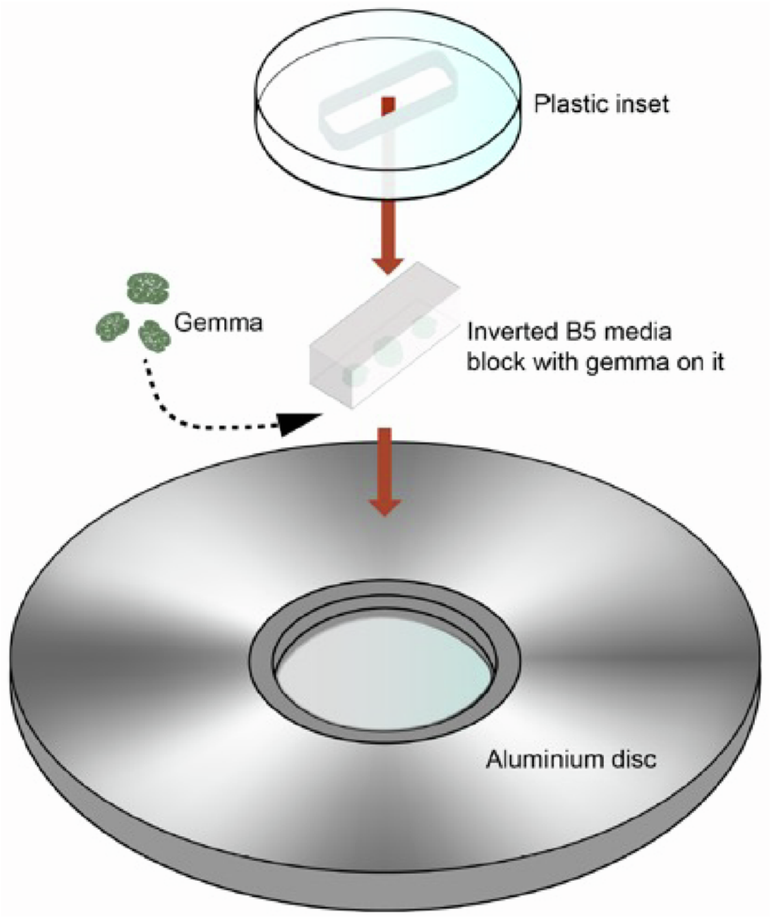

Supplemental Figure S11: Microscope sample mount used to track protein accumulation patterns gemmae.
Basic design of the microscope slide mount, used for time course imaging. B5 media blocks are solidified inside the plastic inset and gemmae are placed on top of the media. The media is supplemented with desired treatment or mock before casting in the inset. A round coverslip is gently placed on top of the gemmae. The plastic inset containing the gemma samples on the media block, is inverted and placed on the aluminium disc and tightened with a screw to prevent movement. Evaporation of water from the media block is prevented by sealing the reverse side of the block with parafilm.

Supplemental File 1

Mathematical model
In this supplement, a full description of the auxin signaling model for *Marchantia polymorpha* (Mp), used in the main paper, is provided. Note that for simplicity, the prefix Mp is typically dropped in the following as the model is largely species independent and relies on the general structure of the nuclear auxin pathway (NAP), with a few ad hoc assumptions. Firstly, the model is described, then some theoretical properties are discussed before showing some numerical simulations.

Model description
The main underlying assumptions are similar to previous NAP models from literature (Farcot et al., 2015), with adaptations motivated by the Marchantia data presented in the main paper. It is known that oligomers involving both ARF and Aux/IAA proteins can form via both a protein-protein domain, DIII-IV (or PB1 since its structure was uncovered) (Nanao et al., 2014) and a DNA binding domain, DBD (Boer et al., 2014). Based on this, the model includes populations of proteins and a generic AuxRE-bearing promoter, describing the competition between the different complexes that can be formed from these actors. As the model focuses on the early stages of development, at which Aux/IAA proteins are essentially absent from the system, we do not include Aux/IAA in the models. On the other hand, we consider both ARF1 and ARF2 along with all the complexes they can form, though limiting oligomer sizes to 2. This leads to the possible configurations - and transitions between them – shown in Supplemental Figure S12.

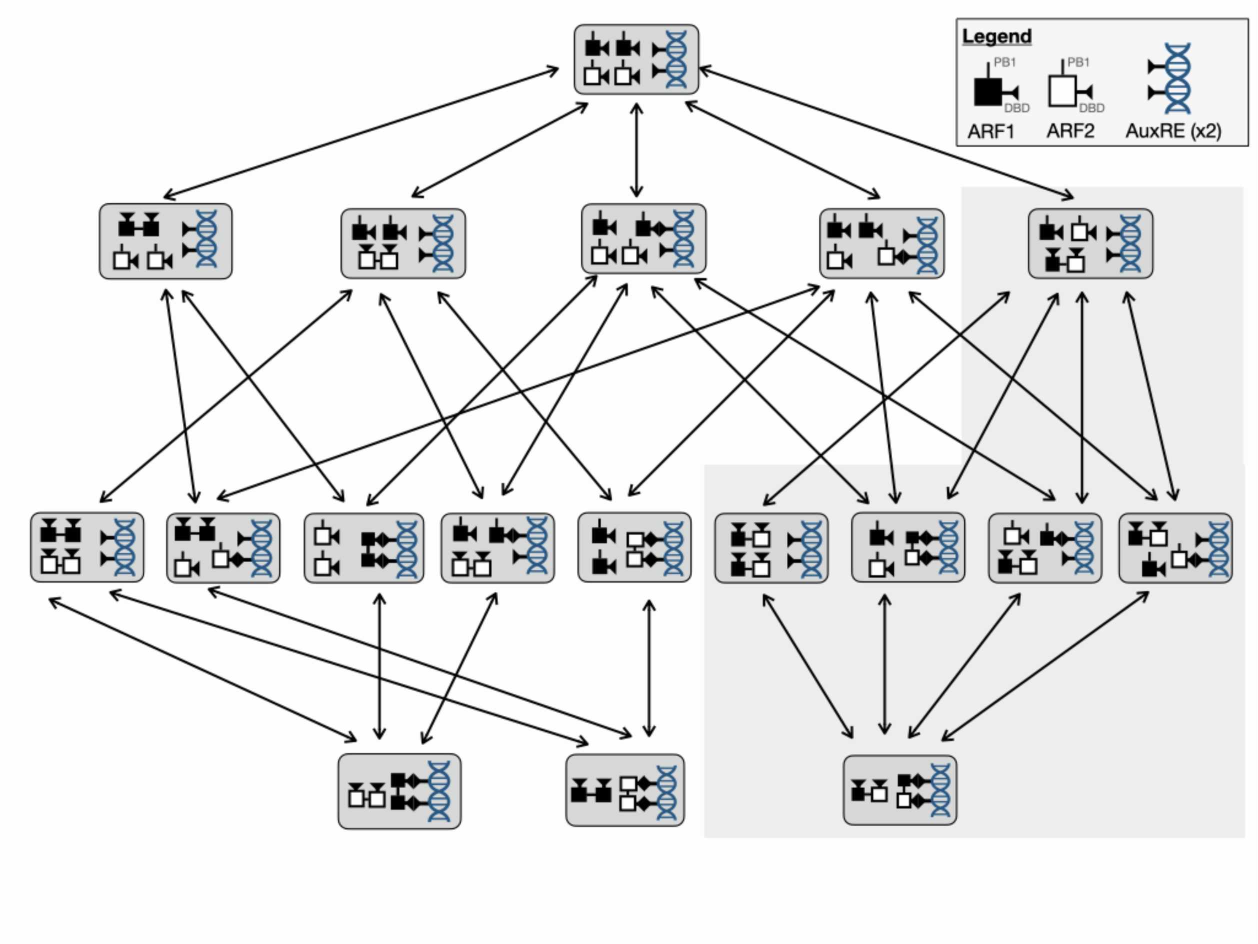

Supplemental Figure S12: All possible protein/AuxRE complexes included in the model. As one possible scenario, we have ignored all MpARF heterodimers, indicated by the grey box.

The equations are in an exact correspondence (using mass action) with the diagram in Supplemental Figure S12

$$\frac{dA_1}{dt} = -2\alpha_{A_1A_1}A_1A_1 + 2\theta_{D_{A_1A_1}}D_{A_1A_1} - \alpha_{A_1A_2}A_1A_2 + \theta_{A_1A_2}D_{A_1A_2} - \alpha_{GA_1}GA_1 + \theta_{GA_1}G_{A_1}$$
$$-\alpha_{A_1GA_1}A_1G_{A_1}\theta_{A_1GA_1}G_{A_1A_1} - \alpha_{A_1GA_2}A_1G_{A_2} + \theta_{A_1GA_2}G_{A_1A_2}$$

$$\frac{dA_2}{dt} = -\alpha_{A_1A_2}A_1A_2 + \theta_{A_1A_2}D_{A_1A_2} - 2\alpha_{A_2A_2}A_2A_2 + 2\theta_{A_2A_2}D_{A_2A_2} - \alpha_{A_2GA_1}A_2G_{A_1} + \theta_{A_2GA_1}G_{A_1A_2}$$
$$-\alpha_{GA_2}GA_2 + \theta_{GA_2}G_{A_2} - \alpha_{A_2GA_2}G_{A_2}A_2 + \theta_{A_2GA_2}G_{A_2A_2}$$

$$\frac{dD_{A_1A_1}}{dt} = \alpha_{A_1A_1}A_1A_1 - \theta_{D_{A_1A_1}}D_{A_1A_1} - \alpha_{A_1A_1G}D_{A_1A_1}G + \theta_{A_1A_1G}G_{A_1A_1}$$

$$\frac{dD_{A_1A_2}}{dt} = \alpha_{A_1A_2}A_1A_2 - \theta_{A_1A_2}D_{A_1A_2} - \alpha_{A_1A_2G}D_{A_1A_2}G + \theta_{A_1A_2G}G_{A_1A_2}$$

$$\frac{dD_{A_2A_2}}{dt} = \alpha_{A_2A_2}A_2A_2 - \theta_{A_2A_2}D_{A_2A_2} + \theta_{A_2A_2G}G_{A_2A_2} - \alpha_{A_2A_2G}D_{A_2A_2}G$$

$$\frac{dG}{dt} = -\alpha_{GA_1}GA_1 + \theta_{GA_1}G_{A_1} - \alpha_{GA_2}GA_2 + \theta_{GA_2}G_{A_2} - \alpha_{A_1A_1G}D_{A_1A_1}G + \theta_{A_1A_1G}G_{A_1A_1}$$
$$-\alpha_{A_1A_2G}D_{A_1A_2}G + \theta_{A_1A_2G}G_{A_1A_2} - \alpha_{A_2A_2G}D_{A_2A_2}G + \theta_{A_2A_2G}G_{A_2A_2}$$

$$\frac{dG_{A_1}}{dt} = \alpha_{GA_1}GA_1 - \theta_{GA_1}G_{A_1} - \alpha_{A_1GA_1}A_1G_{A_1} + \theta_{A_1GA_1}G_{A_1A_1} - \alpha_{A_2GA_1}A_2G_{A_1} + \theta_{A_2GA_1}G_{A_1A_2}$$

$$\frac{dG_{A_2}}{dt} = \alpha_{GA_2}GA_2 - \theta_{GA_2}G_{A_2} - \alpha_{A_1GA_2}A_1G_{A_2} + \theta_{A_1GA_2}G_{A_1A_2} - \alpha_{A_2GA_2}G_{A_2}A_2 + \theta_{A_2GA_2}G_{A_2A_2}$$

$$\frac{dG_{A_1A_1}}{dt} = \alpha_{A_1GA_1}A_1G_{A_1} - \theta_{A_1GA_1}G_{A_1A_1} + \alpha_{A_1A_1G}D_{A_1A_1}G - \theta_{A_1A_1G}G_{A_1A_1}$$

$$\frac{dG_{A_1A_2}}{dt} = \alpha_{A_2GA_1}A_2G_{A_1} - \theta_{A_2GA_1}G_{A_1A_2} + \alpha_{A_1GA_2}A_1G_{A_2} - \theta_{A_1GA_2}G_{A_1A_2} + \alpha_{A_1A_2G}D_{A_1A_2}G - \theta_{A_1A_2G}G_{A_1A_2}$$

$$\frac{dG_{A_2A_2}}{dt} = \alpha_{A_2A_2G}D_{A_2A_2}G - \theta_{A_2A_2G}G_{A_2A_2} + \alpha_{A_2GA_2}G_{A_2}A_2 - \theta_{A_2GA_2}G_{A_2A_2}$$

The notational conventions are as follows:

- A_1 (resp. A_2) denotes the concentration of MpARF1 (resp. MpARF2).
- D_{XY}, $X, Y \in \{A_1, A_2\}$ denotes the concentration of an X:Y dimer, with parameters α, θ for association/dissociation rates as mentioned, and subscripts being hopefully explicit.
- G , G_{A_1}, G_{A_2}, $G_{A_1A_1}$, $G_{A_1A_2}$, $G_{A_2A_2}$ denote the proportion, or probabilities, that a generic promoter is free, or bound with one of the possible ARF complexes as denoted in subscript.

To assign values to the constants α_{XY} and θ_{XY}, we use estimates based on literature, in particular reference (Fontana et al., 2023). This paper shows that DBD allows for cooperative binding: dimers are more stably bound to DNA than monomers. To account for this, we use a cooperativity parameter γ, typically ≈100. In fact, data from (Fontana et al., 2023) indicates three orders of magnitude, for equilibrium dissociation constants:

$$K_{AA}^d \approx K_{AGA}^d \ll K_{AAG}^d \ll K_{AG}^d,$$

denoting $K^d = \frac{\theta}{\alpha}$ and each occurrence of A being either ARF1 or ARF2. It also appears that the intermediary constant is comparable to the dissociation rate of ARF:ARF dimers.
For simplicity, we rely on 4 pairs of association/dissociation constants:

- Kon1/Koff1 define ARF1 homodimerization; their default values are Kon1=1, Koff1=1.
- Kon2/Koff2 define ARF2 homodimerization; their default values are Kon2=1, Koff2=1.
- Kon3/Koff3 define ARF1/ARF2 heterodimerization, with defaults Kon3=1, Koff3=1.
- Kon4/Koff4 define ARF/AuxRE binding, supposed to be non-specific amongst ARFs; this defines the smallest order of magnitude above. Accordingly, their default values are Kon4=1, Koff4= 1/γ =0.01.

All parameters are listed below, following the conventions discussed above:

Parameter	Value	Description
γ	100	Cooperativity coefficient
$\alpha_{A_1A_1}$ / $\theta_{A_1A_1}$	Kon1 / Koff1	Association / dissociation of ARF1 with itself
$\alpha_{A_2A_2}$ / $\theta_{A_2A_2}$	Kon2 / Koff2	Association / dissociation of ARF2 with itself
$\alpha_{A_1A_2}$ / $\theta_{A_1A_2}$	Kon3 / Koff3	Association / dissociation of ARF1 and ARF2
$\alpha_{A_iG} = \alpha_{A_iA_jG} = 2\alpha_{A_iGA_j}$	Kon4	Association of any ARF to a single AuxRE; A_i, A_j denote any of ARF1/ARF2
$\theta_{A_iG} = \gamma\theta_{A_iA_jG} = \gamma^2\theta_{A_iGA_j}$	Koff4	Dissociation of any ARF to a single AuxRE; A_i, A_j denote any of ARF1/ARF2

Model properties
The structure of the model, see Figure S1, confers some general properties which are true regardless of specific parameters. Firstly, since the roles of the two ARFs are perfectly symmetric (whether heterodimers are included or not), one expects an exact balance between ARF1 (resp. ARF1:ARF1) and ARF2 (resp. ARF2:ARF2), unless there are quantitative differences in their binding/unbinding rates. However, even with similar kinetics the balance between Class A and Class B ARFs can be shifted by changes in the amount of available proteins, due to the presence of three conservation relations:

$$A_1^{tot} \doteq A_1 + 2A_{11} + A_{12} + G_{A_1} + 2G_{A_1A_1} + G_{A_1A_2}$$
$$A_2^{tot} \doteq A_2 + A_{12} + 2A_{22} + G_{A_2} + G_{A_1A_2} + 2G_{A_2A_2}$$
$$G^{tot} \doteq G + G_{A_1} + G_{A_2} + G_{A_1A_1} + G_{A_1A_2} + G_{A_2A_2}$$

Intuitively, these mean that the total amounts of each of ARF1, ARF2 and promoters remain unchanged over time. Therefore, they are completely determined by their initial value at time zero. The model describes how this initial amount is reallocated between the different subpopulations represented in Figure S1. This has two benefits:

- By systematically using initial conditions with $\left(G, G_{A_1}, G_{A_2}, G_{A_1A_1}, G_{A_1A_2}, G_{A_2A_2}\right) = (1,0,0,0,0,0)$, i.e. a population including only unoccupied promoters, the conservation relation guarantees that $G^{tot} = 1$ at all times, i.e. that the model describes a probability distribution.

- Since ARFs are conserved we can represent the experimental time series from the main paper as follows: for each experimental time point we use the experimental amounts of ARF1 and ARF2 as initial condition, from which we derive the distribution of all other variables using the model, which will therefore evolve between each time point. This explains why there is no production or degradation of ARFs included in the model equations. The underlying assumption is that the binding events described in the model are much faster than the 2h period between successive time points. This is justified given the dissociation constants shown e.g. (Han et al., 2014; Fontana et al., 2023), and which are of the order of 1-100 s^{-1}, giving time scales of minutes or shorter.

To describe the 'response' of the system, rather than using arbitrary assumptions to model a transcription rate, we consider the ratio between AuxRE sites occupied by activators and repressors:

$$Response = \frac{G_{A_1} + G_{A_1A_1} + G_{A_1A_2}}{G + G_{A_2} + G_{A_1A_2} + G_{A_2A_2}}$$

Note that whether A_1A_2 dimers are activators or repressors is unclear, so we include them twice. In fact, there is no clear evidence to date that these heterodimers form at all and in the following we will consider both scenarios with and without their formation.
One notices that the response term shown above is entirely auxin independent, which may seem contradictory. This is a consequence of these two facts:

(1) the above concerns a situation where Aux/IAA proteins have been removed.
(2) the primary effect of auxin on the NAP is to induce the degradation of Aux/IAA, hence suppressing their transcriptional repression as well as their sequestration of ARF proteins.

Consequently, in tissues where Aux/IAA have been degraded independently of auxin, it is expected that the effect of auxin itself becomes largely diminished. Including Aux/IAA explicitly in the model would result in a significant increase in complexity, roughly doubling the number of dimers seen in Supplemental Figure S12, and adding many unknown parameters. Given that Aux/IAA is experimentally mostly absent this seems an unnecessary complication.
One may still assess responsiveness to auxin as follows. Even a small amount of Aux/IAA will form dimers with ARF1, which will act as repressors. We denote auxin concentration by x and the population of promoter bound ARF1:Aux/IAA dimers by $G_{A_1I}(x)$. The repressive effect of dimers means that the transcriptional response should include a new term:

$$R(x) = \frac{G_{A_1} + G_{A_1A_1} + G_{A_1A_2}}{G + G_{A_2} + G_{A_1A_2} + G_{A_2A_2} + G_{A_1I}(x)}$$

Since auxin induces Aux/IAA degradation, $G_{A_1I}(x)$ decreases to 0 as x increases. One simple way to measure sensitivity is then to evaluate the rate of response change with respect to auxin:

$$\frac{dR}{dx}(x) = \frac{-(G_{A_1} + G_{A_1A_1} + G_{A_1A_2})}{\left(G + G_{A_2} + G_{A_1A_2} + G_{A_2A_2} + G_{A_1I}(x)\right)^2} \cdot \frac{dG_{A_1I}}{dx}(x)$$

As Aux/IAA is low, it makes sense to consider this rate when $G_{A_1 I}(x) \approx 0$. Also, the term $\frac{dG_{A_1 I}}{dx}(x) < 0$ is indicative of how effective the degradation of Aux/IAA is in response to auxin; with the data available this could only be a speculated figure. Therefore, we have chosen to measure auxin sensitivity as the rate of increase in transcription *relative to* the rate $\frac{dG_{A_1 I}}{dx}(x)$, in the limit of where $G_{A_1 I} \to 0$. In equation:

$$\text{(Auxin Sensitivity)} \qquad S = \frac{G_{A_1} + G_{A_1 A_1} + G_{A_1 A_2}}{\left(G + G_{A_2} + G_{A_1 A_2} + G_{A_2 A_2}\right)^2}$$

Numerical simulations (time series)

Time course simulations are run using the well-known ODE solver *scipy.integrate.odeint* available in Python 3.9, as well as plotting functions from the matplotlib library. All codes are available and free to use.

As mentioned previously, one uses the five timepoints t=0, 2, 4, 6, 8h present in the data. For each, we set an initial condition where ARF1 and ARF2 are directly read from the data, and all other variables are zero except for $G = 1$ (as discussed). We then solve the model using *odeint*, until nearing equilibrium. The obtained values are used for the corresponding timepoint. Successive timepoints are connected linearly, in absence of information regarding intermediary times. Using this method, the default parameter values lead to the time series shown in Figure S2.

As expected, the decrease of ARF monomers over time induces a redistribution of all other variables which results, overall in an increased transcriptional response, relative to a situation where ARF levels are maintained; with the intial ARFs amounts, there is ≈2.5x more activator-bound AuxREs than repressor-bound, and this raises to ≈5x as ARFs get degraded.

Using the model, different conditions can be simulated to assess their effect on the system's behavior. We include a small number here, but the python script could be used to investigate an endless number or alternative conditions. We restrict to two main aspects:

(1) As mentioned, there is no direct evidence that ARF1:ARF2 dimers form, only the fact that they cannot be ruled out based on protein structure only. We therefore ran simulations where Kon3=0, precluding the formation of heterodimers. Note that with our default assumptions on parameters, the routes $A_1 + A_2 + G \to G_{A_1} + A_2 \to G_{A_1 A_2}$ are still permitted since their rates are linked to Kon4. For this scenario specifically, we therefore forced Kon4 to 0, but only in terms leading to the formation $G_{A_1 A_2}$, still allowing every other AuxRE configurations.

(2) Also mentioned earlier, the symmetry of the reaction network could be broken in favor of ARF1 accumulation if the rates Kon1/Koff1 are distinctly more favorable to dimer formation than the rates Kon2/Koff2. We therefore also considered this scenario, to assess how much this would enhance the response increase seen with default parameters (where all 4 rates are equal).

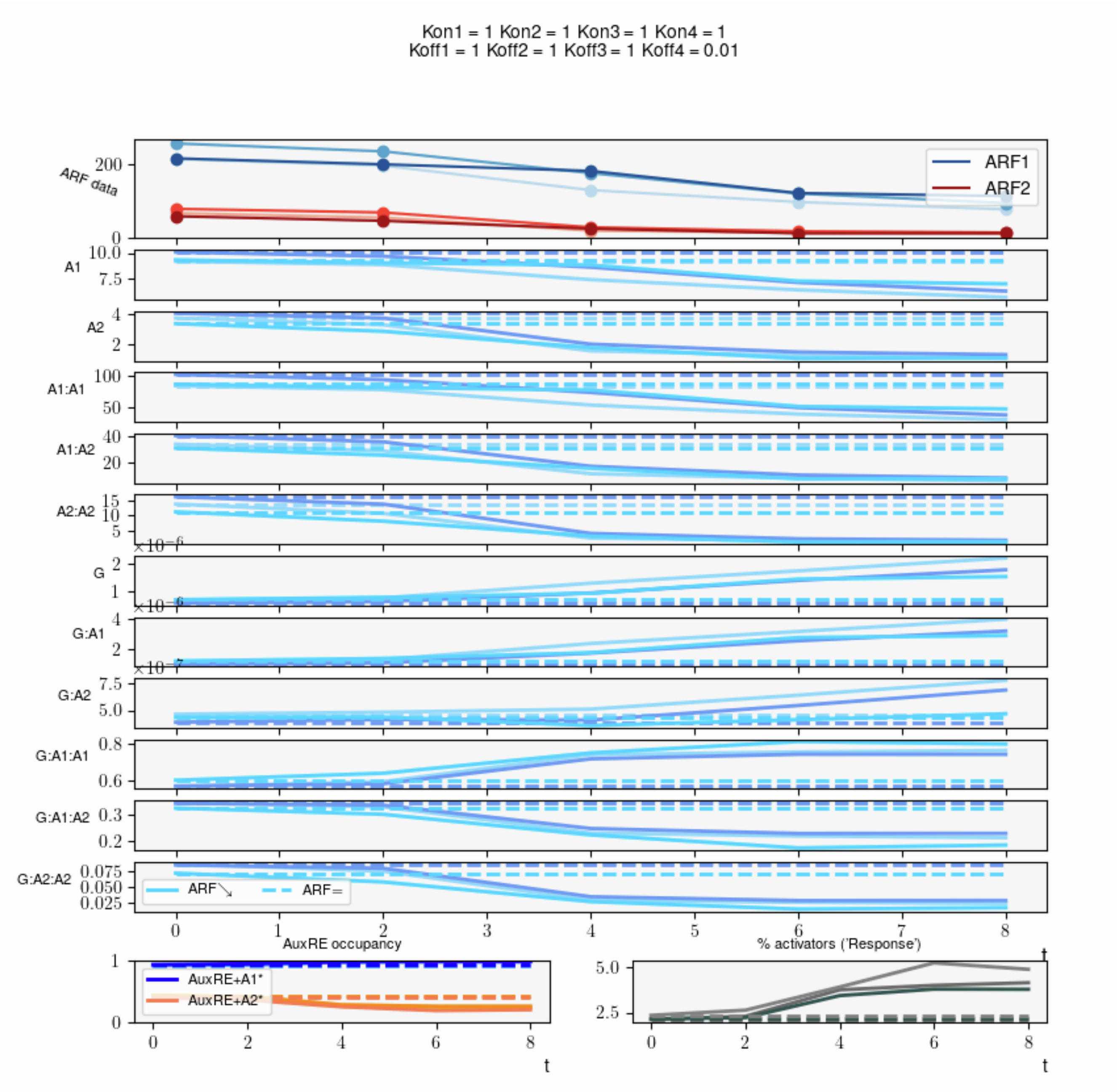

Supplemental Figure S13: Time simulation as described in the text, for the default parameter values. Experimental data is included in the first row. For comparison, variables as they would be without any decrease of ARF over time are shown using dashed lines. Shades of similar colors represent the three replicate time series obtained experimentally.

These alternative conditions are summarized in Supplemental Figure S14. Interestingly, there is little effect on the response resulting from an enhanced ARF1:ARF1 formation. This could be an indication that the repression of ARF seen in the data is a more effective mechanism to generate a transcriptional response. On the other hand, the removal of ARF1:ARF2 heterodimers (highlighted cases in Supplemental Figure S14) systematically leads to an improved response. This is due to the reduced competition between paths of dimer formation. It would be interesting to confirm experimentally whether heterodimers form.

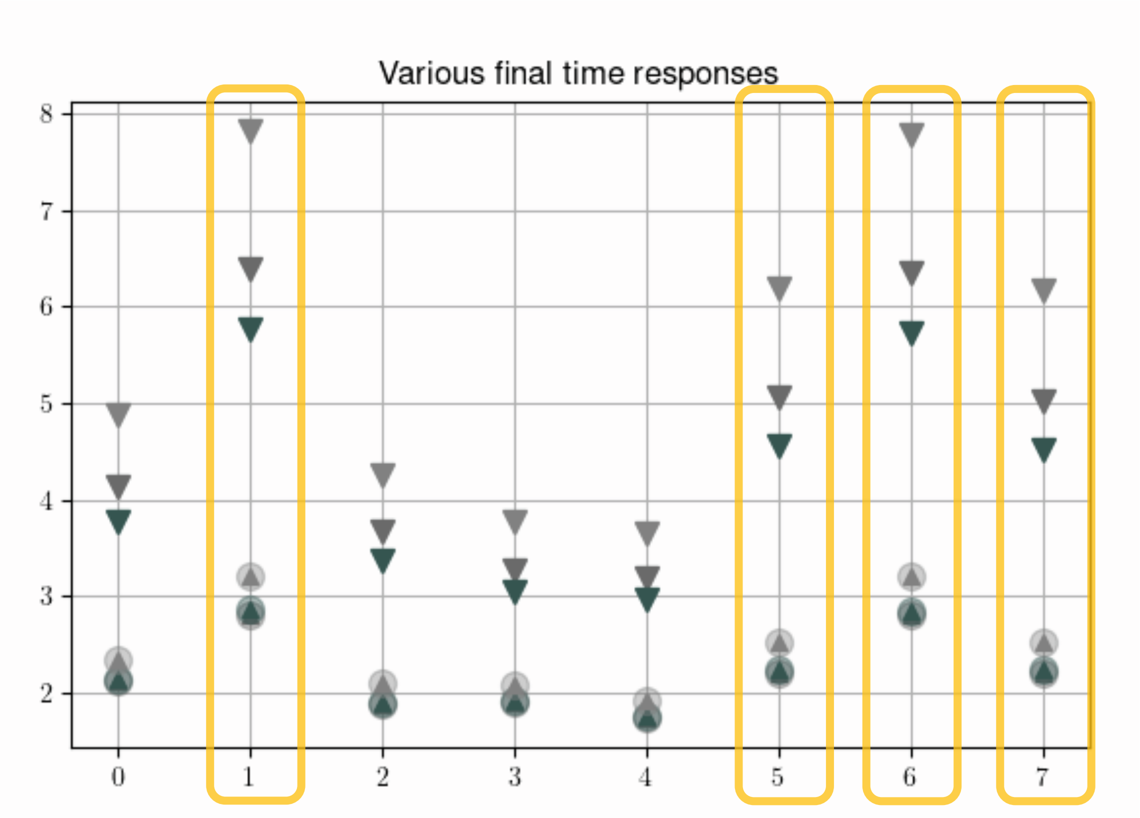

Supplemental Figure S14: One shade of grey for each of the 3-time courses. The final response value (downwards triangle) compared to its initial value (circle upwards triangle), for a range of conditions along the x-axis, specified as follows (only non-default parameter values are indicated):

**0:** Default parameters (identical to Figure 2).	**1:** no A1:A2 heterodimers	**2:** Kon1=10.	**3:** Koff1=0.1
**4:** Kon1=10, Koff1=0.1.	**5:** Kon1=10, no A1:A2 heterodimers	**6:** Koff1=0.1, no A1:A2 heterodimers	**7:** Kon1=10, Koff1=0.1, no A1:A2 heterodimers

To conclude, we assessed sensitivity (as defined above) in a similar way to response amplitude. The results, shown in Supplemental Figure S15 are remarkably consistent with those pertaining to response amplitude; quantitative changes in the ARF1 affinities are largely inconsequential, whereas the removal of MpARF1:MpARF2 heterodimer significantly improves sensitivity to auxin.

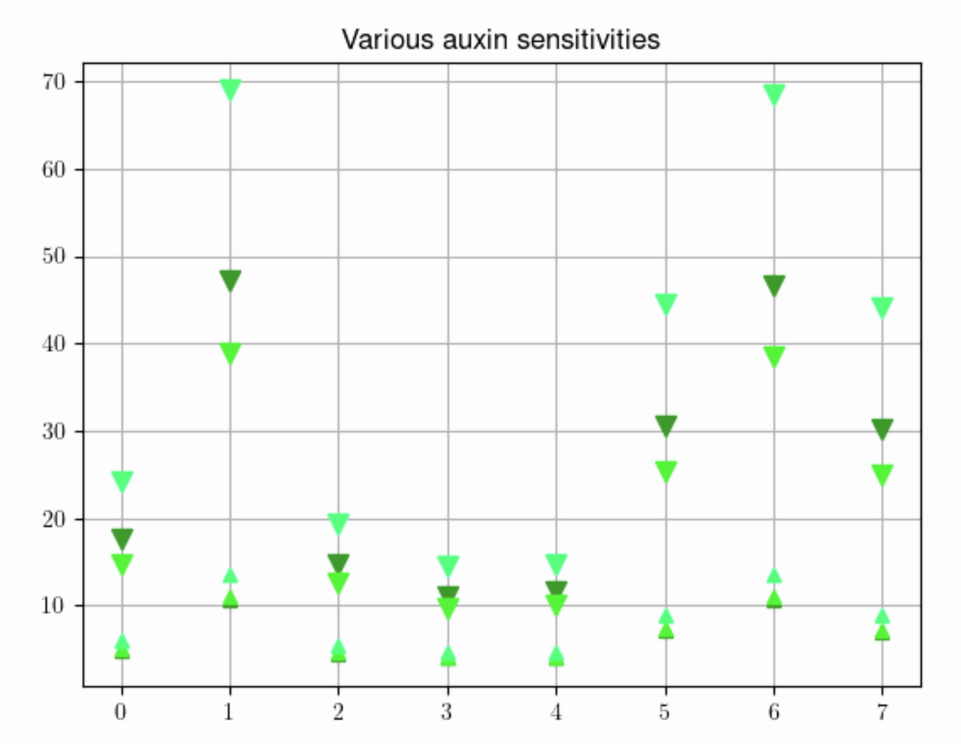

Supplemental Figure S15: One shade of green for each of the 3 time courses. The final sensitivity value (downwards triangle) compared to its initial value (upwards triangle), for a range of conditions along the x-axis, identically to Supplemental Figure S14.

References

Boer, D.R., Freire-Rios, A., van den Berg, W.A., Saaki, T., Manfield, I.W., Kepinski, S., Lopez-Vidrieo, I., Franco-Zorrilla, J.M., de Vries, S.C., Solano, R., Weijers, D., and Coll, M. (2014). Structural basis for DNA binding specificity by the auxin-dependent ARF transcription factors. Cell **156,** 577-589.

Farcot, E., Lavedrine, C., and Vernoux, T. (2015). A modular analysis of the auxin signalling network. PloS one **10,** e0122231.

Fontana, M., Roosjen, M., Crespo Garcia, I., van den Berg, W., Malfois, M., Boer, R., Weijers, D., and Hohlbein, J. (2023). Cooperative action of separate interaction domains promotes high-affinity DNA binding of Arabidopsis thaliana ARF transcription factors. Proceedings of the National Academy of Sciences of the United States of America **120,** e2219916120.

Han, M., Park, Y., Kim, I., Kim, E.H., Yu, T.K., Rhee, S., and Suh, J.Y. (2014). Structural basis for the auxin-induced transcriptional regulation by Aux/IAA17. Proceedings of the National Academy of Sciences of the United States of America **111,** 18613-18618.

Nanao, M.H., Vinos-Poyo, T., Brunoud, G., Thevenon, E., Mazzoleni, M., Mast, D., Laine, S., Wang, S., Hagen, G., Li, H., Guilfoyle, T.J., Parcy, F., Vernoux, T., and Dumas, R. (2014). Structural basis for oligomerization of auxin transcriptional regulators. Nature communications **5,** 3617.
