# Supplementary material for: Quantitative imaging reveals the role of MpARF proteasomal degradation during gemma germination
Source: Plant Commun. 2024 Jul 9;5(11):101039. doi: 10.1016/j.xplc.2024.101039 (PMC11589480; doi:10.1016/j.xplc.2024.101039)
Supplement: Document S2. Article plus supplemental information [file mmc2.pdf]

Plant Communications
Research article

CellPress
Partner Journal

# Quantitative imaging reveals the role of MpARF proteasomal degradation during gemma germination

Shubhajit Das[1,4], Martijn de Roij[1], Simon Bellows[2], Melissa Dipp Alvarez[1], Sumanth Mutte[1], Wouter Kohlen[3], Etienne Farcot[2], Dolf Weijers[1,*] and Jan Willem Borst[1,*]

[1]Laboratory of Biochemistry, Wageningen University and Research, Stippeneng 4, 6708WE Wageningen, the Netherlands

[2]School of Mathematical Sciences, University of Nottingham, University Park, NG7 2RD Nottingham, UK

[3]Laboratory of Molecular Biology, Wageningen University and Research, Droevendaalsesteeg 1, 6708PB Wageningen, the Netherlands

[4]Present address: Institute of Science and Technology, 3400 Klosterneuburg, Austria

***Correspondence: Dolf Weijers (dolf.weijers@wur.nl), Jan Willem Borst (janwillem.borst@wur.nl)**

https://doi.org/10.1016/j.xplc.2024.101039

## ABSTRACT

**The auxin signaling molecule controls a variety of growth and developmental processes in land plants. Auxin regulates gene expression through a nuclear auxin signaling pathway (NAP) consisting of the ubiquitin ligase auxin receptor TIR1/AFB, its Aux/IAA degradation substrate, and DNA-binding ARF transcription factors. Although extensive qualitative understanding of the pathway and its interactions has been obtained, mostly by studying the flowering plant *Arabidopsis thaliana*, it remains unknown how these translate to quantitative system behavior *in vivo*, a problem that is confounded by the large NAP gene families in most species. Here, we used the minimal NAP of the liverwort *Marchantia polymorpha* to quantitatively map NAP protein accumulation and dynamics *in vivo* through the use of knockin fluorescent fusion proteins. Beyond revealing the dynamic native accumulation profile of the entire NAP protein network, we discovered that the two central ARFs, MpARF1 and MpARF2, are proteasomally degraded. This auxin-independent degradation tunes ARF protein stoichiometry to favor gene activation, thereby reprogramming auxin response during the developmental progression. Thus, quantitative analysis of the entire NAP has enabled us to identify ARF degradation and the stoichiometries of activator and repressor ARFs as a potential mechanism for controlling gemma germination.**

**Key words:** auxin signaling, ARF degradation, *Marchantia polymorpha*, live cell imaging

**Das S., de Roij M., Bellows S., Alvarez M.D., Mutte S., Kohlen W., Farcot E., Weijers D., and Borst J.W.** (2024). Quantitative imaging reveals the role of MpARF proteasomal degradation during gemma germination. Plant Comm. **5**, 101039.

## INTRODUCTION

The plant signaling molecule auxin triggers a multitude of growth, developmental, and physiological responses across land plants (Teale et al., 2006; Vanneste and Friml, 2009). Key to the cellular response is a canonical nuclear auxin signaling pathway (NAP) (Lavy and Estelle, 2016). Auxin promotes the binding of AUXIN/INDOLE-3-ACETIC ACID (Aux/IAA) repressor proteins to the nuclear auxin receptor TRANSPORT INHIBITOR RESPONSE1/AUXIN SIGNALING F-BOX (TIR1/AFB) (Tan et al., 2007). This triggers degradation of Aux/IAA proteins (Gray et al., 2001) and releases DNA-binding AUXIN RESPONSE FACTORS (ARFs) (Weijers and Wagner, 2016; Leyser, 2018) from inhibition (Figure 1A). In past decades, the NAP has been studied extensively in the angiosperm *Arabidopsis thaliana* (Friml, 2022). These studies have led to a comprehensive qualitative model of auxin signaling, supported by atomic structures of each component. A key challenge in understanding auxin response is the large size of gene families representing each component in angiosperms (Luo et al., 2018). Specifically, it is hard to tell whether any one protein behaves typically or atypically. More recent analysis of the auxin response system in the bryophytes *Physcomitrium patens* (a moss) and *Marchantia polymorpha* (a liverwort) has helped to reduce system complexity and derive common core principles. Phylogenetic analysis has shown that the simple NAP system in *Marchantia* consists of one ARF in

Published by the Plant Communications Shanghai Editorial Office in association with Cell Press, an imprint of Elsevier Inc., on behalf of CSPB and CEMPS, CAS.


This is an open access article under the CC BY license (http://creativecommons.org/licenses/by/4.0/).

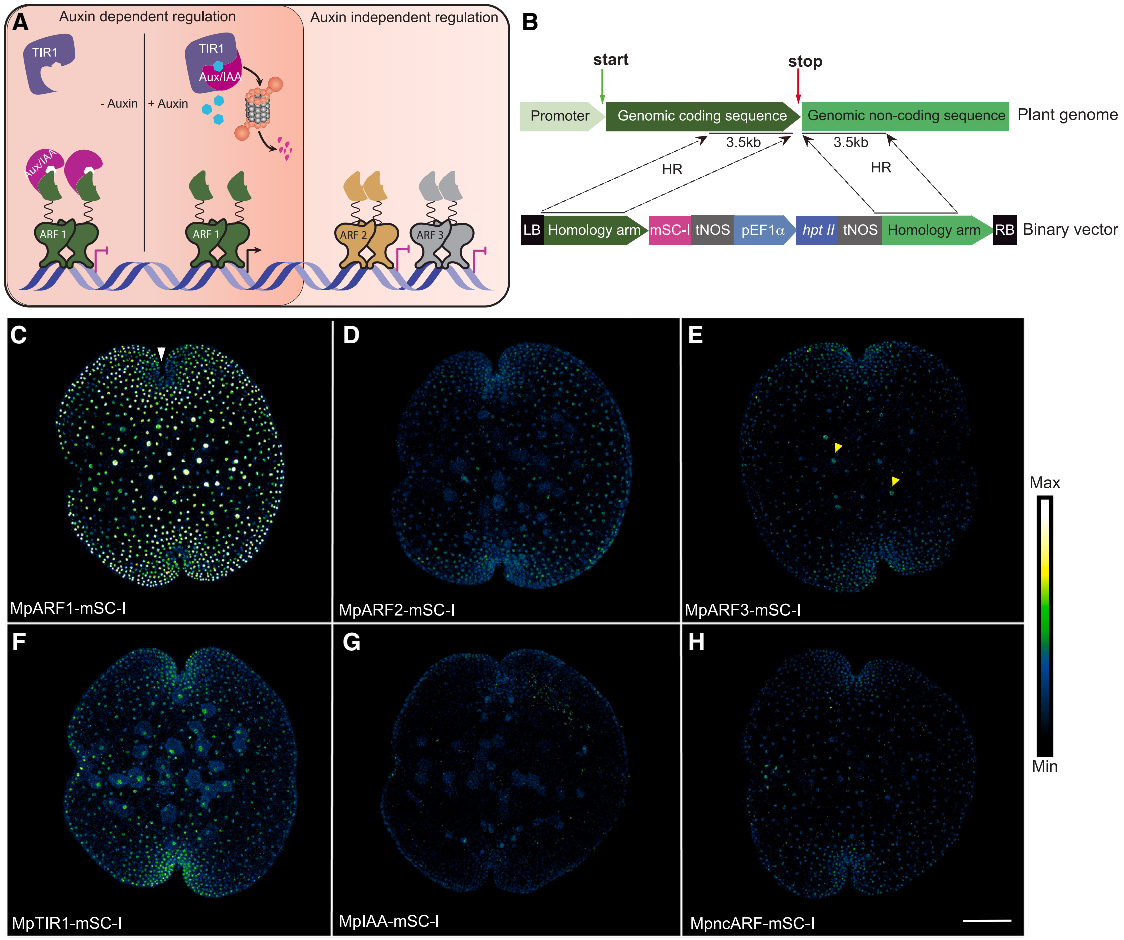


**Figure 1. Native cellular accumulation patterns of NAP signaling proteins in *Marchantia* gemmae.**

**(A)** Schematic diagram of the nuclear auxin signaling pathway. In the absence of auxin, Aux/IAA repressors interact with ARFs to inhibit transcriptional activation. In the presence of auxin, TIR1 interacts with Aux/IAA and targets it for proteasomal degradation. Free ARFs initiate gene transcription of auxin response genes. ARF2 is independent from this regulation. The regulation mode of ARF3 is not well characterized. Both ARF2 and ARF3 act as transcriptional repressors.**(B)** Principle of homologous recombination (HR)-mediated genomic knockin of fluorescent proteins at the C terminus of a gene of interest. *hptII*, hygromycin phosphotransferase; LB, T-DNA left border; mSC-I, mScarlet-I; pEF1α, ELONGATION FACTOR1α promoter; tNOS, nopaline synthase terminatormoter; RB, T-DNA right border; tNOS, nopaline synthase terminator.**(C–H)** Fluorescence patterns reflecting the accumulation of core nuclear auxin signaling proteins in dormant gemmae of *Marchantia* knockin lines with mScarlet-I. Scale bar, 100 μm. White arrowhead indicates the apical notch; yellow arrowheads indicate the rhizoid cells.

each of the three subclasses (A class: MpARF1; B class: MpARF2; C class: MpARF3), a single Aux/IAA (MpIAA), and a single TIR1 receptor (MpTIR1), in addition to a non-canonical ARF (MpncARF) and a non-canonical Aux/IAA (MpncIAA; Mutte et al., 2018). Studies in *Physcomitrium* and *Marchantia* led to the formulation of a model in which the auxin response revolves around antagonistic interactions between A- and B-class ARFs competing for the same DNA-binding sites. While A-ARFs are regulated by auxin and can switch between repression and activation, B-ARFs are auxin-independent repressors (Lavy et al., 2016; Kato et al., 2020). Thus, stoichiometry of the A- and B-class ARFs is predicted to determine the output of auxin response. The C-class ARF in *Marchantia* acts as an auxin-independent transcriptional repressor (Flores-Sandoval et al., 2018), and the non-canonical ARF (ncARF) that lacks the DNA-binding domain positively influences auxin response (Mutte et al., 2018). However, the mechanisms of C-class ARF DNA binding and ncARF-driven auxin response remain to be explored.

Despite our deep understanding of the NAP, one key element remains largely unresolved: essentially all *in vivo* findings are built on qualitative data, and the true *in vivo* concentrations and accumulation patterns of NAP components and their relative concentrations or stoichiometries are unknown. As a consequence, it is unclear whether domains of different auxin activities are generated and, if so, through what mechanisms. Likewise, it is nearly impossible to infer dynamics from genetic or *in vitro* biochemical data, which thus leaves many questions unanswered about the temporal aspects of auxin response. Given that any biochemical interaction is determined by both affinity and concentration of and among components, it will be essential to map protein patterns *in vivo*. The ability to tag endogenous proteins through homologous-recombination-based gene targeting in bryophytes enables us to visualize the dynamics of proteins at native concentrations, as a first step toward understanding system behavior.

In this study, we generated and characterized fluorescent genomic knockin lines for all *Marchantia* NAP proteins. In addition to resolving spatial and temporal maps of protein accumulation, this enabled us to identify active proteasomal degradation of both MpARF1 and MpARF2. We show that degradation tunes A-/B-ARF stoichiometry to permit normal development and auxin response. Our study provides a unique resource for the quantitative analysis of auxin response in *Marchantia* and reveals proteasomal degradation as a regulatory mechanism for ARFs.

## RESULTS

### Development of a collection of genomic knockin lines for the *Marchantia* NAP

Essentially all activities and properties underlying protein function are dependent upon protein concentration. Therefore, a quantitative understanding of any biological process requires the monitoring of endogenous protein accumulation patterns, which can be achieved either by immunolabeling—which suffers from difficulties in reproducible quantification across different proteins—or by endogenous tagging. The latter has so far not been feasible in flowering plants because of the extremely low efficiencies of homology-directed gene targeting, but it is realistic in bryophytes. We set out to generate genomic knockin translational fluorescent fusion lines for all components of the NAP in *Marchantia* (Figure 1B) (Ishizaki et al., 2013). We previously generated knockin lines for both MpARF1 and MpARF2, fused to mScarlet-I (Kato et al., 2020). We extended this, and using homologous recombination, we knocked in a fluorescent protein (either mNeonGreen or mScarlet-I) at the C terminus of each auxin signaling protein: MpARF1 (class A), MpARF2 (class B), MpARF3 (class C), MpIAA, MpTIR1, and MpncARF, thus effectively replacing the single endogenous copy with a fluorescently tagged one.

Where analyzed, overexpression and loss of function of these proteins have been shown to cause strong phenotypes (Flores-Sandoval et al., 2015; Kato et al., 2015, 2017, 2020; Mutte et al., 2018; Suzuki et al., 2023) In contrast, all knockin lines displayed macroscopically normal morphologies and responded to externally applied auxin in a manner qualitatively similar to that of the wild type (Supplemental Figure 1). This suggests that all fusion proteins are functional and that none accumulate at levels significantly outside of the normal range. Next, we used confocal microscopy to visualize all auxin signaling proteins in dormant gemmae residing within the gemma cup, and we compared fusions of all NAP components to the same fluorescent protein (mScarlet-I) under identical experimental conditions. All three ARFs localized to nuclei, but their tissue-wide accumulation patterns showed clear differences (Figures 1C–1E). MpARF1-mScarlet-I showed the highest accumulation among all the NAP components and was present in most cell types of the gemma (see explanation of different cell types in Supplemental Figure 2). MpARF2-mScarlet-I accumulation was much weaker and was absent in the region distal of the apical notch where rhizoid initials reside. MpARF3-mScarlet-I (class C) displayed a weak yet broad accumulation pattern in all cell types. The auxin receptor MpTIR1-mScarlet-I and the non-canonical MpncARF-mScarlet-I showed low, nuclear accumulation, and MpIAA-mScarlet-I was not detected at this stage (Figures 1F–1H). In summary, we established a collection of knockin lines that enabled mapping and quantification of NAP protein accumulation patterns *in vivo*.

### Co-receptor-ligand dynamics in *Marchantia* NAP

We could not detect MpIAA in dormant gemma (Figure 1G), and therefore we monitored MpIAA accumulation patterns in the first 24 h following gemma germination. The first weak nuclear signals could be detected after 24 h of growth (Figure 2A). It is conceivable that the MpIAA protein is expressed in dormant gemmae but continuously degraded owing to high auxin concentrations. Indeed, treatment of dormant gemmae with the proteasome inhibitor MG132 led to a clear nuclear signal within 2 h (Figure 2B). By quantifying free IAA concentrations in germinating gemmae, we found a clear decline in the 24 h following germination (Figure 2D), consistent with IAA-triggered MpIAA degradation at early stages. Pharmacological inhibition of auxin synthesis by combining l-kynurenine and yucasin inhibitors (He et al., 2011; Nishimura et al., 2014) led to abundant nuclear MpIAA-mScarlet-I accumulation across gemmae within 2 h (Figure 2C). These findings suggest that MpTIR1 is active throughout gemma development in mediating IAA-triggered MpIAA degradation. MpTIR1-mScarlet-I was detectable at all stages and showed a gradual increase in protein levels as gemmae grew (Figure 2E). Thus, as gemmae break dormancy and grow, the auxin response system moves from restrictive to permissive.

The pace of auxin signal relay from TIR1/AFBs to ARFs depends on the degradation rate of Aux/IAA proteins. A quick transcriptional response to auxin, therefore, relies on the rapid degradation of Aux/IAAs (Dreher et al., 2006). Aux/IAA degradation rates in *Arabidopsis*, where studied, range from 10 min to 1.3 h, and it is unclear what rate reflects the ancestral state within the Aux/IAA family. To determine the *in vivo* MpIAA half-life, we first depleted endogenous auxin with l-kynurenine and yucasin (Figure 3A). This led to abundant nuclear accumulation of MpIAA. Gemmae were next treated with 3 $\mu$M of the stable, synthetic auxin 1-NAA (1-naphthaleneacetic acid), and MpIAA protein levels were tracked and quantified by time-lapse imaging (Figure 3B). We detected a clear loss of fluorescence signal and determined the half-life of MpIAA to be 6.5 min (Figure 3C). To confirm that the signal decline was due to proteasomal degradation, we added MG132 and found that the fluorescence signal remained over the time course (Supplemental Figure 3). Likewise, the signal of MpARF1-Scarlet-I did not decline over the same time with the same imaging settings (Supplemental Figure 4). This demonstrated that the imaging settings used did not induce significant photobleaching. These results suggest that the ancestral condition of the NAP may be characterized by fast Aux/IAA turnover and that the slower degradation rates found in *Arabidopsis* may be a derived property.

### Dynamic MpARF protein accumulation

Dynamics in the NAP are ascribed primarily to the degradation of Aux/IAA proteins, whereas TIR1 and ARFs are considered to

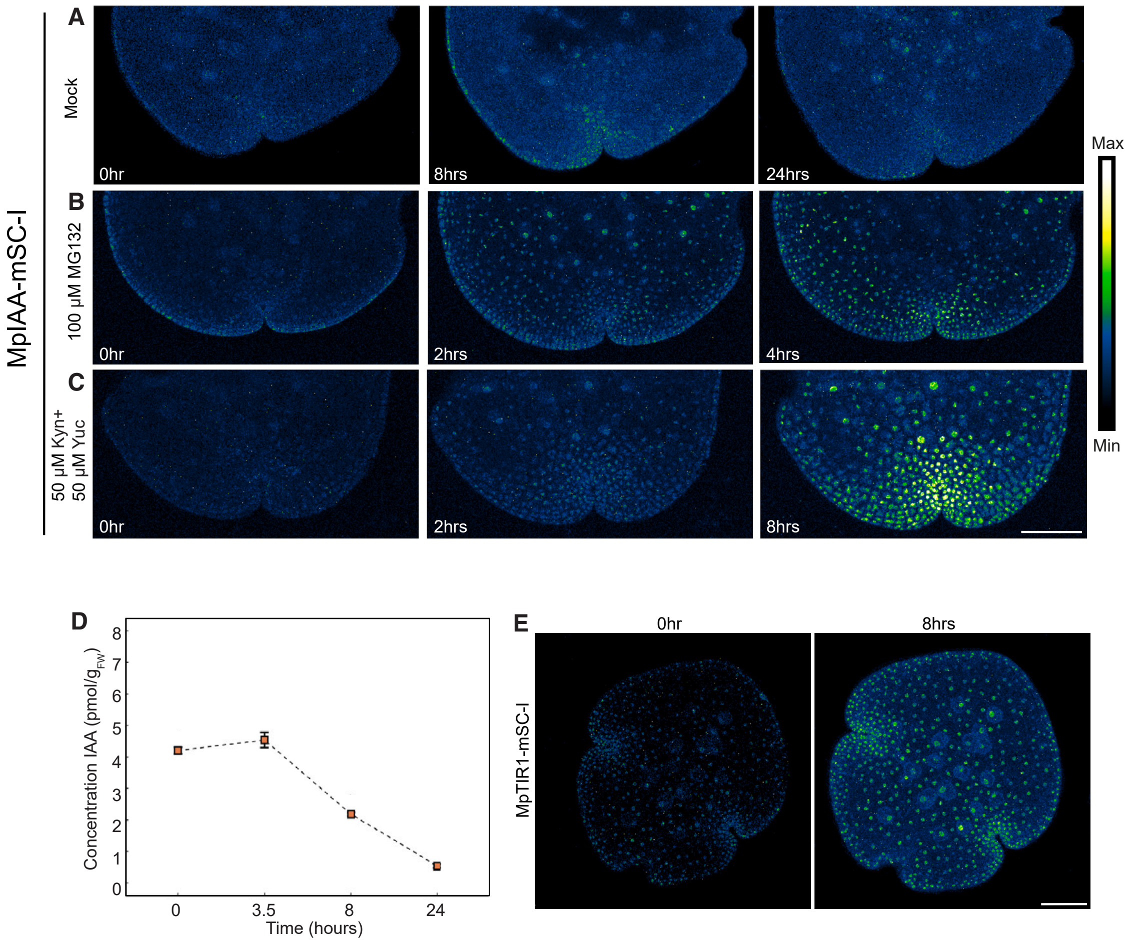


**Figure 2. Concentrations of auxin and MpIAA are dynamic during gemma development.**

**(A–C)** Time-course imaging of MpIAA-mSC-I treated with mock **(A)**, 100 μM MG132 **(B)**, and 50 μM l-kynurenine and 50 μM yucasin **(C)**.**(D)** Concentration dynamics of free IAA in germinating gemmae ($n$ = 3, error bars: SD). Liquid chromatography–mass spectrometry quantification shows that total IAA levels drop following gemma germination.**(E)** Time-course imaging of MpTIR1-mSC-I shows that its accumulation levels gradually increase during gemma germination. Scale bars, 100 μm.

be more stable factors. Indeed, transcriptional levels of MpARFs were stable following gemma germination (Figure 4A). To explore the accumulation dynamics of the MpARF proteins, we imaged each ARF during the first 24 h following germination. Remarkably, we found that both MpARF1 and MpARF2 protein levels progressively declined (Figures 4B, 4C, and Supplemental Figure 5). By contrast, MpARF3 patterns did not change in this time window (Figure 4D). Given the higher starting levels of MpARF1 in dormant gemmae and the lower MpARF2 concentrations, the former was still detectable after 8 h, whereas the MpARF2 signal declined to undetectable levels (Supplemental Figure 5A). Comparable dynamics were found for MpARF1-mNeonGreen and MpARF2-mNeonGreen (Supplemental Figure 5), indicating that signal decline was independent of the fluorescent protein tag.

Given that MpARF transcript levels did not change in the same time window (Figure 4A), MpARF1 and MpARF2 must be post-transcriptionally controlled by either translational or post-translational regulation. To test the involvement of proteolysis, we treated plants with the proteasome inhibitor MG132 or bortezomib. While MpARF3 was unaffected by the treatments (Figures 4D and 4E), the decline of MpARF1 was prevented by either inhibitor (Figures 4B, 4C, 4E, and Supplemental Figure 6). MpARF2 levels not only stabilized upon treatment with the inhibitors but even increased relative to those of dormant gemmae (Figures 3B, 3C, 3E, and Supplemental Figure 6). This implies that both MpARF1 and MpARF2, but not MpARF3, are proteasomally degraded.

Proteolysis of regulatory proteins, including transcriptional regulators, is often part of feedback control or a point of regulation in signaling pathways. To investigate whether auxin or any other known signaling molecule triggers MpARF degradation during germination, we exogenously treated gemmae for ±16 h with auxin and a range of other plant hormones or inhibitors. Neither auxin nor abscisic acid, gibberellic acid, or jasmonic acid altered MpARF degradation. Similarly, auxin transport or biosynthesis inhibitors did not affect degradation (Supplemental Figure 7), suggesting that ARF degradation is not controlled by auxin or other tested hormones.

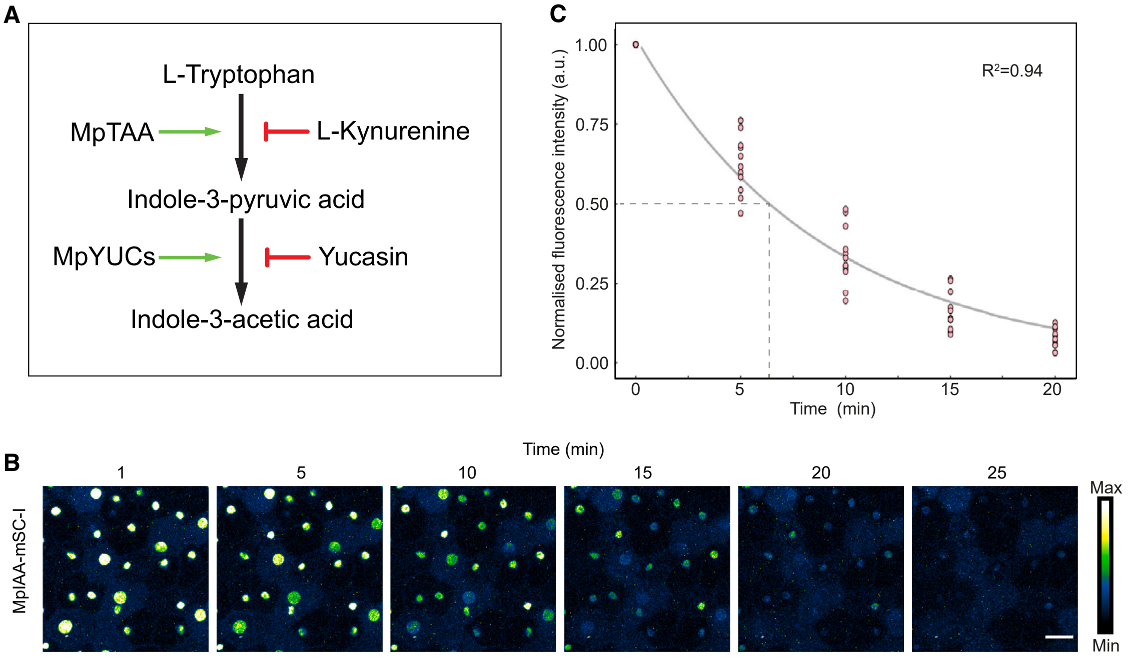


**Figure 3. Quantification of MpIAA turnover.**
**(A)** Auxin biosynthesis via the indole pyruvic acid pathway is catalyzed by two key enzymes, TRYPTOPHAN AMINOTRANSFERASE OF ARABIDOPSIS (TAA1) and YUCCA (YUC). To lower auxin levels in a tissue, these enzymes can be chemically inhibited by l-kynurenine and yucasin, respectively.**(B)** Time-lapse imaging of MpIAA-mSC-I gemmae pre-treated with 50 μM l-kynurenine and 50 μM yucasin. Upon auxin (1-NAA; 3 μM) treatment, fluorescence rapidly decreases owing to MpIAA-mSC-I degradation.**(C)** Fitting normalized fluorescence data using a 1-component exponential model results in an MpIAA half-life of 6.5 min. Scale bar, 25 μm.

## Regulated degradation controls ARF stoichiometry for normal development and auxin response

A key question is what function is served by the regulated degradation of MpARF1 and MpARF2. We previously proposed that auxin responsiveness in *Marchantia* is determined by the stoichiometry of MpARF1 and MpARF2 (Kato et al., 2020). We therefore explored the possibility that this stoichiometry is actively regulated by proteolysis. Because such stoichiometries may differ among individual cells, it is not possible to derive them by comparing individual knockin lines. To visualize both MpARF1 and MpARF2 quantities in the same tissue and in the same cell, we crossed MpARF1-mScarlet-I with MpARF2-mNeonGreen and MpARF1-mNeonGreen with MpARF2-mScarlet-I and obtained two double knockin lines. Both double knockins showed ARF accumulation patterns comparable to those of the single MpARF lines (Figures 4B, 4C, and 5A–5C). As expected from their individual patterns, MpARF1 and MpARF2 were present in different stoichiometries in different cell types. In nuclei of meristematic apical notch cells, MpARF2 displayed higher accumulation than MpARF1 (Figure 5A–5C). In rhizoid initial cells, MpARF2 protein was nearly undetectable, whereas MpARF1 showed clear accumulation (Figure 5D). In the region separating the apical notch and the rhizoid initial cells, which we refer to here as the transition zone, both MpARFs were present (Figure 5A and 5B and Supplemental Figure 8).

We next determined MpARF1/MpARF2 stoichiometries following gemma germination in an MpARF1-mSc-I/MpARF2-mNG double knockin line. For this analysis, we measured relative MpARF1:MpARF2 fluorescence intensities in individual nuclei within four independent gemmae at four time points. Quantification of the MpARF1 and 2 fluorescence intensities in the transition zone showed that the MpARF1:MpARF2 stoichiometry consistently increased during gemmae germination, favoring MpARF1 over MpARF2 (Figure 5E).

We next asked what the impact of this change in MpARF1:MpARF2 stoichiometry might be, and we first developed a mathematical model of the minimal *Marchantia* NAP and its known interactions (Supplemental File 1). Using a piecewise polynomial function, we fit three independent, experimentally quantified MpARF accumulation profiles into the mathematical model, in which the effect of changing MpARF1 and MpARF2 levels on the outcome of auxin response was modeled as the transcription of a hypothetical ARF-regulated gene. Model simulations predicted that the increased MpARF1:MpARF2 ratio would enhance transcriptional response output (solid lines, Figure 5F). Simulated loss of MpARF2 degradation predicted no response output (dashed lines, Figure 5F). Thus, ARF degradation may be required to switch auxin-regulated genes from an inactive to an active transcriptional state by acting on the MpARF1:MpARF2 stoichiometry.

To test the prediction that regulated, low MpARF2 levels are needed for proper auxin sensitivity, we made use of the published MpARF2 inducible overexpression lines (Kato et al., 2020). In addition, we generated a new set of stable overexpression lines with and without a C-terminal citrine tag. Induction of MpARF2-GR activity with dexamethasone prevented thallus growth (Figure 5G) (Jones and Dolan, 2012; Flores-Sandoval et al.,

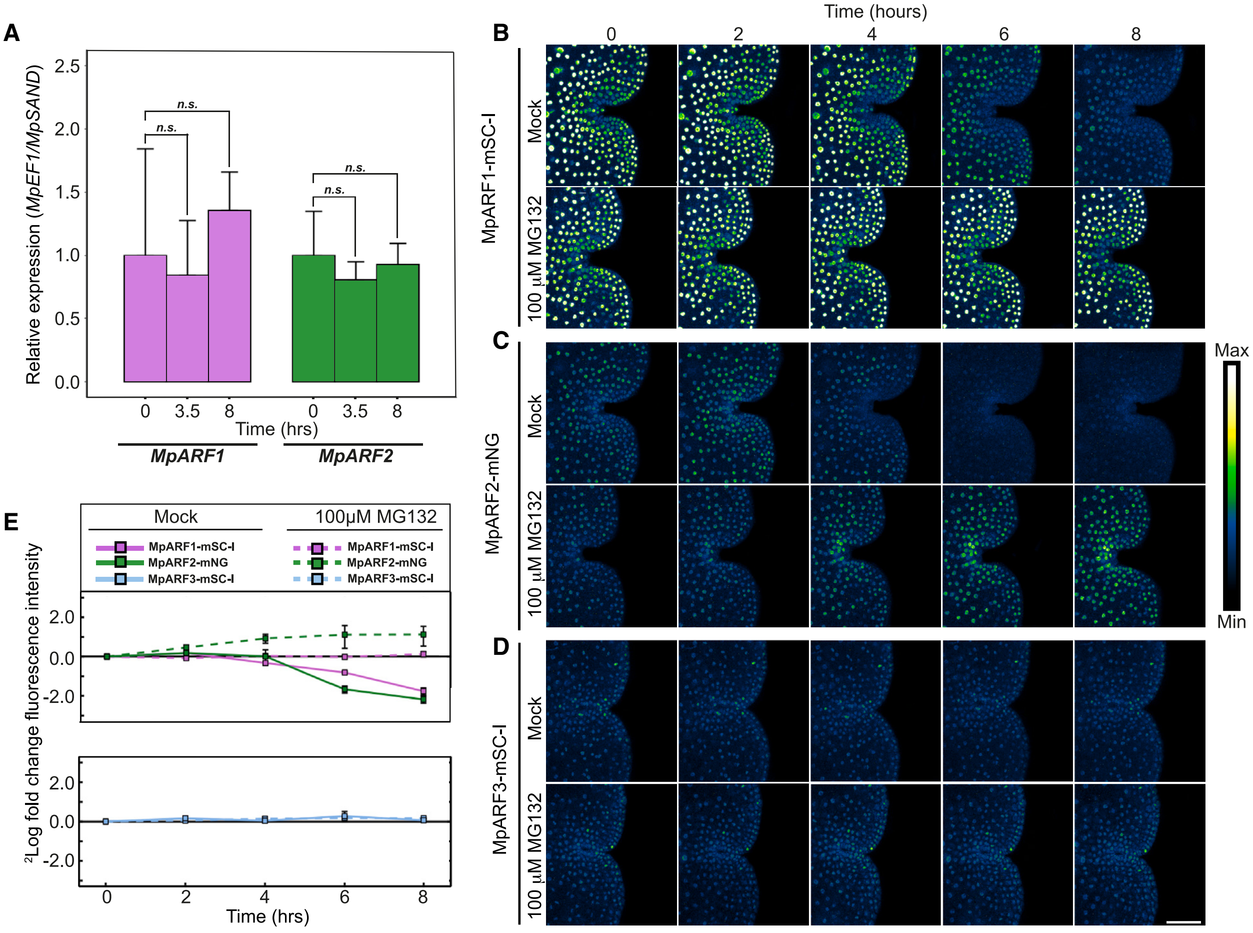


**Figure 4. MpARF protein accumulation in gemmae is post-translationally regulated by the proteasome.**
**(A)** qRT–PCR of *MpARF1* and *MpARF2* transcripts shows stable gene transcription during gemma germination ($n = 3$, Student's $t$ test $p < 0.05$).**(B–D)** Time-course imaging of MpARF1, MpARF2, and MpARF3 protein fusions to mScarlet-I in germinating gemmae in the control treatment or upon treatment with 100 μM MG132.**(E)** Quantification of MpARF1, MpARF2, and MpARF3 protein accumulation after germination, expressed as FC relative to that in dormant gemmae (≥30 nuclei quantified per image, $n = 3$, error bars: SE). Scale bar, 50 μm.

2015). Likewise, 2-week-old stable pEF1-MpARF2 overexpression lines had multiple apical notches, were strongly defective in growth, lacked gemma cups (Supplemental Figure 9), and were completely insensitive to auxin treatment (1-NAA; Figure 5H and Supplemental Figures 6A and 6B). The inducible ARF2-GR lines had a lower expression of auxin response genes (Kato et al., 2020). In addition, in previous reports, overexpression of MpARF1 has been shown to cause auxin hypersensitivity and increased branching rates (see Eklund et al., 2015; Kato et al., 2020). These results support the prediction that elevated levels of MpARF1 and MpARF2 have opposite effects on auxin sensitivity.

## Transcriptional reprogramming in germinating gemmae

The dynamics of NAP components, particularly the shift in ARF stoichiometry following gemma germination, suggest that auxin-regulated genes are dynamically controlled. To test this prediction directly, we performed an RNA-sequencing (RNA-seq) experiment on gemmae collected at 0 h (dormant), 3.5 h, and 8 h of growth. Principal-component analysis (PCA) resulted in a strong clustering of biological replicates and a high variance between different time points. This suggests major differences in the transcriptome at different developmental stages and implies massive transcriptional reprogramming early during gemma germination (Figure 6A). After 3.5 h of gemma growth, we found hundreds of differentially regulated genes, and this effect was even more pronounced after 8 h (Figure 6B). There was a large overlap in genes that were either up- or downregulated at 3, 5, and 8 h compared with the 0-h time point (Figure 6C). However, a substantial fraction of genes was also uniquely regulated at each time point (Figure 6C). We next identified 94 known auxin-regulated genes from available transcriptome data of auxin-treated wild-type plants (Mutte et al., 2018) and determined their expression levels across the time series. Transcript levels of the majority of auxin-upregulated genes were higher following germination, and likewise, transcript counts of most auxin-downregulated genes were lower in germinated gemmae (Figure 6D). Thus, as predicted, at a global scale, transcriptional auxin response is elevated upon dormancy release. However, there also seems to be a dampening of auxin-regulated gene expression at 8 h, which suggests that there is additional complexity at this time point that our model does not capture. To directly test the capacity for

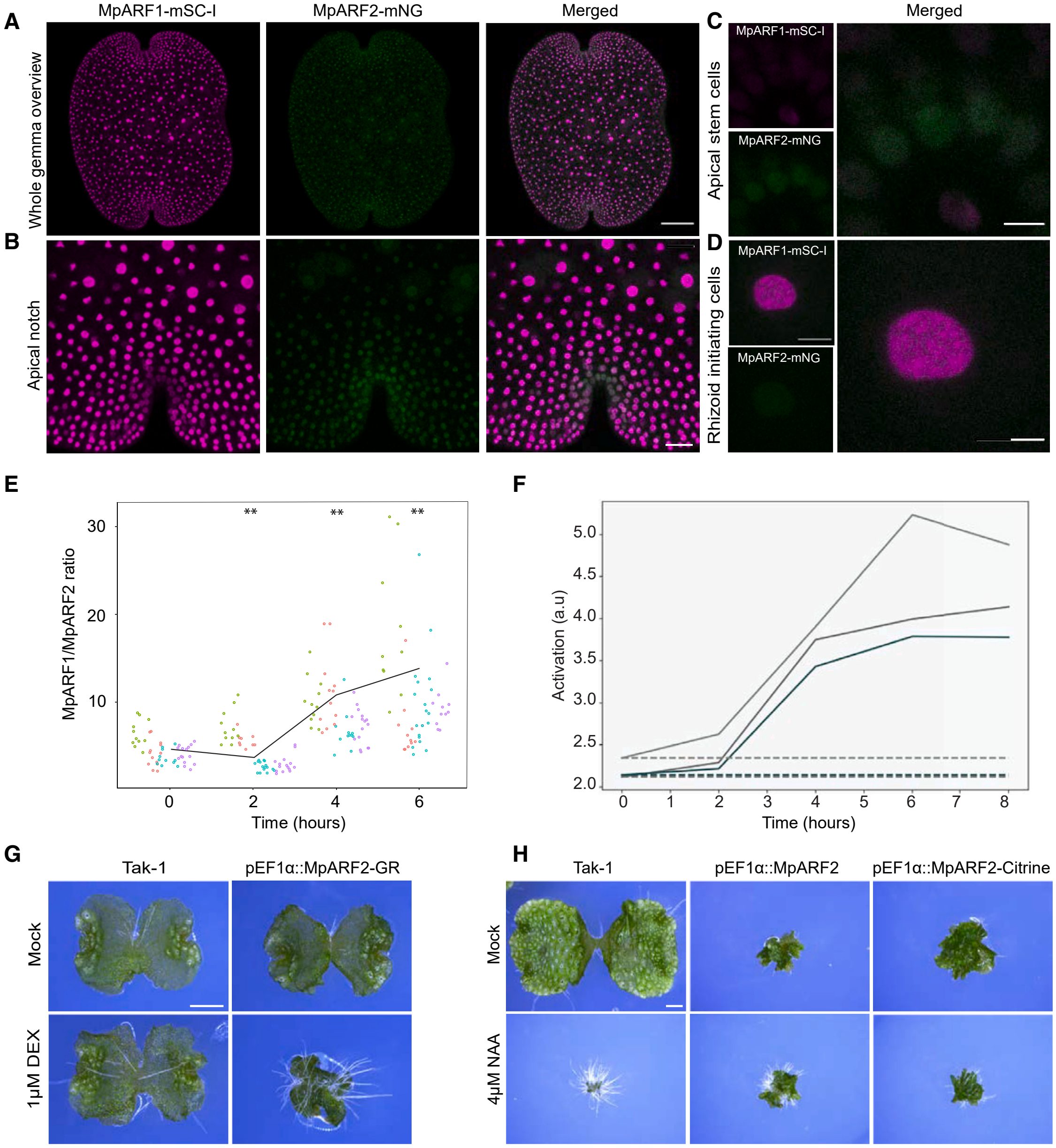


**Figure 5. Control of gemma growth by regulation of MpARF1/MpARF2 stoichiometry.**
**(A)** Overview of MpARF1-mSC-I and MpARF2-mNG accumulation patterns in dormant gemmae of double knockin lines (scale bar, 100 μm).**(B–D)** Detail of MpARF1-mSC-I and MpARF2-mNG accumulation patterns in the apical notch region (**B**; scale bar, 25 μm), outermost apical notch cells (**C**; scale bar, 5 μm), and rhizoid initial cells (**D**; scale bar, 5 μm).**(E)** Quantification of ARF1:ARF2 stoichiometry in individual nuclei of a double knockin line (MpARF1-mScI MpARF2-mNG) during gemma germination. Colors mark nuclei from different gemmae. $^{**}p < 0.05$, paired Student's $t$ test between t = 0 and other time points.**(F)** Predicted transcription pattern of an auxin-inducible gene (a.u.) during gemma germination. Solid lines indicate transcription rate under normal conditions in which MpARFs degrade during gemma germination, whereas dashed lines indicate transcription rate in the absence of MpARF2 proteasomal degradation. Lines correspond to predictions modeled in 3 replicate measurements of ARF levels.**(G)** Phenotypes of wild-type and pE-F1ARF2-GR lines treated for 7 days with 1 μM dexamethasone or mock control.**(H)** Phenotypes of 14-day-old wild type and untagged (pEF1αARF2) or citrine-tagged (pEF1αARF2-citrine) constitutive ARF2 overexpression lines grown on mock medium or medium containing 4 μM 1-NAA. Scale bars, 1 mm.

auxin-regulated gene expression at different times during gemma germination, we harvested gemmae either 1 or 3.5 h after removal from the gemma cup, treated each set separately with 1 μM IAA for 1 h, and then performed RNA-seq analysis. We determined differential expression upon IAA treatment at each time point and plotted the fold change (FC) for each gene at the two time points (Figure 6E). This analysis showed that among 289 auxin-regulated genes (152 at 1 h and 137 at 3.5 h), 26 showed similar regulation at both time points. Notably, there was no substantial feedback regulation of NAP components, including MpIAA (Supplemental Figure 10). Of the 26 genes with similar auxin regulation at both time points,

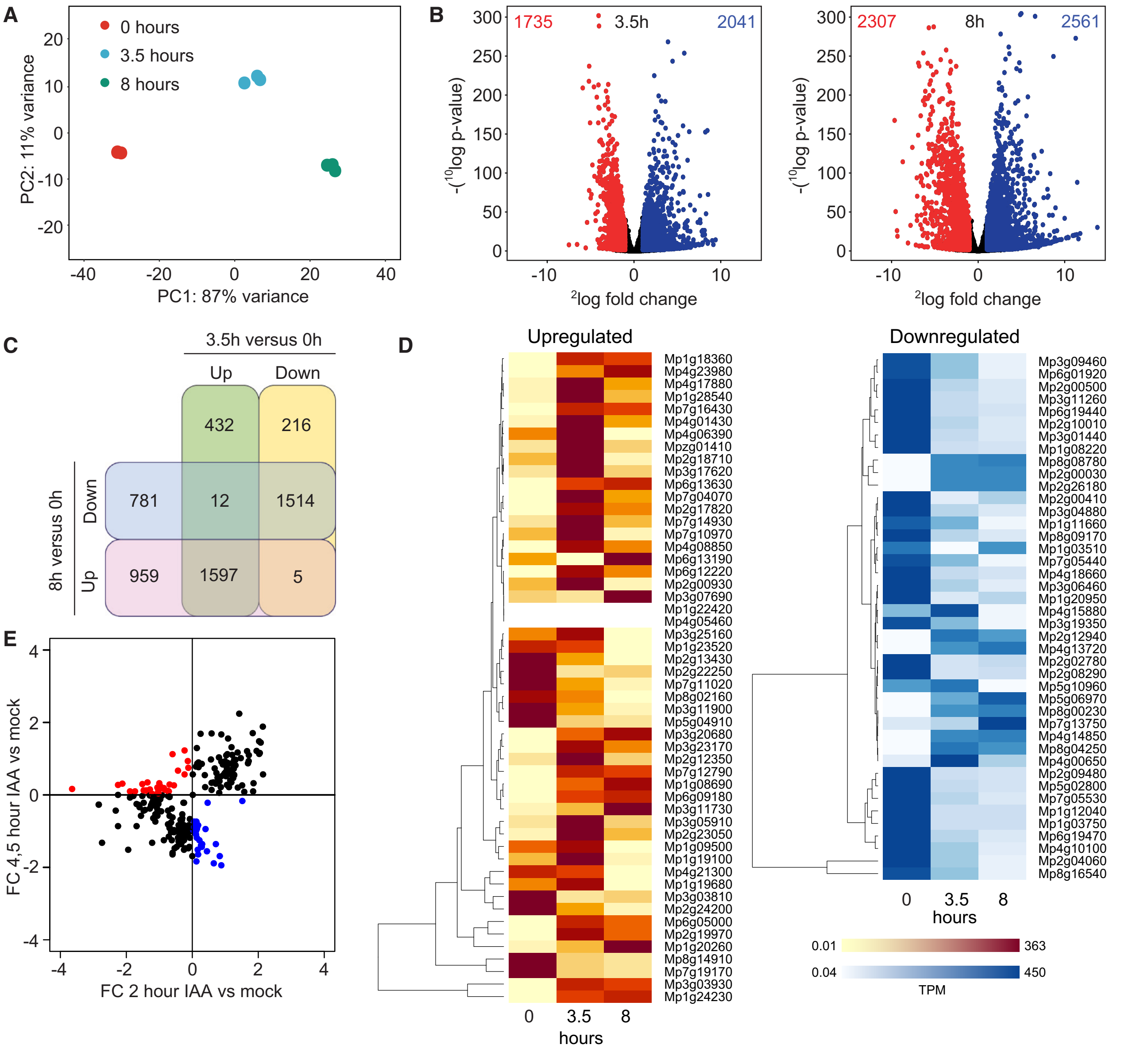


**Figure 6. Reprogramming of transcriptional auxin response capacity during early gemma development.**
**(A)** PCA plot of RNA-seq transcriptomes of dormant gemmae and gemmae grown in control medium for 3.5 and 8 h.
**(B)** Volcano plots show the number of significantly upregulated (blue) and downregulated (red) genes (padj < 0.05) at 3.5 and 8 h relative to the 0-h time point. **(C)** Venn diagram illustrates the overlap of DEGs in 3.5- and 8-h gemmae. **(D)** Transcriptional dynamics (TPM) of auxin-responsive genes during gemma germination. **(E)** Plot of $\log_2$(FC) differently regulated genes between 2- and 4.5-h-old gemmae treated with mock medium or auxin. Plot shows FC in average TPM counts of all differentially regulated genes at the 2 time points. Commonly regulated genes are marked in black; genes with opposite regulation at 2 and 4.5 h are indicated in blue and red, respectively.

a substantial fraction showed a clear difference in amplitude between time points. This result demonstrates that, as time passes after gemma germination, the capacity for auxin-regulated gene expression changes both qualitatively (identity of genes) and quantitatively (amplitude of regulation).

## DISCUSSION

We used the simple, minimal auxin response system of *Marchantia* to map protein levels both quantitatively and qualitatively throughout gemma development. To this end, we generated a full set of genomic knockin lines in which fluorescent proteins (two spectrally unique fluorophores) were fused to the C terminus of each auxin signaling protein by homologous recombination. Such an effort would also be possible in *Physcomitrium patens*, given the ease of gene targeting in that species (Roberts et al., 2011), but it would be much more complicated owing to the larger gene families. We do not know of any other plant species that would currently allow such a holistic investigation of the auxin response system. Given their macroscopically normal phenotypes, we believe that these knockins faithfully represent the native spatiotemporal accumulation profile of all nuclear auxin signaling proteins in *Marchantia*.

A noticeable pattern in MpARF spatial expression was the cell-specific difference in ARF quantities. Cell types with either A-class ARF1, B-class ARF2, or both, were observed. Because class A and B ARFs can recognize the same DNA-binding element in *Arabidopsis*, *Physcomitrium*, and *Marchantia* (Boer et al., 2014; Freire-Rios et al., 2020), the relative concentrations of these two competing MpARFs should determine auxin responsiveness. The contrasting localization maps of MpARF1 and MpARF2 in various cell types satisfy the protein abundance requirements to support an ARF stoichiometry-dependent model of gene regulation (Supplemental Figure S8). The relatively high MpARF1 accumulation observed in rhizoid initial cells points toward an ARF1-dominated gene activation mode. In the apical cells, we observed the opposite pattern—relatively high MpARF2 abundance, suggesting a gene repression mode. A plausible explanation is that auxin levels are high in stem cells, resulting in the degradation of MpIAA and relieving any auxin-dependent repression. To keep these stem cells transcriptionally silent and possibly to maintain stem cell identity, MpARF2 expression is relatively high. In the transition zone, both MpARF1 and MpARF2 accumulate, suggesting that this region is defined by a competitive ARF-DNA binding mode. MpARF-DNA binding assays and transcriptome analysis at a single-cell resolution would help determine the actual ARF output at cellular resolution.

By following the localization of all NAP components over time, we characterized the dynamics of the MpTIR1 receptor, the MpIAA repressor, and IAA itself, which suggested a progressive transition from a state with high auxin, low receptor, and low MpIAA to one with low auxin, high receptor, and higher MpIAA. Thus, the capacity for MpIAA-dependent auxin response changes dynamically during development. It could be that the gemma cup itself provides a high-auxin environment, as proposed previously (Eklund et al., 2015), and causes effective Aux/IAA degradation in gemmae. The capacity to respond to auxin depends on both the TIR1–Aux/IAA machinery and the ARF transcription factors (Weijers and Wagner, 2016; Leyser, 2018). As auxin at high concentration is a growth inhibitory signal and is required to keep gemmae dormant inside gemma cups (see Eklund et al., 2015), a reduction in auxin is required to release dormancy and allow gemma growth. As MpARF1:MpARF2 stoichiometry increases post-germination, transcriptional responses are permitted at an auxin concentration that is more favorable for growth. While imaging native MpARF levels, we found a surprising and profound degradation process that changes the ARF landscape during the first hours of gemma germination. Permissive auxin conditions at the early stage are countered by an ARF landscape that is relatively inhibitory. Over time, this ARF landscape shifts to a relatively activating state. The prediction would be that as time progresses, gemmae will become more sensitive to small changes in auxin concentrations, but this prediction remains to be tested.

We observed an elevated auxin response after dormancy release (Figure 6). By contrast, the quantified endogenous auxin levels were lower after 6 h of growth (Figure 2D). To explain this contradiction, we must consider the genes that are regulated exclusively by MpARF1 or MpARF2. MpARF1 is an auxin-dependent activator, whereas MpARF2 is an auxin-independent transcriptional repressor. Genes activated by MpARF1 alone are therefore expected to be downregulated when auxin levels are low after germination. Genes repressed by MpARF2 are not expected to change in expression with changes in auxin level, but as the MpARF2 protein is degraded, these genes are expected to be de-repressed. Genes regulated by both MpARF1 and MpARF2 are upregulated as their relative ratio increases, favoring MpARF1 function. In addition, we also observed increased expression of MpTIR1 upon dormancy release, suggesting modulation of the auxin response system at multiple points. Therefore, the transcriptional output is a reflection of not only the auxin level itself but also the relative concentrations of the signaling proteins and their stability at the stage of growth that caused this contradictory response.

We could connect MpARF degradation to an active control of A-/B-ARF stoichiometry. Even in the absence of any treatment, and in static observations, gemmae represent a rich landscape of MpARF1:MpARF2 stoichiometries. From first principles, and supported by mathematical modeling, these sites of varying stoichiometries should translate into areas with different auxin response outputs. Indeed, we see that the cells with the most "activating" stoichiometry are rhizoid initial cells, which are known to be highly sensitive to externally applied auxin (Ashton et al., 1979; Sakakibara et al., 2003; Prigge et al., 2010). Thus, the endogenous MpARF accumulation patterns will likely translate to a corresponding map of auxin sensitivities. Unfortunately, these are hard to map at present owing to the absence of a cellular-resolution reporter for mapping of the auxin response output. Given that stoichiometry manipulation does prevent normal auxin response and development, we do expect that the maps, both in the gemma studied here and beyond, will be an exciting starting point for mapping sites of auxin action.

A key question is how changes in ARF stoichiometry are brought about. In principle, any gene/protein regulatory process can contribute to protein accumulation, and this stoichiometry therefore serves as a central pivot point for the control of auxin output. Although it remains to be seen what transcriptional inputs contribute to diverse MpARF gene expression patterns, we do see that, given unequal starting levels, a relatively generic degradation rate can create large changes in MpARF1:MpARF2 stoichiometry. Identification of components in the degradation mechanism, as well as in (post)transcriptional controls such as the microRNA390–TAS3–ARF pathway (Xia et al., 2017), will help to resolve the tuning mechanisms.

MpARF1 and MpARF2 are the sole representatives of the A-class and B-class ARFs in *Marchantia*, and both are derived from an inferred algal proto-A-/B-ARF that is likely represented by ARF proteins of streptophyte algae (Mutte et al., 2018). It is thus plausible that proteolytic degradation is a property inherited from the proto-A-/B-ARF. This raises the question of how widespread this type of regulation is. Several of the 23 *Arabidopsis* ARFs (AtARF1, AtARF6, AtARF7, AtARF17, and AtARF19) have been reported to be proteasomally degraded (Salmon et al., 2008; Lakehal et al., 2019; Jing et al., 2022). Neither the degron nor the biological relevance of these degradations is entirely clear, but there is clearly potential for this degradation mechanism to be intimately connected to auxin response.

## METHODS

### Plant growth conditions

*M. polymorpha* male Takaragaike-1 (Tak-1) and female Takaragaike-2 (Tak-2) plants were used as the wild-type variety. For vegetative propagation, plants were grown on $^1/_2$ Gamborg B5 media plates in growth chambers with 40 μmol photons $m^{-2}$ $s^{-1}$ continuous white light at 22°C. For sexual reproduction, plants were grown on $^1/_2$ Gamborg B5 medium supplemented with 1% sucrose and maintained under 40 μmol photons $m^{-2}$ $s^{-1}$ continuous white fluorescent light for 1 month. Plants were then moved into 40 μmol photons $m^{-2}$ $s^{-1}$ continuous white light supplemented with 15 μmol photons $m^{-2}$ $s^{-1}$ far-red light to induce antheridiophore and archegoniophore development. Plants were repeatedly crossed manually to ensure fertilization. Sporangia with mature spores were collected aseptically and used in spore transformation.

### Development of genomic knockin translational fusions

*Marchantia* knockin lines were developed to study native auxin response proteins at endogenous concentrations. A nopaline synthase (NOS) terminator and a fluorescent marker gene (either mScarlet-I or mNeonGreen) were cloned sequentially at the HindIII restriction site of the pJHY-TMp1 binary plasmid to create pJHY-mScarlet-I and pJHY-mNeonGreen vectors. After each cloning step, the HindIII site was regenerated by adding a HindIII site in the forward primer. This enabled subsequent cloning at the 5′ end of the previous insert in the same plasmid. Two 3.5-kb genomic DNA fragments were amplified by PCR and used as homologous arms for recombination. The first genomic DNA fragment contained the 3.5-kb sequence upstream of the stop codon of the gene of interest, and the second fragment was composed of the 3.5-kb sequence downstream of the stop codon. The first fragment was cloned at the HindIII site, and the second fragment was cloned at the AscI site of pJHY-mScarlet-I and pJHY-mNeonGreen. This cloning strategy was used to create homologous recombination constructs for ARF1, ARF2, ARF3, TIR1, Aux/IAA, and ncARF. Wild-type (Tak-1) spores were transformed by the *Agrobacterium*-mediated transformation protocol described in Ishizaki et al. (2008). Transformants were selected on $^1/_2$ Gamborg B5 + 100 mg/L cefotaxime medium with 10 mg/L hygromycin selection. Genomic DNA PCR was used to isolate true knockin lines.

### Generation of *MpARF2* overexpression lines

To overexpress *MpARF2* in *Marchantia*, the *MpARF2* coding sequence was amplified and cloned under the *MpEF1* promoter. The *MpARF2*-CDS was cloned into the published gateway vectors pMpGWB103 and pMpGWB108 (Ishizaki et al., 2015) to generate an unfused and a citrine-fluorophore-fused version, respectively. Constructs were transformed in *Marchantia* Tak-1 using *Agrobacterium*-mediated transformation of gemmae. Positive transformants were obtained through hygromycin selection on $^1/_2$ Gamborg B5 medium plates.

### Auxin sensitivity and physiological analysis of knockins

Knockin lines were tested for their wild-type-like growth, physiology, and auxin sensitivity. Tak-1, Tak-2, and all knockin lines were treated with $^1/_2$ B5 medium supplemented with either DMSO or 3 μM 1-NAA and grown for 7 days. On the 8th day, plants ($n$ = 10 per genotype) were imaged with a stereomicroscope to compare their physiological responses to auxin (Supplemental Figure S1).

### Microscope slide-mount setup for time-course imaging

A microscope slide mount was set up for live imaging of gemmae to precisely track a selected set of cells for temporal protein expression analysis. The mount consisted of a circular aluminum disc with a plastic inset fitted at the center of the disc (Supplemental Figure S11). Melted $^1/_2$ B5 medium with or without desired treatments was poured into the cavity of the plastic inset and allowed to solidify. Gemmae were carefully placed on top of the solidified medium and covered with a round coverslip. The bottom of the mount was sealed with parafilm to prevent any evaporation and drying of the medium during time-series imaging. Between two imaging time points in a time-series experiment, the mounts were placed inverted in growth chambers to keep the gemmae exposed to light and allow for normal growth.

### Confocal live-cell imaging

All live-cell imaging was performed on a Leica SP8X-SMD confocal microscope equipped with hybrid detectors and a pulsed (40 MHz) white-light laser. mNeonGeen and mScarlet-I were excited with the 506-nm and 561-nm laser lines, respectively. The laser powers were set at 4% output to avoid bleaching of the fluorophores. Fluorescence was detected between 512 and 560 nm (mNeonGreen) and 570 and 620 nm (mScarlet-I) using hybrid detectors in photon counting mode. Z-stack images were acquired using a 20× water immersion objective lens and time-gated detection to suppress chlorophyll autofluorescence. Images were processed using ImageJ software. Maximum-intensity projections of z-stack images were used to quantify total cellular fluorescence in each analyzed nucleus, corrected for background fluorescence. For presentation of the representative images of protein expression patterns, outlines of gemmae were cut in Adobe Photoshop and placed onto a black background. For quantification of images, the background signal was always taken into consideration.

### RNA extraction, cDNA synthesis, and qPCR

Total plant RNA was extracted from gemmae collected from gemma cups of 4-week-old Tak-1 and knockin plants and subsequently incubated in liquid $^1/_2$ B5 medium for 0, 3.5, and 8 h before freezing in liquid nitrogen. RNA was extracted from ground tissue using the TRIzol reagent and the Qiagen Plant mini-kit. An on-column RNase-free DNase (Qiagen) treatment was performed before final elution. cDNA was synthesized from 1 μg total RNA using the iScript Reverse Transcriptase kit (Bio-Rad). qPCR reactions were carried out using 2× IQ SYBR green (Bio-Rad) on a CFX384 Touch Real-Time PCR detection system (Bio-Rad). Data analysis was performed as described in Taylor et al. (2019). The housekeeping genes *MpEF1a* and *MpSAND* were used for transcript-level normalization.

### RNA-seq data analysis

The quality of the raw fastq reads was analyzed in FastQC. Reads were then mapped onto the *M. polymorpha* genome (MpTak1v5.1, accessed through MarpolBase, https://marchantia.info/download/tak1v5.1/) using HISAT2 version 2.2.1 (Kim et al., 2019) with -dta and -trim5 10 as additional parameters. SAM and BAM files were handled using SAMtools version 1.11 (Danecek et al., 2021). The raw reads were then counted using featureCounts version 2.0.1 (Liao et al., 2014) with the additional parameters: -t exon; -g gene_id; -Q 30; -p; -primary. The results were imported into R version 3.6.1, and raw count normalization as well as identification of differentially expressed genes (adjusted *p value [*padj] <0.05) were performed using DESeq2 (Love et al., 2014). Genes with a total read count <45 were excluded from the analysis, and genes with an absolute $log_2$(FC) of >1 and padj < 0.05 were deemed differentially expressed. All plots were generated in R (www.r-project.org). Raw RNA-seq reads were deposited in the NCBI Sequence Read Archive under project number PRJNA1019931.

### Inducible ARF2 overexpression

For inducible MpARF2 overexpression, the pEF1MpARF2-GR lines were used (Kato et al., 2020). Plants ($n$ = 10 per genotype) were treated with either DMSO or 1 μM dexamethasone in B5 medium and imaged after 3 days to look for rhizoid formation as an indicator of gemma germination. Dexamethasone treatment was used to induce the movement of ARF2-GR from the cytosol to the nucleus.

### Mathematical modeling

Details of modeling are described in detail in Supplemental File 1.

### IAA quantification

Total IAA was quantified to estimate the total cellular auxin levels during gemma growth. Gemmae were collected from 4-week-old Tak-1 plants. Tak-1 gemmae were grown on liquid $^{1}/_{2}$ B5 medium, and samples were collected after 0, 3.5, 8, and 24 h of growth. Samples were snap frozen in liquid nitrogen, ground into a fine powder, and weighed. For extraction of IAA, ~150 mg snap-frozen plant material was used per sample. Tissue was ground into a fine powder at −80°C using 3-mm stainless-steel beads at 50 Hz for 2 × 30 s in a TissueLyser LT (Qiagen). Ground samples were extracted with 1 mL cold methanol containing [phenyl $^{13}C_6$]-IAA (0.1 nmol/mL) as an internal standard in a 2-mL Eppendorf tube as described previously (Ruyter-Spira et al., 2011). Samples were filtered through a 0.45-μm Minisart SRP4 filter (Sartorius) and measured on the same day. IAA was measured on a Waters Xevo TQ-S tandem quadrupole mass spectrometer.

## SUPPLEMENTAL INFORMATION

Supplemental information is available at *Plant Communications Online*.

## FUNDING

This work was supported by the Netherlands Organisation for Scientific Research, the Netherlands (grants ALWOP.402 and OCENW.M20.031 to J.W.B.) and the Human Frontiers Research Program (grant RGP0015/2022 to D.W.).

## AUTHOR CONTRIBUTIONS

Conceptualization, D.W. and J.W.B. Investigation, S.D., M.d.R., S.M., M.D.A., and W.K. Modeling, E.F. and S.B. Writing – original draft, S.D., M.d.R., W.K., S.B., E.F., J.W.B., and D.W. Writing – review & editing, S.D., J.W.B., and D.W. Supervision, J.W.B. and D.W.

## ACKNOWLEDGEMENTS

We are grateful to Iris Nieuwland and Neri van Laar for experimental support. No conflict of interest declared.

Received: October 19, 2023
Revised: July 3, 2024
Accepted: July 8, 2024
Published: July 9, 2024

## REFERENCES

**Ashton, N.W., Grimsley, N.H., and Cove, D.J.** (1979). Analysis of gametophytic development in the moss, Physcomitrella patens, using auxin and cytokinin resistant mutants. Planta **144**:427–435. https://doi.org/10.1007/BF00380118.

**Boer, D.R., Freire-Rios, A., van den Berg, W.A., Saaki, T., Manfield, I.W., Kepinski, S., Lopez-Vidrieo, I., Franco-Zorrilla, J.M., de Vries, S.C., Solano, R., et al.** (2014). Structural basis for DNA binding specificity by the auxin-dependent ARF transcription factors. Cell **156**:577–589. https://doi.org/10.1016/j.cell.2013.12.027.

**Danecek, P., Bonfield, J.K., Liddle, J., Marshall, J., Ohan, V., Pollard, M.O., Whitwham, A., Keane, T., McCarthy, S.A., Davies, R.M., et al.** (2021). Twelve years of SAMtools and BCFtools. Gigascience **10**. https://doi.org/10.1093/gigascience/giab008.

**Dreher, K.A., Brown, J., Saw, R.E., and Callis, J.** (2006). The Arabidopsis Aux/IAA protein family has diversified in degradation and auxin responsiveness. Plant Cell **18**:699–714. https://doi.org/10.1105/tpc.105.039172.

**Eklund, D.M., Ishizaki, K., Flores-Sandoval, E., Kikuchi, S., Takebayashi, Y., Tsukamoto, S., Hirakawa, Y., Nonomura, M., Kato, H., Kouno, M., et al.** (2015). Auxin Produced by the Indole-3-Pyruvic Acid Pathway Regulates Development and Gemmae Dormancy in the Liverwort Marchantia polymorpha. Plant Cell **27**:1650–1669. https://doi.org/10.1105/tpc.15.00065.

**Flores-Sandoval, E., Eklund, D.M., and Bowman, J.L.** (2015). A Simple Auxin Transcriptional Response System Regulates Multiple Morphogenetic Processes in the Liverwort Marchantia polymorpha. PLoS Genet. **11**:e1005207. https://doi.org/10.1371/journal.pgen.1005207.

**Flores-Sandoval, E., Eklund, D.M., Hong, S.F., Alvarez, J.P., Fisher, T.J., Lampugnani, E.R., Golz, J.F., Vazquez-Lobo, A., Dierschke, T., Lin, S.S., and Bowman, J.L.** (2018). Class C ARFs evolved before the origin of land plants and antagonize differentiation and developmental transitions in Marchantia polymorpha. New Phytol. **218**:1612–1630. https://doi.org/10.1111/nph.15090.

**Freire-Rios, A., Tanaka, K., Crespo, I., van der Wijk, E., Sizentsova, Y., Levitsky, V., Lindhoud, S., Fontana, M., Hohlbein, J., Boer, D.R., et al.** (2020). Architecture of DNA elements mediating ARF transcription factor binding and auxin-responsive gene expression in Arabidopsis. Proc. Natl. Acad. Sci. USA **117**:24557–24566. https://doi.org/10.1073/pnas.2009554117.

**Friml, J.** (2022). Fourteen Stations of Auxin. Cold Spring Harbor Perspect. Biol. **14**:a039859. https://doi.org/10.1101/cshperspect.a039859.

**Gray, W.M., Kepinski, S., Rouse, D., Leyser, O., and Estelle, M.** (2001). Auxin regulates SCF(TIR1)-dependent degradation of AUX/IAA proteins. Nature **414**:271–276. https://doi.org/10.1038/35104500.

**He, W., Brumos, J., Li, H., Ji, Y., Ke, M., Gong, X., Zeng, Q., Li, W., Zhang, X., An, F., et al.** (2011). A small-molecule screen identifies L-kynurenine as a competitive inhibitor of TAA1/TAR activity in ethylene-directed auxin biosynthesis and root growth in Arabidopsis. Plant Cell **23**:3944–3960. https://doi.org/10.1105/tpc.111.089029.

**Ishizaki, K., Chiyoda, S., Yamato, K.T., and Kohchi, T.** (2008). Agrobacterium-mediated transformation of the haploid liverwort Marchantia polymorpha L., an emerging model for plant biology. Plant Cell Physiol. **49**:1084–1091. https://doi.org/10.1093/pcp/pcn085.

**Ishizaki, K., Johzuka-Hisatomi, Y., Ishida, S., Iida, S., and Kohchi, T.** (2013). Homologous recombination-mediated gene targeting in the liverwort Marchantia polymorpha L. Sci. Rep. **3**:1532. https://doi.org/10.1038/srep01532.

**Ishizaki, K., Nishihama, R., Ueda, M., Inoue, K., Ishida, S., Nishimura, Y., Shikanai, T., and Kohchi, T.** (2015). Development of Gateway Binary Vector Series with Four Different Selection Markers for the Liverwort Marchantia polymorpha. PLoS One **10**:e0138876. https://doi.org/10.1371/journal.pone.0138876.

**Jing, H., Korasick, D.A., Emenecker, R.J., Morffy, N., Wilkinson, E.G., Powers, S.K., and Strader, L.C.** (2022). Regulation of AUXIN RESPONSE FACTOR condensation and nucleo-cytoplasmic partitioning. Nat. Commun. **13**:4015. https://doi.org/10.1038/s41467-022-31628-2.

**Jones, V.A., and Dolan, L.** (2012). The evolution of root hairs and rhizoids. Ann. Bot. **110**:205–212. https://doi.org/10.1093/aob/mcs136.

**Kato, H., Ishizaki, K., Kouno, M., Shirakawa, M., Bowman, J.L., Nishihama, R., and Kohchi, T.** (2015). Auxin-Mediated Transcriptional System with a Minimal Set of Components Is Critical for Morphogenesis through the Life Cycle in Marchantia polymorpha. PLoS Genet. **11**:e1005084. https://doi.org/10.1371/journal.pgen.1005084.

**Kato, H., Kouno, M., Takeda, M., Suzuki, H., Ishizaki, K., Nishihama, R., and Kohchi, T.** (2017). The Roles of the Sole Activator-Type Auxin Response Factor in Pattern Formation of Marchantia polymorpha. Plant Cell Physiol. **58**:1642–1651. https://doi.org/10.1093/pcp/pcx095.

**Kato, H., Mutte, S.K., Suzuki, H., Crespo, I., Das, S., Radoeva, T., Fontana, M., Yoshitake, Y., Hainiwa, E., van den Berg, W., et al.** (2020). Design principles of a minimal auxin response system. Nat. Plants **6**:473–482. https://doi.org/10.1038/s41477-020-0662-y.

**Kim, D., Paggi, J.M., Park, C., Bennett, C., and Salzberg, S.L.** (2019). Graph-based genome alignment and genotyping with HISAT2 and HISAT-genotype. Nat. Biotechnol. **37**:907–915. https://doi.org/10.1038/s41587-019-0201-4.

**Lakehal, A., Chaabouni, S., Cavel, E., Le Hir, R., Ranjan, A., Raneshan, Z., Novak, O., Pacurar, D.I., Perrone, I., Jobert, F., et al.** (2019). A Molecular Framework for the Control of Adventitious Rooting by TIR1/AFB2-Aux/IAA-Dependent Auxin Signaling in Arabidopsis. Mol. Plant **12**:1499–1514. https://doi.org/10.1016/j.molp.2019.09.001.

**Lavy, M., and Estelle, M.** (2016). Mechanisms of auxin signaling. Development **143**:3226–3229. https://doi.org/10.1242/dev.131870.

**Lavy, M., Prigge, M.J., Tao, S., Shain, S., Kuo, A., Kirchsteiger, K., and Estelle, M.** (2016). Constitutive auxin response in Physcomitrella reveals complex interactions between Aux/IAA and ARF proteins. Elife **5**. https://doi.org/10.7554/eLife.13325.

**Leyser, O.** (2018). Auxin Signaling. Plant Physiol. **176**:465–479. https://doi.org/10.1104/pp.17.00765.

**Liao, Y., Smyth, G.K., and Shi, W.** (2014). featureCounts: an efficient general purpose program for assigning sequence reads to genomic features. Bioinformatics **30**:923–930. https://doi.org/10.1093/bioinformatics/btt656.

**Love, M.I., Huber, W., and Anders, S.** (2014). Moderated estimation of fold change and dispersion for RNA-seq data with DESeq2. Genome Biol. **15**:550. https://doi.org/10.1186/s13059-014-0550-8.

**Luo, J., Zhou, J.J., and Zhang, J.Z.** (2018). Aux/IAA Gene Family in Plants: Molecular Structure, Regulation, and Function. Int. J. Mol. Sci. **19**. https://doi.org/10.3390/ijms19010259.

**Mutte, S.K., Kato, H., Rothfels, C., Melkonian, M., Wong, G.K., and Weijers, D.** (2018). Origin and evolution of the nuclear auxin response system. Elife **7**. https://doi.org/10.7554/eLife.33399.

**Nishimura, T., Hayashi, K., Suzuki, H., Gyohda, A., Takaoka, C., Sakaguchi, Y., Matsumoto, S., Kasahara, H., Sakai, T., Kato, J., et al.** (2014). Yucasin is a potent inhibitor of YUCCA, a key enzyme in auxin biosynthesis. Plant J. **77**:352–366. https://doi.org/10.1111/tpj.12399.

**Prigge, M.J., Lavy, M., Ashton, N.W., and Estelle, M.** (2010). Physcomitrella patens auxin-resistant mutants affect conserved elements of an auxin-signaling pathway. Curr. Biol. **20**:1907–1912. https://doi.org/10.1016/j.cub.2010.08.050.

**Roberts, A.W., Dimos, C.S., Budziszek, M.J., Jr., Goss, C.A., and Lai, V.** (2011). Knocking out the wall: protocols for gene targeting in Physcomitrella patens. Methods Mol. Biol. **715**:273–290. https://doi.org/10.1007/978-1-61779-008-9_19.

**Ruyter-Spira, C., Kohlen, W., Charnikhova, T., van Zeijl, A., van Bezouwen, L., de Ruijter, N., Cardoso, C., Lopez-Raez, J.A., Matusova, R., Bours, R., et al.** (2011). Physiological effects of the synthetic strigolactone analog GR24 on root system architecture in Arabidopsis: another belowground role for strigolactones? Plant Physiol. **155**:721–734. https://doi.org/10.1104/pp.110.166645.

**Sakakibara, K., Nishiyama, T., Sumikawa, N., Kofuji, R., Murata, T., and Hasebe, M.** (2003). Involvement of auxin and a homeodomain-leucine zipper I gene in rhizoid development of the moss Physcomitrella patens. Development **130**:4835–4846. https://doi.org/10.1242/dev.00644.

**Salmon, J., Ramos, J., and Callis, J.** (2008). Degradation of the auxin response factor ARF1. Plant J. **54**:118–128. https://doi.org/10.1111/j.1365-313X.2007.03396.x.

**Suzuki, H., Kato, H., Iwano, M., Nishihama, R., and Kohchi, T.** (2023). Auxin signaling is essential for organogenesis but not for cell survival in the liverwort Marchantia polymorpha. Plant Cell **35**:1058–1075. https://doi.org/10.1093/plcell/koac367.

**Tan, X., Calderon-Villalobos, L.I., Sharon, M., Zheng, C., Robinson, C.V., Estelle, M., and Zheng, N.** (2007). Mechanism of auxin perception by the TIR1 ubiquitin ligase. Nature **446**:640–645. https://doi.org/10.1038/nature05731.

**Taylor, S.C., Nadeau, K., Abbasi, M., Lachance, C., Nguyen, M., and Fenrich, J.** (2019). The Ultimate qPCR Experiment: Producing Publication Quality, Reproducible Data the First Time. Trends Biotechnol. **37**:761–774. https://doi.org/10.1016/j.tibtech.2018.12.002.

**Teale, W.D., Paponov, I.A., and Palme, K.** (2006). Auxin in action: signalling, transport and the control of plant growth and development. Nat. Rev. Mol. Cell Biol. **7**:847–859. https://doi.org/10.1038/nrm2020.

**Vanneste, S., and Friml, J.** (2009). Auxin: a trigger for change in plant development. Cell **136**:1005–1016. https://doi.org/10.1016/j.cell.2009.03.001.

**Weijers, D., and Wagner, D.** (2016). Transcriptional Responses to the Auxin Hormone. Annu. Rev. Plant Biol. **67**:539–574. https://doi.org/10.1146/annurev-arplant-043015-112122.

**Xia, R., Xu, J., and Meyers, B.C.** (2017). The Emergence, Evolution, and Diversification of the miR390-TAS3-ARF Pathway in Land Plants. Plant Cell **29**:1232–1247. https://doi.org/10.1105/tpc.17.00185.

Plant Communications, Volume *5*

## Supplemental information

# Quantitative imaging reveals the role of MpARF proteasomal degradation during gemma germination

**Shubhajit Das, Martijn de Roij, Simon Bellows, Melissa Dipp Alvarez, Sumanth Mutte, Wouter Kohlen, Etienne Farcot, Dolf Weijers, and Jan Willem Borst**

**Supplemental information**

# Quantitative imaging reveals the role of MpARF proteasomal degradation during gemma germination

Shubhajit Das[1,4], Martijn de Roij[1], Simon Bellows[2], Melissa Dipp Alvarez[1], Sumanth Mutte[1], Wouter Kohlen[3], Etienne Farcot[2], Dolf Weijers[1*], Jan Willem Borst[1*]

[1]Laboratory of Biochemistry, Wageningen University and Research, the Netherlands.

[2]School of Mathematical Sciences, University of Nottingham, United Kingdom.

[3]Laboratory of Molecular Biology, Wageningen University and Research, the Netherlands.

[4]Current address: Institute of Science and Technology, Austria

*Corresponding authors dolf.weijers@wur.nl and janwillem.borst@wur.nl

## Supplemental Figures

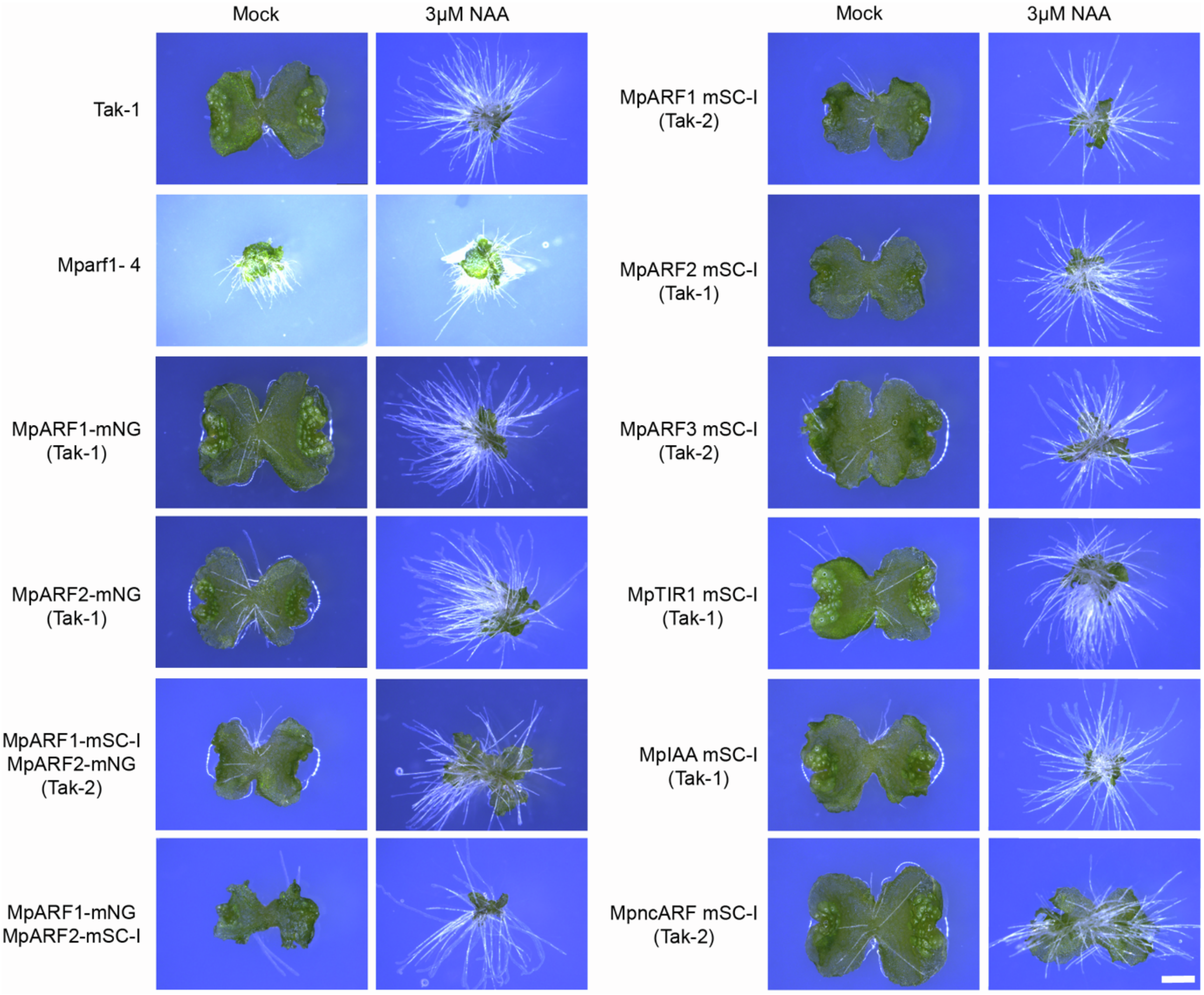


**Supplemental Figure S1: Phenotype and auxin response in knock-in lines.**
Auxin response assay on all genomic knock-in lines. All knock-ins were treated with mock or 3μM NAA and imaged after 1 week. All knock-ins show wild-type-like auxin response with thallus growth inhibition and ectopic rhizoid formation. Scale bar = 1 mm.

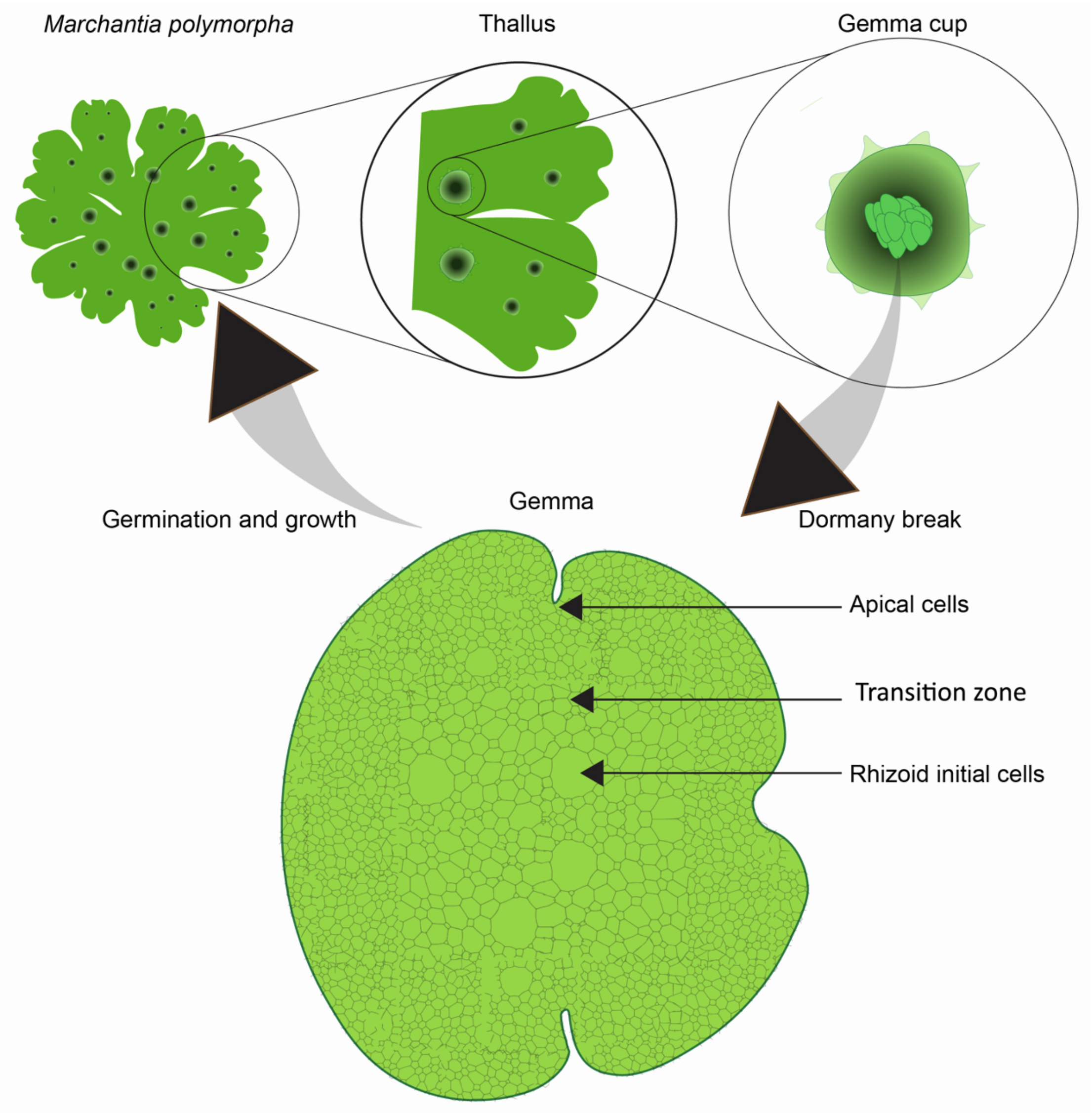


**Supplemental Figure S2.** Schematic representation of the Marchantia tissues and different cell types within a gemma.

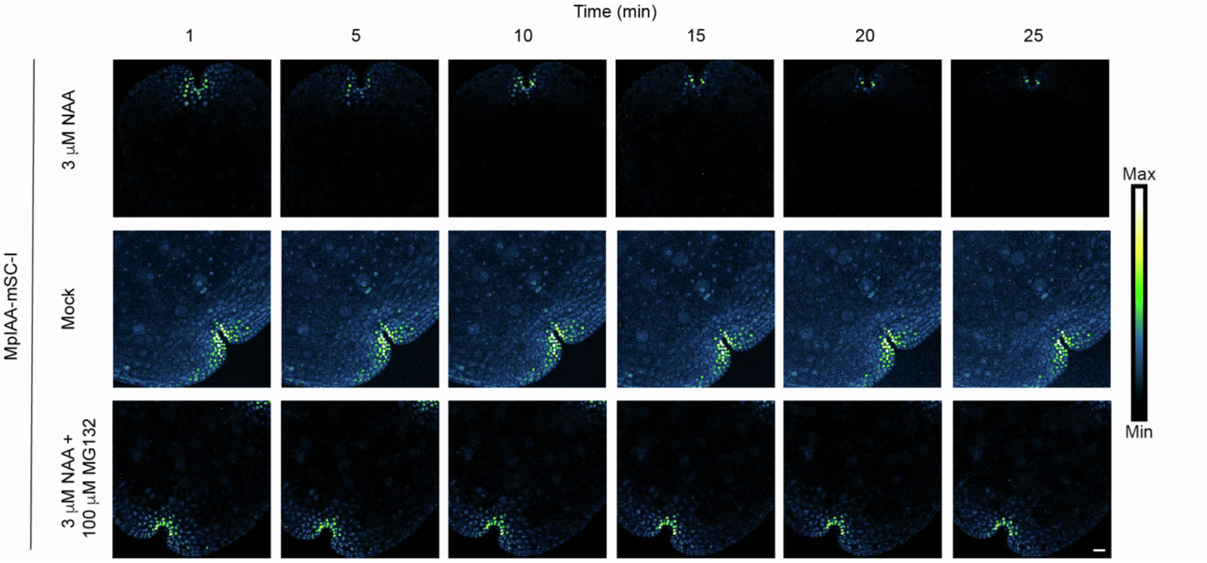


**Supplemental Figure S3**: Time-lapse imaging on MpIAA-mSC-I gemmae pre-treated with (50 μM)L-Kynurenine and (50 μM) Yucasin. Upon auxin (1-NAA; 3μM) treatment, fluorescence rapidly decreases due to MpIAA-mSC-I degradation, whereas the fluorescence remains constant if the sample is mock treated or incubated with the proteasomal inhibitor MG132. Scale bar = 25 μm.

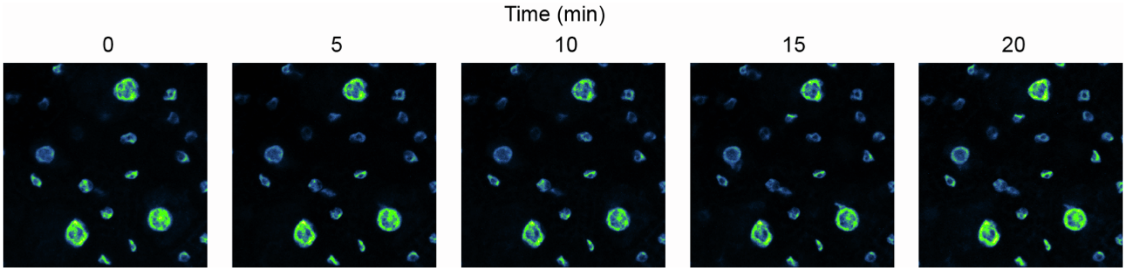


**Supplemental Figure S4**; time course of MpARF1-mSC-I at same time of MpIAA experiment showing no significant photobleaching of MpARF1-mSC-I.

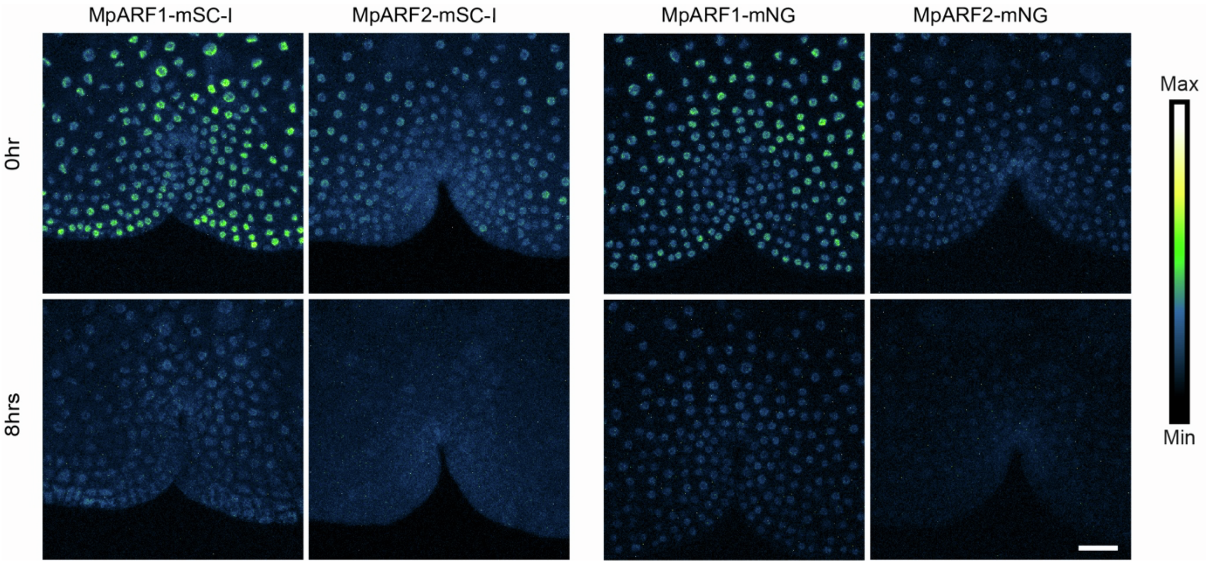


**Supplemental Figure S5. MpARF1 and MpARF2 protein accumulation dynamics is independent of the fluorophore tag used.**
Time course imaging on MpARF1 and MpARF2 single knock-in lines fused to mScarlet-I (mSC-I) and mNeonGreen (mNG) fluorescent proteins. Both mNG and mSC-I fusion variants of MpARF1 and MpARF2 show similar fluorescence decline during gemma germination. Scale bar = 50 µm.

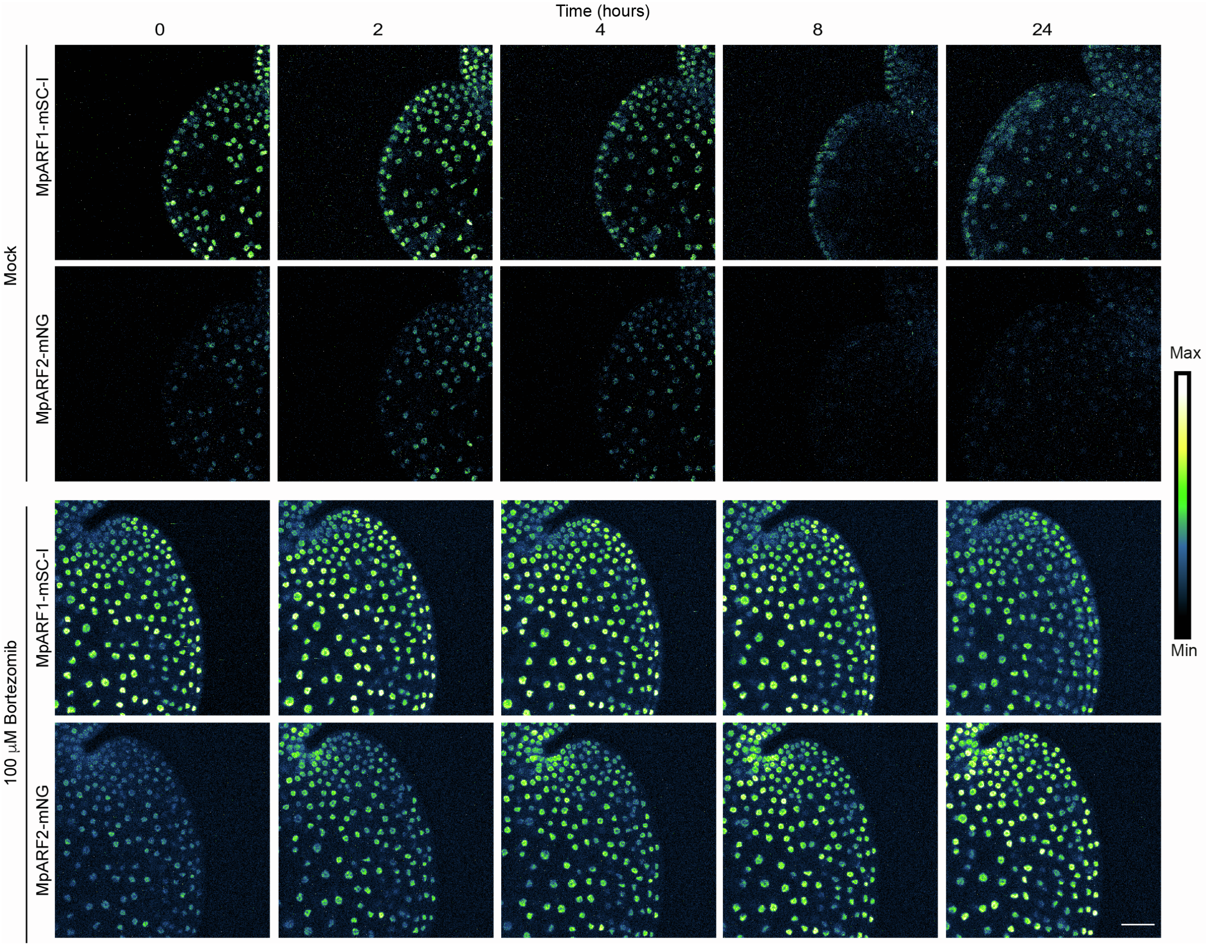


**Supplemental Figure S6. Bortezomib prevents MpARF degradation.**
Treatment with proteasomal degradation inhibitor Bortezomib also blocks the degradation of MpARF1-mSC-I and MpARF2-mNG. Scale bar = 50 µm

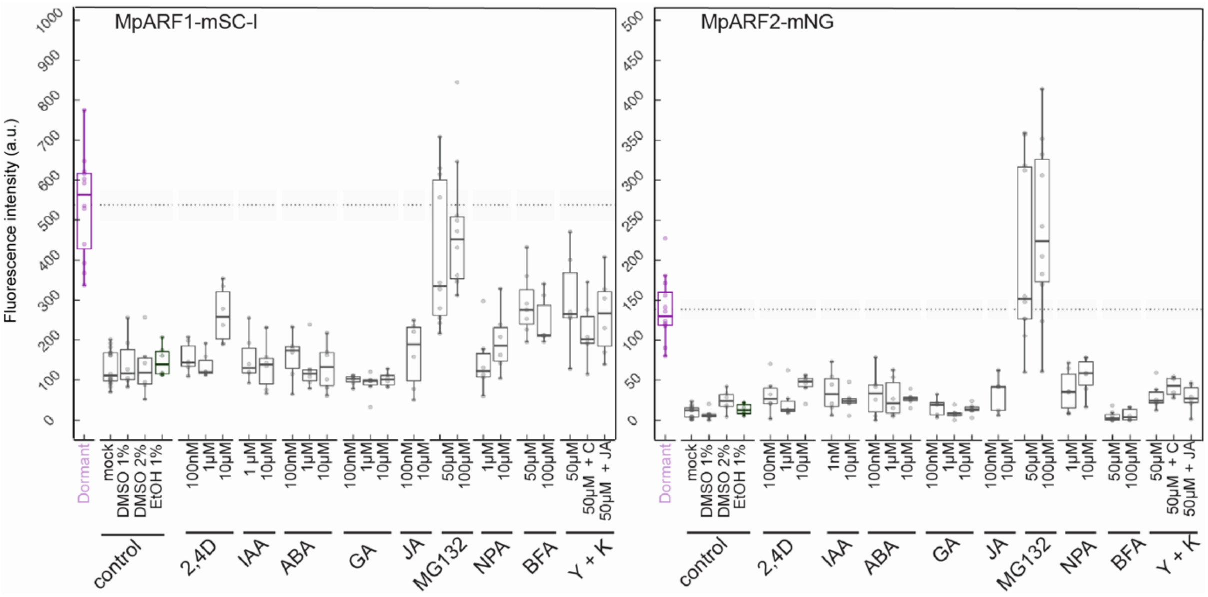


**Supplemental Figure S7. Proteasomal degradation of MpARF is independent of auxin and other tested common plant hormones.**

Treatment with common plant hormones such as natural (IAA) and synthetic (2,4-D) auxin, abscisic acid (ABA), gibberellic acid (GA), and jasmonic acid (JA), as well as inhibitors of auxin biosynthesis (Yucasin and Kyneurenine), auxin transport (NPA) and endocytosis(BFA), do not have any effect on MpARF degradation. Only proteasomal inhibitor (MG132) can block MpARF degradation.

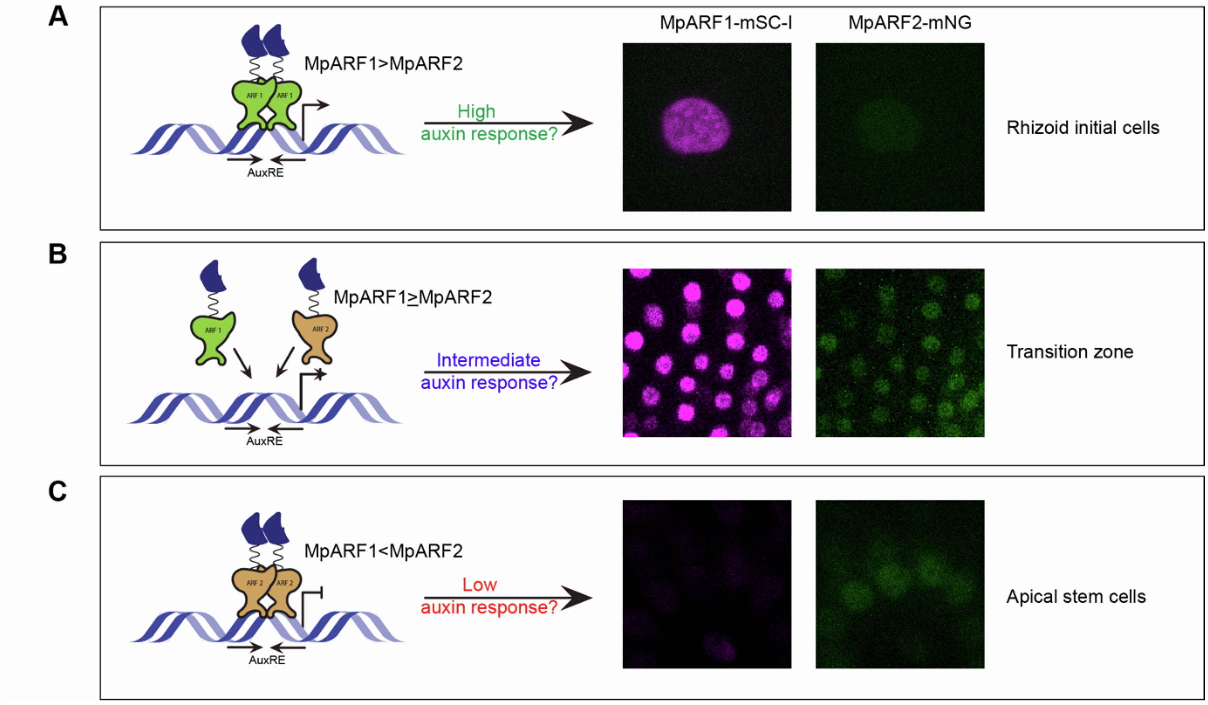


**Supplemental Figure S8. Model for how differential MpARF stoichiometry may underpin auxin response output across tissues.**

Three possible modes of gene regulation by the relative stoichiometry of class A and class B ARFs, adapted from Kato et. al. 2020. **A)** At a relatively higher concentration of MpARF1, DNA binding and gene activation is controlled by MpARF1 in an auxin-dependent manner. This mode might be operational in rhizoid initial cells where MpARF2 expression is below detection limit and MpARF1 is abundant. **B)** The second mode of gene regulation considers the presence of both class A and B MpARFs leading to competition-driven gene regulation(in the transition zone. **C)** The third mode describes gene repression by class B MpARFs, independent of cellular auxin concentrations. Apical meristem cells of gemmae could represent this third mode as these cells have relatively higher MpARF2 expression and no MpARF1 expression.

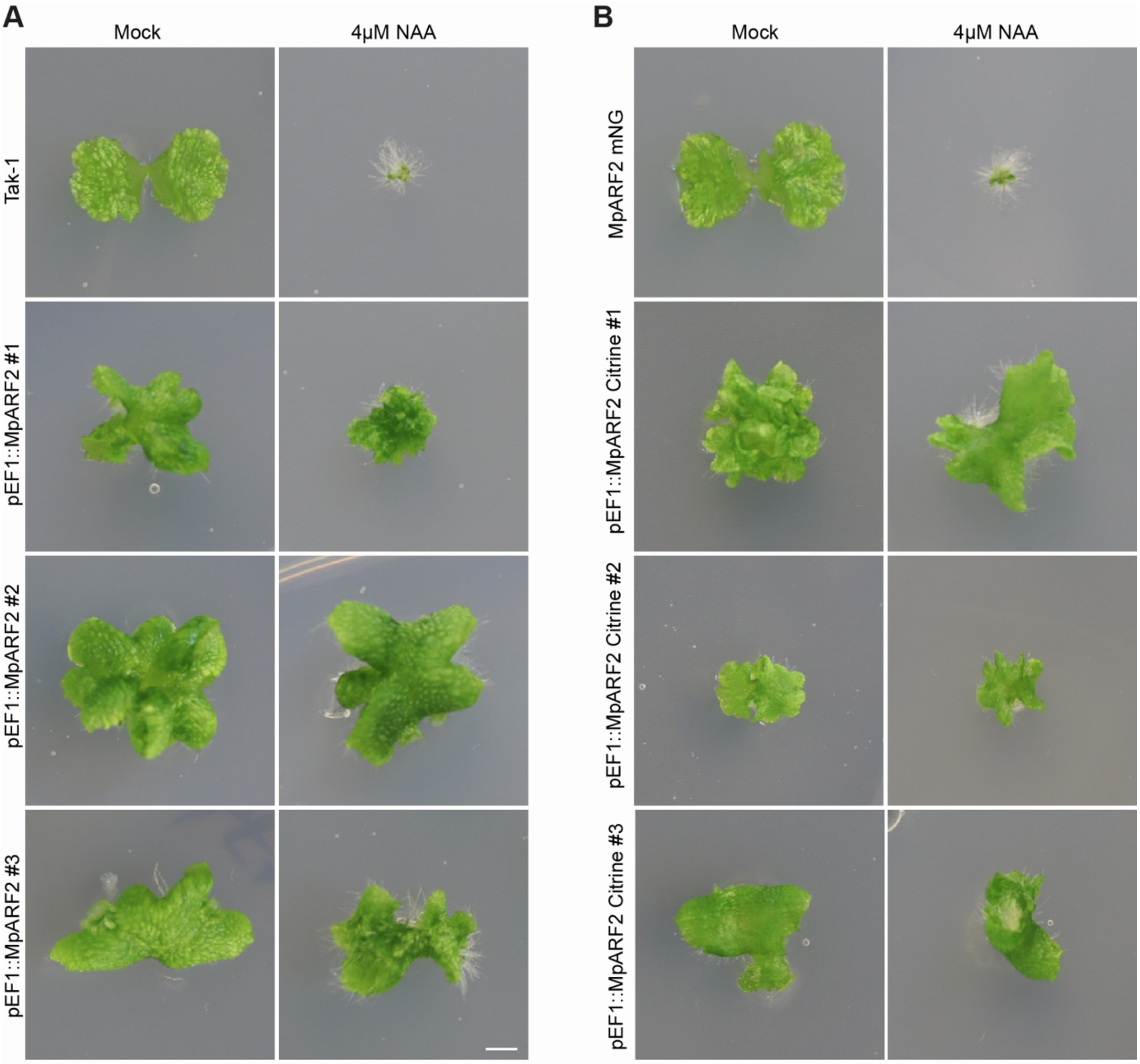


**Supplemental Figure S9. Accumulation of MpARF2 leads to an auxin insensitive phenotype.**
**A,B)** Growth of three independent pEF1::MpARF2 (**A**) and pEF1::MpARF2-Citrine (**B**) lines and Tak-1 control on control medium or medium containing 4 µM NAA. **C)** Expression of MpARF2-Citrine in pEF1::MpARF2-Citrine line#1. Scale bar = 25 µm.

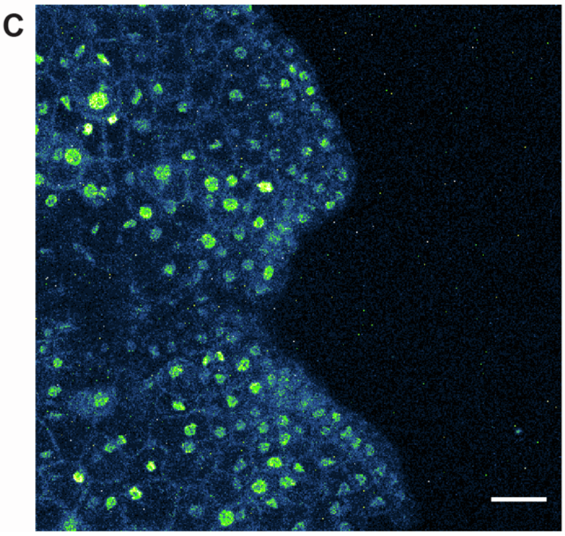

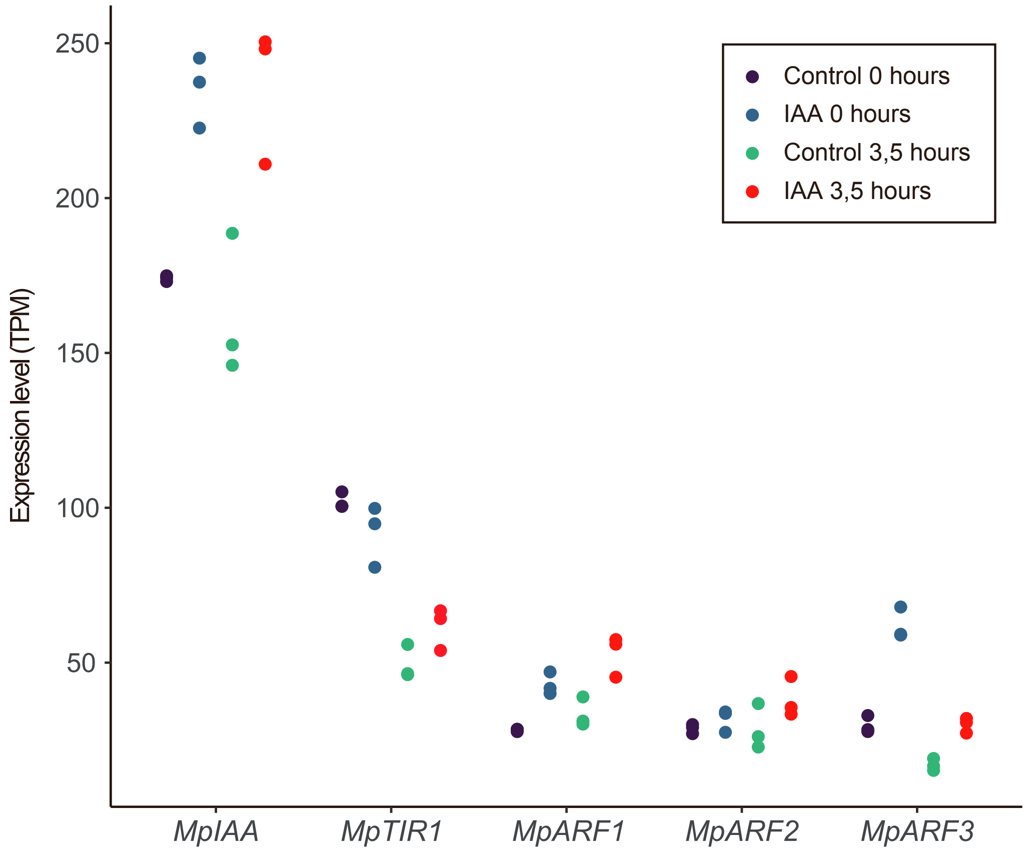


**Supplemental Figure S10:** Raw TPM expression values for MpIAA, MpTIR1 and MpARF1/2/3 in control- and IAA-treated gemmae at 0 and 3,5 hours after removal from the gemmae cup. Values of each replicate are shown.

**:**

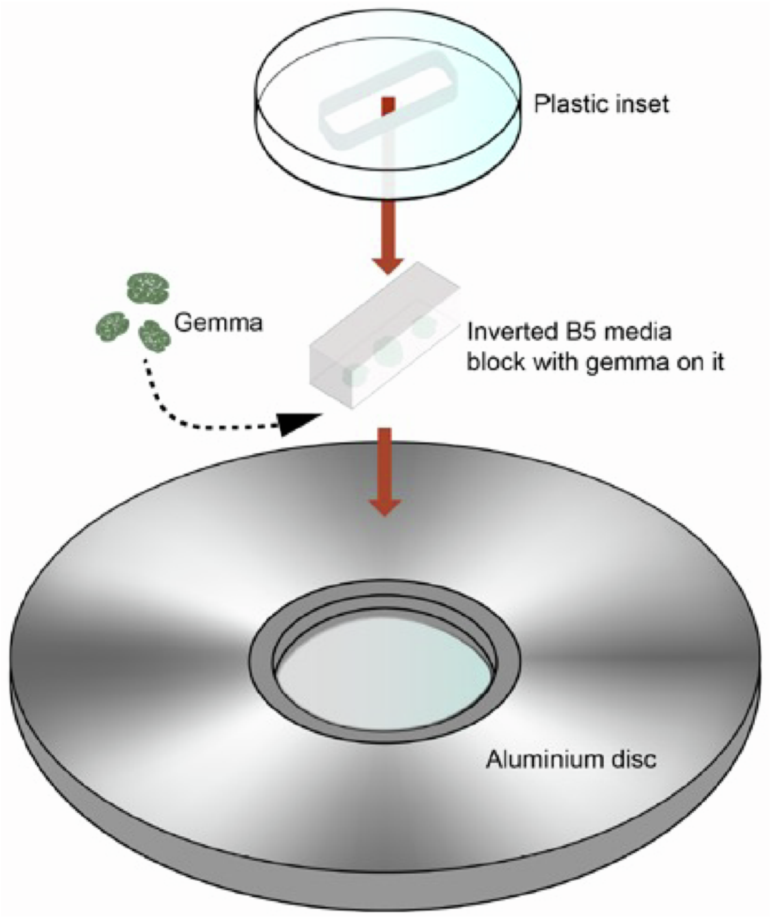


**Supplemental Figure S11: Microscope sample mount used to track protein accumulation patterns gemmae.**

Basic design of the microscope slide mount, used for time course imaging. B5 media blocks are solidified inside the plastic inset and gemmae are placed on top of the media. The media is supplemented with desired treatment or mock before casting in the inset. A round coverslip is gently placed on top of the gemmae. The plastic inset containing the gemma samples on the media block, is inverted and placed on the aluminium disc and tightened with a screw to prevent movement. Evaporation of water from the media block is prevented by sealing the reverse side of the block with parafilm.

**Supplemental File 1**

**Mathematical model**

In this supplement, a full description of the auxin signaling model for *Marchantia polymorpha* (Mp), used in the main paper, is provided. Note that for simplicity, the prefix Mp is typically dropped in the following as the model is largely species independent and relies on the general structure of the nuclear auxin pathway (NAP), with a few ad hoc assumptions. Firstly, the model is described, then some theoretical properties are discussed before showing some numerical simulations.

**Model description**

The main underlying assumptions are similar to previous NAP models from literature (Farcot et al., 2015), with adaptations motivated by the Marchantia data presented in the main paper. It is known that oligomers involving both ARF and Aux/IAA proteins can form via both a protein-protein domain, DIII-IV (or PB1 since its structure was uncovered) (Nanao et al., 2014) and a DNA binding domain, DBD (Boer et al., 2014). Based on this, the model includes populations of proteins and a generic AuxRE-bearing promoter, describing the competition between the different complexes that can be formed from these actors. As the model focuses on the early stages of development, at which Aux/IAA proteins are essentially absent from the system, we do not include Aux/IAA in the models. On the other hand, we consider both ARF1 and ARF2 along with all the complexes they can form, though limiting oligomer sizes to 2. This leads to the possible configurations - and transitions between them – shown in Supplemental Figure S12.

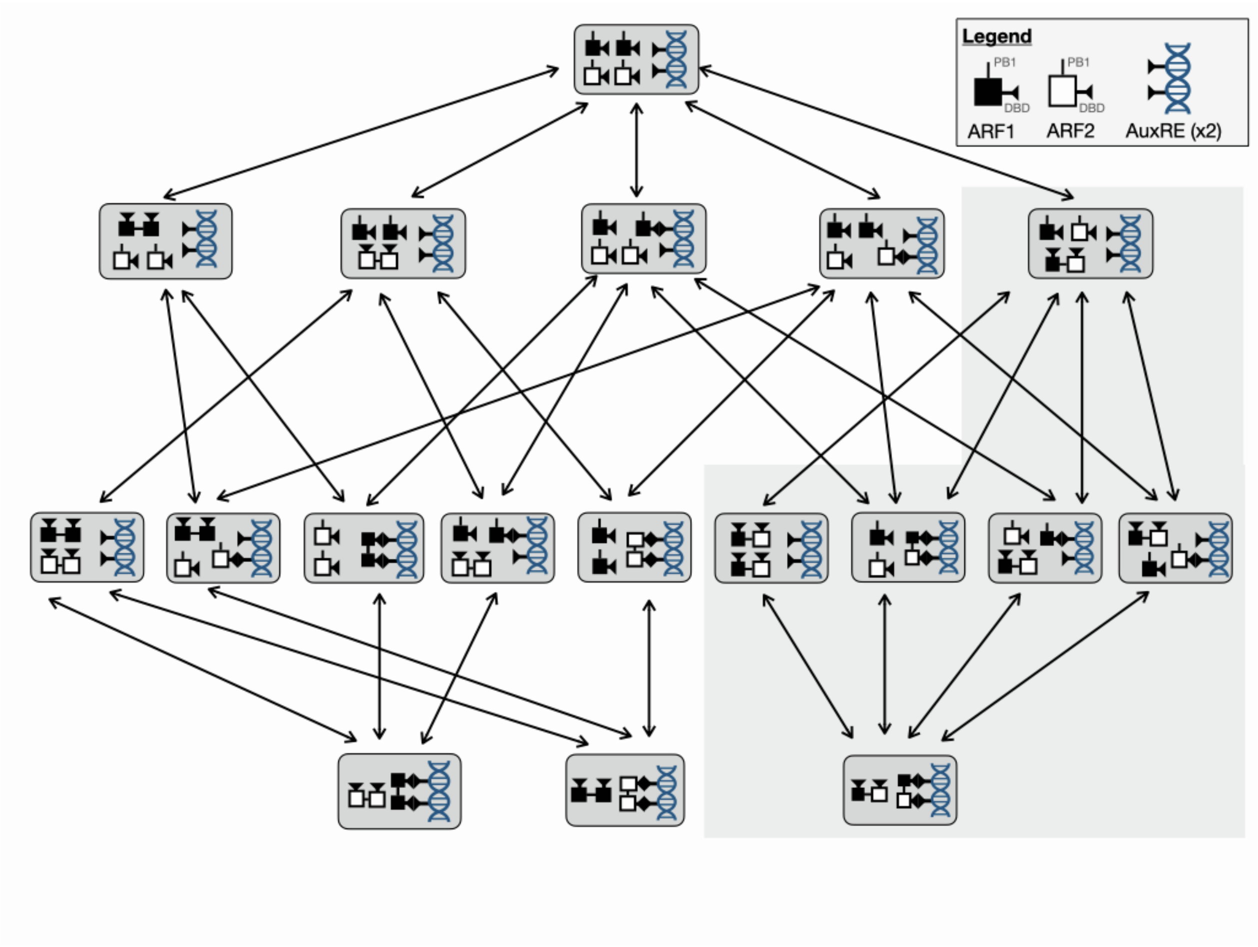


**Supplemental Figure S12:** All possible protein/AuxRE complexes included in the model. As one possible scenario, we have ignored all MpARF heterodimers, indicated by the grey box.

The equations are in an exact correspondence (using mass action) with the diagram in Supplemental Figure S12

$$\frac{dA_1}{dt} = \; - 2\alpha_{A_1A_1}A_1A_1 + 2\theta_{D_{A_1A_1}}D_{A_1A_1} - \alpha_{A_1A_2}A_1A_2 + \theta_{A_1A_2}D_{A_1A_2} - \alpha_{GA_1}GA_1 + \theta_{GA_1}G_{A_1}$$
$$-\alpha_{A_1GA_1}A_1G_{A_1}\theta_{A_1GA_1}G_{A_1A_1} - \alpha_{A_1GA_2}A_1G_{A_2} + \theta_{A_1GA_2}G_{A_1A_2}$$

$$\frac{dA_2}{dt} = -\alpha_{A_1A_2}A_1A_2 + \theta_{A_1A_2}D_{A_1A_2} - 2\alpha_{A_2A_2}A_2A_2 + 2\theta_{A_2A_2}D_{A_2A_2} - \alpha_{A_2GA_1}A_2G_{A_1} + \theta_{A_2GA_1}G_{A_1A_2}$$
$$-\alpha_{GA_2}GA_2 + \theta_{GA_2}G_{A_2} - \alpha_{A_2GA_2}G_{A_2}A_2 + \theta_{A_2GA_2}G_{A_2A_2}$$

$$\frac{dD_{A_1A_1}}{dt} = \alpha_{A_1A_1}A_1A_1 - \theta_{D_{A_1A_1}}D_{A_1A_1} - \alpha_{A_1A_1G}D_{A_1A_1}G + \theta_{A_1A_1G}G_{A_1A_1}$$

$$\frac{dD_{A_1A_2}}{dt} = \alpha_{A_1A_2}A_1A_2 - \theta_{A_1A_2}D_{A_1A_2} - \alpha_{A_1A_2G}D_{A_1A_2}G + \theta_{A_1A_2G}G_{A_1A_2}$$

$$\frac{dD_{A_2A_2}}{dt} = \alpha_{A_2A_2}A_2A_2 - \theta_{A_2A_2}D_{A_2A_2} + \theta_{A_2A_2G}G_{A_2A_2} - \alpha_{A_2A_2G}D_{A_2A_2}G$$

$$\frac{dG}{dt} = -\alpha_{GA_1}GA_1 + \theta_{GA_1}G_{A_1} - \alpha_{GA_2}GA_2 + \theta_{GA_2}G_{A_2} - \alpha_{A_1A_1G}D_{A_1A_1}G + \theta_{A_1A_1G}G_{A_1A_1}$$
$$-\alpha_{A_1A_2G}D_{A_1A_2}G + \theta_{A_1A_2G}G_{A_1A_2} - \alpha_{A_2A_2G}D_{A_2A_2}G + \theta_{A_2A_2G}G_{A_2A_2}$$

$$\frac{dG_{A_1}}{dt} = \alpha_{GA_1}GA_1 - \theta_{GA_1}G_{A_1} - \alpha_{A_1GA_1}A_1G_{A_1} + \theta_{A_1GA_1}G_{A_1A_1} - \alpha_{A_2GA_1}A_2G_{A_1} + \theta_{A_2GA_1}G_{A_1A_2}$$

$$\frac{dG_{A_2}}{dt} = \alpha_{GA_2}GA_2 - \theta_{GA_2}G_{A_2} - \alpha_{A_1GA_2}A_1G_{A_2} + \theta_{A_1GA_2}G_{A_1A_2} - \alpha_{A_2GA_2}G_{A_2}A_2 + \theta_{A_2GA_2}G_{A_2A_2}$$

$$\frac{dG_{A_1A_1}}{dt} = \alpha_{A_1GA_1}A_1G_{A_1} - \theta_{A_1GA_1}G_{A_1A_1} + \alpha_{A_1A_1G}D_{A_1A_1}G - \theta_{A_1A_1G}G_{A_1A_1}$$

$$\frac{dG_{A_1A_2}}{dt} = \alpha_{A_2GA_1}A_2G_{A_1} - \theta_{A_2GA_1}G_{A_1A_2} + \alpha_{A_1GA_2}A_1G_{A_2} - \theta_{A_1GA_2}G_{A_1A_2} + \alpha_{A_1A_2G}D_{A_1A_2}G - \theta_{A_1A_2G}G_{A_1A_2}$$

$$\frac{dG_{A_2A_2}}{dt} = \alpha_{A_2A_2G}D_{A_2A_2}G - \theta_{A_2A_2G}G_{A_2A_2} + \alpha_{A_2GA_2}G_{A_2}A_2 - \theta_{A_2GA_2}G_{A_2A_2}$$

The notational conventions are as follows:

- $A_1$ (resp. $A_2$) denotes the concentration of MpARF1 (resp. MpARF2).
- $D_{XY}$, $X, Y \in \{A_1, A_2\}$ denotes the concentration of an X:Y dimer, with parameters α, θ for association/dissociation rates as mentioned, and subscripts being hopefully explicit.
- $G$ , $G_{A_1}$, $G_{A_2}$, $G_{A_1A_1}$, $G_{A_1A_2}$, $G_{A_2A_2}$ denote the proportion, or probabilities, that a generic promoter is free, or bound with one of the possible ARF complexes as denoted in subscript.

To assign values to the constants $\alpha_{XY}$ and $\theta_{XY}$, we use estimates based on literature, in particular reference (Fontana et al., 2023). This paper shows that DBD allows for cooperative binding: dimers are more stably bound to DNA than monomers. To account for this, we use a cooperativity parameter $\gamma$, typically ≈100. In fact, data from (Fontana et al., 2023) indicates three orders of magnitude, for equilibrium dissociation constants:

$$K^d_{AA} \approx K^d_{AGA} \ll K^d_{AAG} \ll K^d_{AG},$$

denoting $K^d = \frac{\theta}{\alpha}$ and each occurrence of $A$ being either ARF1 or ARF2. It also appears that the intermediary constant is comparable to the dissociation rate of ARF:ARF dimers.
For simplicity, we rely on 4 pairs of association/dissociation constants:

- Kon1/Koff1 define ARF1 homodimerization; their default values are Kon1=1, Koff1=1.
- Kon2/Koff2 define ARF2 homodimerization; their default values are Kon2=1, Koff2=1.
- Kon3/Koff3 define ARF1/ARF2 heterodimerization, with defaults Kon3=1, Koff3=1.
- Kon4/Koff4 define ARF/AuxRE binding, supposed to be non-specific amongst ARFs; this defines the smallest order of magnitude above. Accordingly, their default values are Kon4=1, Koff4= 1/γ =0.01.

All parameters are listed below, following the conventions discussed above:

| Parameter | Value | Description |
|---|---|---|
| $\gamma$ | 100 | Cooperativity coefficient |
| $\alpha_{A_1A_1}$ / $\theta_{A_1A_1}$ | Kon1 / Koff1 | Association / dissociation of ARF1 with itself |
| $\alpha_{A_2A_2}$ / $\theta_{A_2A_2}$ | Kon2 / Koff2 | Association / dissociation of ARF2 with itself |
| $\alpha_{A_1A_2}$ / $\theta_{A_1A_2}$ | Kon3 / Koff3 | Association / dissociation of ARF1 and ARF2 |
| $\alpha_{A_iG} = \alpha_{A_iA_jG} = 2\alpha_{A_iGA_j}$ | Kon4 | Association of any ARF to a single AuxRE; $A_i, A_j$ denote any of ARF1/ARF2 |
| $\theta_{A_iG} = \gamma\theta_{A_iA_jG} = \gamma^2\theta_{A_iGA_j}$ | Koff4 | Dissociation of any ARF to a single AuxRE; $A_i, A_j$ denote any of ARF1/ARF2 |

**Model properties**
The structure of the model, see Figure S1, confers some general properties which are true regardless of specific parameters. Firstly, since the roles of the two ARFs are perfectly symmetric (whether heterodimers are included or not), one expects an exact balance between ARF1 (resp. ARF1:ARF1) and ARF2 (resp. ARF2:ARF2), unless there are quantitative differences in their binding/unbinding rates. However, even with similar kinetics the balance between Class A and Class B ARFs can be shifted by changes in the amount of available proteins, due to the presence of three conservation relations:

$$A_1^{tot} \doteq A_1 + 2A_{11} + A_{12} + G_{A_1} + 2G_{A_1A_1} + G_{A_1A_2}$$
$$A_2^{tot} \doteq A_2 + A_{12} + 2A_{22} + G_{A_2} + G_{A_1A_2} + 2G_{A_2A_2}$$
$$G^{tot} \doteq G + G_{A_1} + G_{A_2} + G_{A_1A_1} + G_{A_1A_2} + G_{A_2A_2}$$

Intuitively, these mean that the total amounts of each of ARF1, ARF2 and promoters remain unchanged over time. Therefore, they are completely determined by their initial value at time zero. The model describes how this initial amount is reallocated between the different subpopulations represented in Figure S1. This has two benefits:

- By systematically using initial conditions with $(G, G_{A_1}, G_{A_2}, G_{A_1A_1}, G_{A_1A_2}, G_{A_2A_2}) = (1,0,0,0,0,0)$, i.e. a population including only unoccupied promoters, the conservation relation guarantees that $G^{tot} = 1$ at all times, i.e. that the model describes a probability distribution.

- Since ARFs are conserved we can represent the experimental time series from the main paper as follows: for each experimental time point we use the experimental amounts of ARF1 and ARF2 as initial condition, from which we derive the distribution of all other variables using the model, which will therefore evolve between each time point. This explains why there is no production or degradation of ARFs included in the model equations. The underlying assumption is that the binding events described in the model are much faster than the 2h period between successive time points. This is justified given the dissociation constants shown e.g. (Han et al., 2014; Fontana et al., 2023), and which are of the order of 1-100 $s^{-1}$, giving time scales of minutes or shorter.

To describe the 'response' of the system, rather than using arbitrary assumptions to model a transcription rate, we consider the ratio between AuxRE sites occupied by activators and repressors:

$$Response = \frac{G_{A_1} + G_{A_1A_1} + G_{A_1A_2}}{G + G_{A_2} + G_{A_1A_2} + G_{A_2A_2}}$$

Note that whether $A_1A_2$ dimers are activators or repressors is unclear, so we include them twice. In fact, there is no clear evidence to date that these heterodimers form at all and in the following we will consider both scenarios with and without their formation.
One notices that the response term shown above is entirely auxin independent, which may seem contradictory. This is a consequence of these two facts:

(1) the above concerns a situation where Aux/IAA proteins have been removed.
(2) the primary effect of auxin on the NAP is to induce the degradation of Aux/IAA, hence suppressing their transcriptional repression as well as their sequestration of ARF proteins.

Consequently, in tissues where Aux/IAA have been degraded independently of auxin, it is expected that the effect of auxin itself becomes largely diminished. Including Aux/IAA explicitly in the model would result in a significant increase in complexity, roughly doubling the number of dimers seen in Supplemental Figure S12, and adding many unknown parameters. Given that Aux/IAA is experimentally mostly absent this seems an unnecessary complication.
One may still assess responsiveness to auxin as follows. Even a small amount of Aux/IAA will form dimers with ARF1, which will act as repressors. We denote auxin concentration by $x$ and the population of promoter bound ARF1:Aux/IAA dimers by $G_{A_1I}(x)$. The repressive effect of dimers means that the transcriptional response should include a new term:

$$R(x) = \frac{G_{A_1} + G_{A_1A_1} + G_{A_1A_2}}{G + G_{A_2} + G_{A_1A_2} + G_{A_2A_2} + G_{A_1I}(x)}$$

Since auxin induces Aux/IAA degradation, $G_{A_1I}(x)$ decreases to 0 as $x$ increases. One simple way to measure sensitivity is then to evaluate the rate of response change with respect to auxin:

$$\frac{dR}{dx}(x) = \frac{-(G_{A_1} + G_{A_1A_1} + G_{A_1A_2})}{\left(G + G_{A_2} + G_{A_1A_2} + G_{A_2A_2} + G_{A_1I}(x)\right)^2} \cdot \frac{dG_{A_1I}}{dx}(x)$$

As Aux/IAA is low, it makes sense to consider this rate when $G_{A_1 I}(x) \approx 0$. Also, the term $\frac{dG_{A_1 I}}{dx}(x) < 0$ is indicative of how effective the degradation of Aux/IAA is in response to auxin; with the data available this could only be a speculated figure. Therefore, we have chosen to measure auxin sensitivity as the rate of increase in transcription *relative to* the rate $\frac{dG_{A_1 I}}{dx}(x)$, in the limit of where $G_{A_1 I} \to 0$. In equation:

$$\text{(Auxin Sensitivity)} \qquad S = \frac{G_{A_1} + G_{A_1 A_1} + G_{A_1 A_2}}{\left(G + G_{A_2} + G_{A_1 A_2} + G_{A_2 A_2}\right)^2}$$

**Numerical simulations (time series)**

Time course simulations are run using the well-known ODE solver *scipy.integrate.odeint* available in Python 3.9, as well as plotting functions from the matplotlib library. All codes are available and free to use.

As mentioned previously, one uses the five timepoints t=0, 2, 4, 6, 8h present in the data. For each, we set an initial condition where ARF1 and ARF2 are directly read from the data, and all other variables are zero except for $G = 1$ (as discussed). We then solve the model using *odeint*, until nearing equilibrium. The obtained values are used for the corresponding timepoint. Successive timepoints are connected linearly, in absence of information regarding intermediary times. Using this method, the default parameter values lead to the time series shown in Figure S2.

As expected, the decrease of ARF monomers over time induces a redistribution of all other variables which results, overall in an increased transcriptional response, relative to a situation where ARF levels are maintained; with the intial ARFs amounts, there is ≈2.5x more activator-bound AuxREs than repressor-bound, and this raises to ≈5x as ARFs get degraded.

Using the model, different conditions can be simulated to assess their effect on the system's behavior. We include a small number here, but the python script could be used to investigate an endless number or alternative conditions. We restrict to two main aspects:

(1) As mentioned, there is no direct evidence that ARF1:ARF2 dimers form, only the fact that they cannot be ruled out based on protein structure only. We therefore ran simulations where Kon3=0, precluding the formation of heterodimers. Note that with our default assumptions on parameters, the routes $A_1 + A_2 + G \to \ G_{A_1} + A_2 \to G_{A_1 A_2}$ are still permitted since their rates are linked to Kon4. For this scenario specifically, we therefore forced Kon4 to 0, but only in terms leading to the formation $G_{A_1 A_2}$ , still allowing every other AuxRE configurations.

(2) Also mentioned earlier, the symmetry of the reaction network could be broken in favor of ARF1 accumulation if the rates Kon1/Koff1 are distinctly more favorable to dimer formation than the rates Kon2/Koff2. We therefore also considered this scenario, to assess how much this would enhance the response increase seen with default parameters (where all 4 rates are equal).

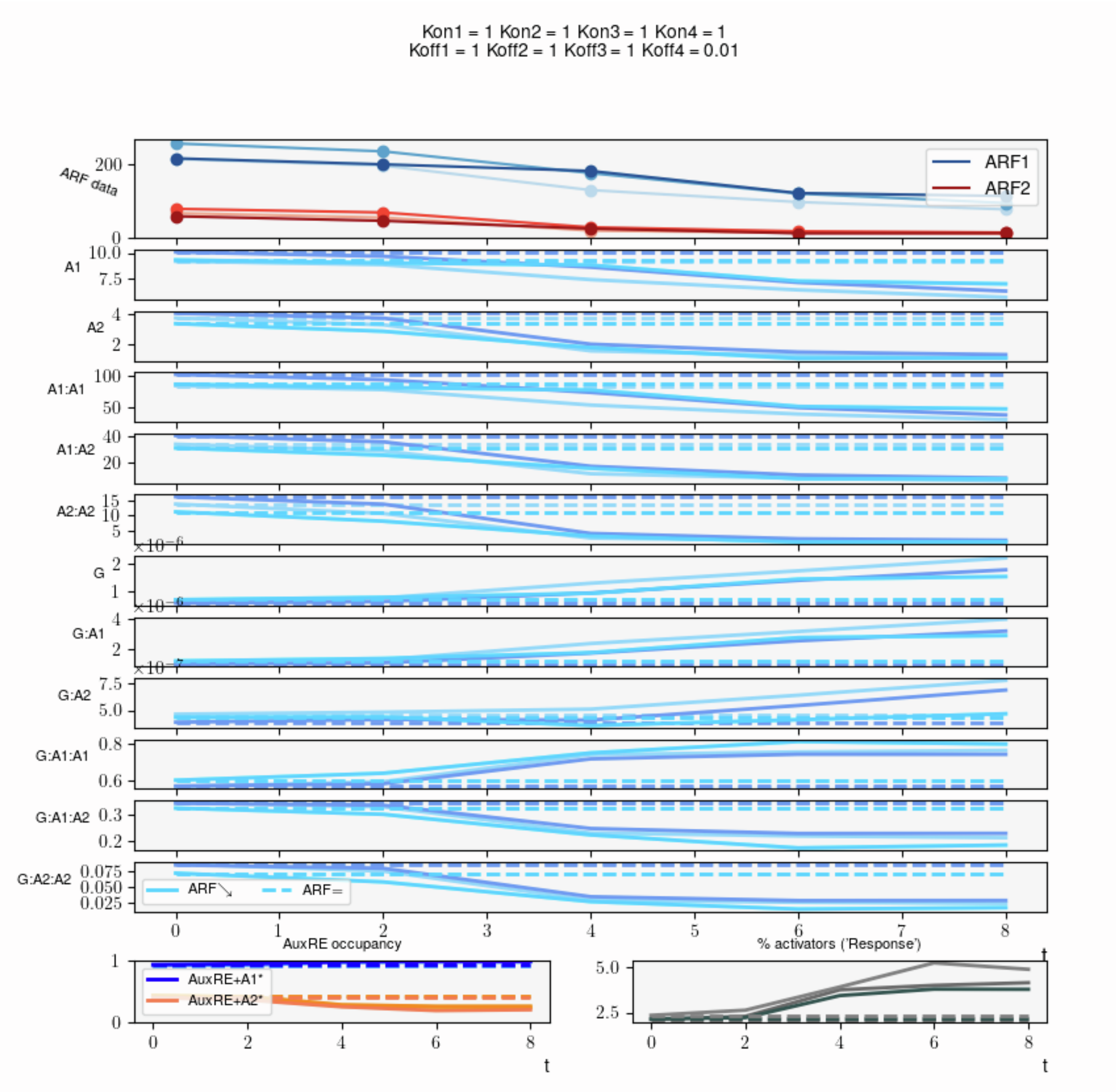


**Supplemental Figure S13:** Time simulation as described in the text, for the default parameter values. Experimental data is included in the first row. For comparison, variables as they would be without any decrease of ARF over time are shown using dashed lines. Shades of similar colors represent the three replicate time series obtained experimentally.

These alternative conditions are summarized in Supplemental Figure S14. Interestingly, there is little effect on the response resulting from an enhanced ARF1:ARF1 formation. This could be an indication that the repression of ARF seen in the data is a more effective mechanism to generate a transcriptional response. On the other hand, the removal of ARF1:ARF2 heterodimers (highlighted cases in Supplemental Figure S14) systematically leads to an improved response. This is due to the reduced competition between paths of dimer formation. It would be interesting to confirm experimentally whether heterodimers form.

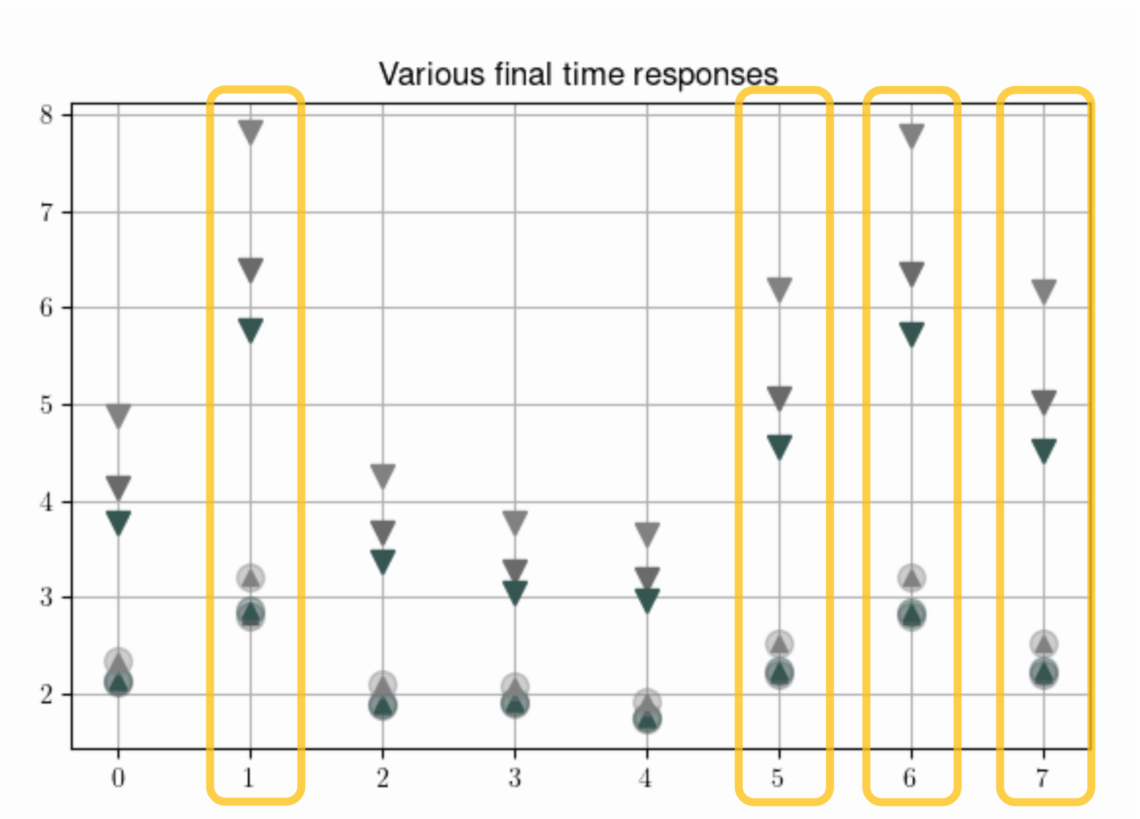


**Supplemental Figure S14:** One shade of grey for each of the 3-time courses. The final response value (downwards triangle) compared to its initial value (circle upwards triangle), for a range of conditions along the x-axis, specified as follows (only non-default parameter values are indicated):

| | | | |
|---|---|---|---|
| **0:** Default parameters (identical to Figure 2). | **1:** no A1:A2 heterodimers | **2:** Kon1=10. | **3:** Koff1=0.1 |
| **4:** Kon1=10, Koff1=0.1. | **5:** Kon1=10, no A1:A2 heterodimers | **6:** Koff1=0.1, no A1:A2 heterodimers | **7:** Kon1=10, Koff1=0.1, no A1:A2 heterodimers |

To conclude, we assessed sensitivity (as defined above) in a similar way to response amplitude. The results, shown in Supplemental Figure S15 are remarkably consistent with those pertaining to response amplitude; quantitative changes in the ARF1 affinities are largely inconsequential, whereas the removal of MpARF1:MpARF2 heterodimer significantly improves sensitivity to auxin.

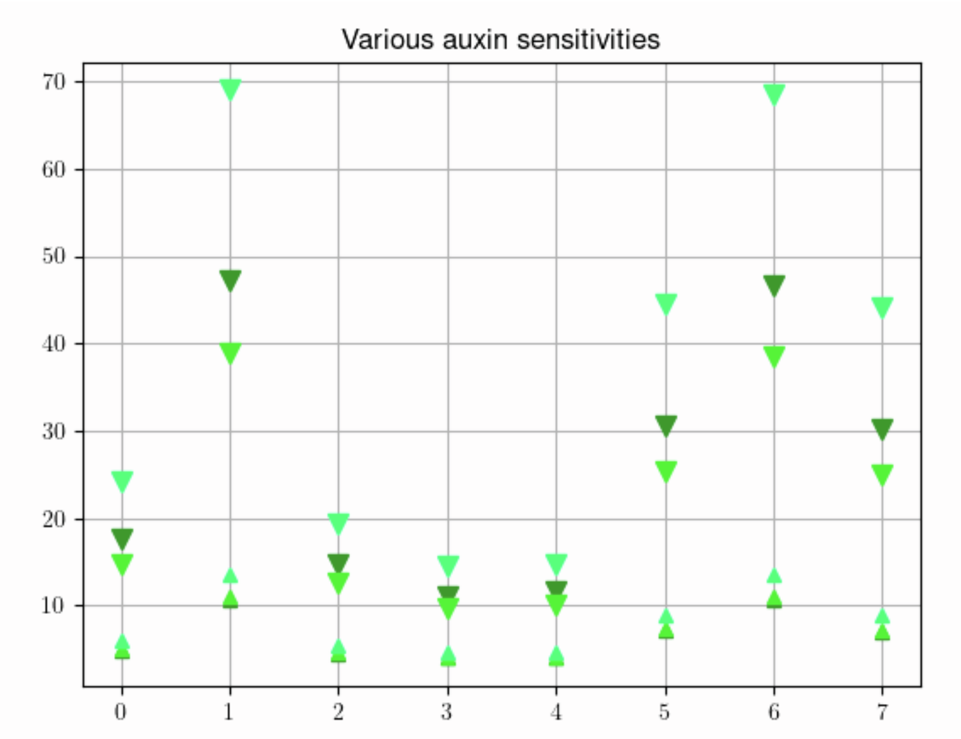


**Supplemental Figure S15:** One shade of green for each of the 3 time courses. The final sensitivity value (downwards triangle) compared to its initial value (upwards triangle), for a range of conditions along the x-axis, identically to Supplemental Figure S14.

## References


**Boer, D.R., Freire-Rios, A., van den Berg, W.A., Saaki, T., Manfield, I.W., Kepinski, S., Lopez-Vidrieo, I., Franco-Zorrilla, J.M., de Vries, S.C., Solano, R., Weijers, D., and Coll, M.** (2014). Structural basis for DNA binding specificity by the auxin-dependent ARF transcription factors. Cell **156,** 577-589.

**Farcot, E., Lavedrine, C., and Vernoux, T.** (2015). A modular analysis of the auxin signalling network. PloS one **10,** e0122231.

**Fontana, M., Roosjen, M., Crespo Garcia, I., van den Berg, W., Malfois, M., Boer, R., Weijers, D., and Hohlbein, J.** (2023). Cooperative action of separate interaction domains promotes high-affinity DNA binding of Arabidopsis thaliana ARF transcription factors. Proceedings of the National Academy of Sciences of the United States of America **120,** e2219916120.

**Han, M., Park, Y., Kim, I., Kim, E.H., Yu, T.K., Rhee, S., and Suh, J.Y.** (2014). Structural basis for the auxin-induced transcriptional regulation by Aux/IAA17. Proceedings of the National Academy of Sciences of the United States of America **111,** 18613-18618.

**Nanao, M.H., Vinos-Poyo, T., Brunoud, G., Thevenon, E., Mazzoleni, M., Mast, D., Laine, S., Wang, S., Hagen, G., Li, H., Guilfoyle, T.J., Parcy, F., Vernoux, T., and Dumas, R.** (2014). Structural basis for oligomerization of auxin transcriptional regulators. Nature communications **5,** 3617.
